# Prevalence and intensity of soil-transmitted helminth infections of children in sub-Saharan Africa, 2000–18: a geospatial analysis

**DOI:** 10.1016/S2214-109X(20)30398-3

**Published:** 2020-12-15

**Authors:** Benn Sartorius, Jorge Cano, Hope Simpson, Lucy S Tusting, Laurie B Marczak, Molly K Miller-Petrie, Boniface Kinvi, Honorat Zoure, Pauline Mwinzi, Simon I Hay, Maria Rebollo, Rachel L Pullan

**Affiliations:** a Department of Disease Control, London School of Hygiene and Tropical Medicine, London, UK; b Centre on Climate Change and Planetary Health, London School of Hygiene and Tropical Medicine, London, UK; c Department of Health Metrics Sciences, University of Washington, Seattle, WA, USA; d Institute for Health Metrics and Evaluation, University of Washington, Seattle, WA, USA; e Department of Environmental and Occupational Health Sciences, University of Washington, Seattle, WA, USA; f Department of Public Health Medicine, University of KwaZulu-Natal, Durban, South Africa; g Expanded Special Project for Elimination of Neglected Tropical Diseases, WHO Regional Office for Africa, Brazzaville, Democratic Republic of the Congo

## Abstract

**Background:**

Driven by global targets to eliminate soil-transmitted helminths as a public health problem, governments have rapidly rolled out control programmes using school and community-based platforms. To justify and target ongoing investment, quantification of impact and identification of remaining high-risk areas are needed. We aimed to assess regional progress towards these targets.

**Methods:**

We did a continental-scale ecological analysis using a Bayesian space–time hierarchical model to estimate the effects of known environmental, socioeconomic, and control-related factors on the prevalence of soil-transmitted helminths, and we mapped the probability that implementation units had achieved moderate-to-heavy intensity infection prevalence of less than 2% among children aged 5–14 years between Jan 1, 2000, and Dec 31, 2018.

**Findings:**

We incorporated data from 26 304 georeferenced surveys, spanning 3096 (60%) of the 5183 programmatic implementation units. Our findings suggest a reduction in the prevalence of soil-transmitted helminths in children aged 5–14 years in sub-Saharan Africa, from 44% in 2000 to 13% in 2018, driven by sustained delivery of preventive chemotherapy, improved sanitation, and economic development. Nevertheless, 1301 (25%) of 5183 implementation units still had an estimated prevalence of moderate-to-heavy intensity infection exceeding the 2% target threshold in 2018, largely concentrated in nine countries (in 1026 [79%] of 1301 implementation units): Nigeria, Democratic Republic of the Congo, Ethiopia, Cameroon, Angola, Mozambique, Madagascar, Equatorial Guinea, and Gabon.

**Interpretation:**

Our estimates highlight the areas to target and strengthen interventions, and the areas where data gaps remain. If elimination of soil-transmitted helminths as a public health problem is to be achieved in sub-Saharan Africa by 2030, continued investment in treatment and prevention activities are essential to ensure that no areas are left behind.

**Funding:**

Bill & Melinda Gates Foundation.

## Introduction

Soil-transmitted helminths (including *Ascaris lumbricoides, Trichuris trichiura*, and the hookworms *Ancylostoma duodenale* and *Necator americanus)* are among the most widespread of the neglected tropical diseases (NTDs).^1^, ^2^, ^3^ In 2010, soil-transmitted helminths were estimated to infect more than a billion people.^2^, ^4^, ^5^ The past decade has seen substantial progress in the roll out of control measures for soil-transmitted helminths, driven by strengthened collaboration and country commitment and expanded donor support. During this time, the WHO 2020 NTD Roadmap target has been to provide regular anthelmintic treatment to at least 75% of children in settings where infection prevalence in children aged 5–14 years exceeds 20%. By 2018, this goal was nearly met in sub-Saharan Africa, with an estimated 70% of at-risk children receiving regular deworming.^5^ The new WHO 2030 NTD Roadmap now looks beyond coverage, with a goal of eliminating morbidity associated with soil-transmitted helminths (ie, elimination as a public health problem, defined as a moderate-to-heavy intensity infection prevalence of <2%).^6^ Although deworming through mass treatment platforms is known to be a highly cost-effective public health intervention,^7^, ^8^ little is known regarding the effects of mass treatment on the changing prevalence and intensity of soil-transmitted helminths in children. Such an evaluation has been hampered because, in many endemic countries, control was initiated in the absence of comprehensive baseline data—either due to resource constraints or because anthelmintics were already being delivered through other programmes—and surveys have not been done.

In 2016, the WHO Africa Regional Office established the Expanded Special Project to Eliminate NTDs (ESPEN) to provide technical support to national NTD programmes. ESPEN includes the development of a pioneering open-access platform that enables health ministries and stakeholders to share, synthesise, and evaluate subnational programmatic data on multiple NTDs. Here, we incorporate epidemiological and programmatic data collated through this platform into a robust space–time modelling framework, with the aim of producing programmatic implementation unit-level estimates of the prevalence of soil-transmitted helminths infections among children aged 5–14 years across sub-Saharan Africa in 2000–18.

**Research in context**

**Evidence before this study**

Global and national estimates of soil-transmitted helminth burden, incorporating data up to 2010, estimated the prevalence of hookworm in sub-Saharan Africa at 13·6%, *Ascaris lumbricoides* at 13·6%, and *Trichuris trichiura* at 11·6%. Estimates for sub-Saharan Africa have been subsequently updated using a geostatistical approach to include additional datapoints up to 2012, resulting in period estimates (before 2000 and from and including 2000) at high resolution. Although these estimates suggested reductions in soil-transmitted helminth infections, their utility for tracking control targets remains limited. To our knowledge, no previous estimates have quantified the effects of preventive chemotherapy and other key determinants of soil-transmitted helminths at implementation unit-year level or estimated the prevalence of moderate-to-heavy intensity infection at scale.

**Added value of this study**

This study provides estimates of the prevalence of moderate-to-heavy intensity soil-transmitted helminths at the implementation unit level to track progress toward the soil-transmitted helminth goals for 2020 and 2030 and help identify areas at highest risk of not attaining target thresholds. We used a georeferenced database of soil-transmitted helminth prevalence for sub-Saharan Africa, to which we applied a Bayesian space–time model that included key time-varying determinants. We estimate prevalence (with CIs) of infection, and of moderate-to-heavy intensity infection, for each soil-transmitted helminth species by implementation unit among children in 2000–18. Our model improves upon previous efforts by using a longitudinal perspective not included in earlier national or regional estimates, accounting for subnational variation in space–time, and including time-varying preventive chemotherapy, water and sanitation, and socioeconomic patterns. We also include summaries for each country to increase the accessibility to and transparency of our estimates.

**Implications of all the available evidence**

This space–time analysis quantifies progress towards elimination in 2000–18. Although the prevalence of soil-transmitted helminths has substantially reduced over the past two decades, with consideration of a revised control strategy warranted in many areas, it is still highly unlikely that all areas will achieve the global targets for 2020 and beyond. Our estimates can be used by national programmes and implementing partners to identify areas likely to require reassessment surveys, and areas that still require surveillance or intervention to better target preventive chemotherapy to children at high risk of morbidity and to enhance access to at least basic sanitation. If elimination of soil-transmitted helminths as a public health problem is to be achieved by 2030, better resource allocation and renewed and increased investment in treatment and prevention activities are urgently required to accelerate reductions and ensure that no subpopulations are left behind.

## Methods

### Study design and participants

We did continental-scale ecological analysis using a Bayesian space–time hierarchical model to estimate the effects of known environmental, socioeconomic, and control-related factors on the prevalence of soil-transmitted helminths, and we mapped the probability that implementation units had achieved the target moderate-to-heavy intensity infection prevalence of less than 2% among children aged 5–14 years between Jan 1, 2000, and Dec 31, 2018.

This analysis adhered to Guidelines for Accurate and Transparent Health Estimates Reporting standards (appendix pp 52–53).^9^ Data and covariates used are all in the public domain (appendix pp 60–62).

### Procedures

Survey data on the prevalence of soil-transmitted helminth infections (including *A lumbricoides, T trichiura*, and hookworm) were obtained from the publicly available ESPEN database (last accessed July 14, 2020). This database incorporates data shared with WHO by national governments through standard reporting routes, and prevalence estimates from research surveys done in representative populations abstracted by the Global Atlas of Helminth Infections project.^10^ Datapoints were linked to programmatic implementation units (typically district level) using geographical coordinates or, in their absence, the reported implementation unit name. Analysis was restricted to surveys done between 2000 and 2018, and survey data contained within the likely spatial limits of soil-transmitted helminths in sub-Saharan Africa.^11^ Datapoints that had either a missing year of survey or could not be geocoded to a particular implementation unit were excluded from the analyses (appendix pp 54–55).

The intensity of soil-transmitted helminth transmission is linked to multiple environmental and human factors that affect the survival of, and exposure to, helminth infective life stages. To inform our model, we compiled a suite of environmental,^12^ control-related, and socioeconomic covariates (appendix pp 60–62),^13^ which included high-resolution meteorological and environmental data (aridity, maximum temperature, soil composition, and pH of topsoil), gridded estimates of relevant socioeconomic and household factors (living in slum conditions, poor access to safe drinking water, poor access to sanitation facilities, and gross domestic product at purchasing power parity [GDP PPP]), and anthelmintic preventive chemotherapy data at implementation unit level provided routinely by national programmes to ESPEN (school-based deworming for soil-transmitted helminths and mass drug administration for lymphatic filariasis). A detailed description of the covariate data can be found in the appendix (pp 60–62).

Covariate data were linked by geographical location and year to the available survey data on soil-transmitted helminth prevalence using geographical coordinates. For implementation units without soil-transmitted helminth survey data, we took the median value of a given covariate as the summary value for a given implementation unit (and year, if time varying) for inclusion in the model.

### Statistical analysis

We assessed bivariate associations between covariates and prevalence of each species. Covariates that were most predictive and non-collinear were retained in the full model. Before inclusion in the joint multivariable modelling framework, we used a principled basis to assess pairwise comparison of the prevalence of soil-transmitted helminths against each selected covariate for presence of prominent outliers using a Kernel Density Estimation Outlier Score algorithm,^14^ implemented in R (version 4.0.0), and we excluded highly influential outliers from the analytic dataset (appendix p 65).

We formulated our model within a Bayesian hierarchical framework using a shared component space–time model,^15^, ^16^ which simultaneously analyses the three soil-transmitted helminth species, through additive decomposition for the shared components (both spatially and temporally), and includes space–time interaction terms for each species (*A lumbricoides, T trichiura*, and hookworm) and all combined to more accurately predict prevalence at locations where survey data were absent. The observed number of positive tests (and total number of children tested) for each soil-transmitted helminth species and for all species combined for each implementation unit (i=1...,5183) and year (j=2000…,2018) were assumed to follow binomial distributions. We fitted this model in WINBUGS (version 1.4.3) using Markov chain Monte Carlo simulation and non-informative priors. The full specification and model code can be found in the appendix (pp 77–81).

To estimate the prevalence of moderate-to-heavy intensity infection, we used data from a systematic review of 19 surveys in 10 countries that assessed the species-specific relationships between infection prevalence and infection intensity before any treatment intervention.^17^ These data suggest very strong, species-specific nonlinear relationships between prevalence and intensity of infection; we confirmed these relationships were consistent after treatment using independent data from a large cluster-randomised trial done in Kenya (appendix p 64).^18^ The posterior prevalence for each soil-transmitted helminth by implementation unit and year were linked to the corresponding estimated prevalence of moderate-to-heavy intensity infection (appendix p 63). We estimated exceedance probabilities for the 20% any-prevalence threshold and 2% moderate-to-heavy intensity infection threshold. We used Richardson's criterion^19^ to assess significance of threshold breach, in which Bayesian exceedance probabilities in excess of 0·80 were deemed to be significant.

To estimate population counts of infection and moderate-to-heavy intensity infection by species, we multiplied posterior prevalence estimates by the estimated population of children aged 5–14 years.^20^ Population size by implementation unit and year were used to generate population-weighted prevalence estimates at national and regional levels. We did counterfactual analyses^21^ to estimate the individual contributions that preventive chemotherapy, improvements in sanitation, and poverty reduction made to the changing burden of soil-transmitted helminths across sub-Saharan Africa. Model coefficients from the full multivariable space–time joint model were reversed, with all other covariates taking their observed value, and added back to the natural logarithm of the posterior estimated prevalence and exponentiated to yield the estimated counterfactual prevalence in a given implementation unit and year. Because the programmatic data used in this study are largely from baseline surveys (before deworming), the estimated coefficients for the impact of cumulative effective mass drug administration is underestimated when compared with an external source from Kenya.^22^ We therefore applied a correction factor to the estimated coefficient for this covariate to more accurately quantify its counterfactual contribution.

We used two-chain Markov chain Monte Carlo simulation to assess model convergence (appendix p 82). For model validation, we compared the observed and fitted prevalence values to assess overall model fit (appendix pp 83–85) and did out-of-sample validation by withholding a random 20% sample of observed data and fitting the model to the remaining data to predict for this withheld sample (appendix p 86). Model predictive ability was measured by quantifying the proportion of correctly predicted values within the kth highest posterior density interval varying from 50% to 95% and using Bland-Altman plots and Lin's Concordance Correlation Coefficient of Absolute Agreement (appendix pp 83–85).

Statistical code is available in the appendix (pp 79–81). Analyses were done using Stata 16, R (version 4.0.0) and WINBUGS (version 1.4.3).

### Role of the funding source

The funder of the study had no role in study design, data collection, data analysis, data interpretation, or writing of the report. The corresponding author had full access to all the data in the study and had final responsibility for the decision to submit for publication.

## Results

As of July 14, 2020, the ESPEN database contained 30 732 datapoints in sub-Saharan Africa, with 27 171 datapoints from between Jan 1, 2000, and Dec 31, 2018 (2236 datapoints had a missing year and 1325 were from before 2000), of which 26 304 (97%) could be geolocated to a specific coordinate or accurately assigned to a programmatic implementation unit (appendix p 55). 24 496 datapoints (93%) were from studies conducted since 2005 and were based on quantification of eggs in stool using Kato-Katz (appendix p 55) and, of the 12879 datapoints or surveys with a reported age range, 8588 (67%) were among children aged 5–14 years (appendix pp 56–59). The 26 304 georeferenced datapoints, when aggregated by implementation unit, represent 5021 implementation unit-years of data covering 3096 (60%) of the 5183 implementation units. Temporal coverage ranged from a minimum of 46 survey datapoints in 2000 to a maximum of 4860 datapoints (spanning 840 implementation units) in 2013. Further geographical and temporal details of data and age profiles of the surveys are available in the appendix (pp 54–59).

Estimated uncertainty overall and by year is driven by underlying data density. Countries where future sampling might need to be prioritised to allow for more precise estimates in the period leading up to the global targets are listed in the appendix (pp 66–68). Central African Republic, Congo, Djibouti, Equatorial Guinea, and Somalia, for example, had no available datapoints for 2000–18, whereas Guinea-Bissau and Lesotho had 50 or fewer datapoints in total over this period (appendix pp 66–67). This small amount of data resulted in larger CIs for estimates arising in these countries (appendix pp 67–68). Full details of the adequacy of model fit and out of sample predictive validity are presented in the appendix (pp 82–86). Out-of-sample validation suggests that 742 (99%) of the 752 observed prevalence values were contained in the 95% CI for the posterior prediction. Furthermore, there was high correlation between model predicted versus observed prevalence values in this withheld sample (Spearman correlation coefficient 0·67; p<0·0001).

**Figure 1:**
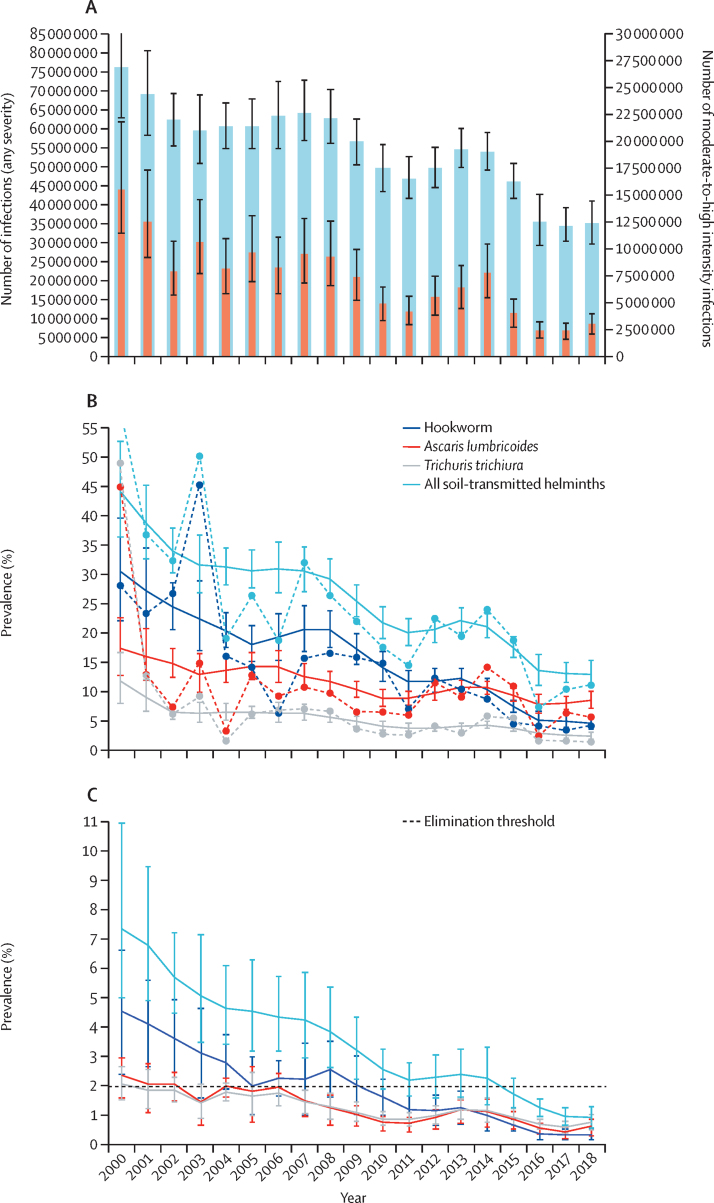
Prevalence of any soil-transmitted helminth infections (A) and of moderate-to-high intensity infections (B), and the absolute number of prevalent cases (C), in children aged 5–14 years in sub-Saharan Africa, 2000–18 Data are shown with 95% CIs. (A) Dotted lines with circle markers represent the raw aggregated prevalence in the underlying input data. (C) Blue bars represent any infections, and red bars represent moderate-to-high intensity infections.

**Figure 2:**
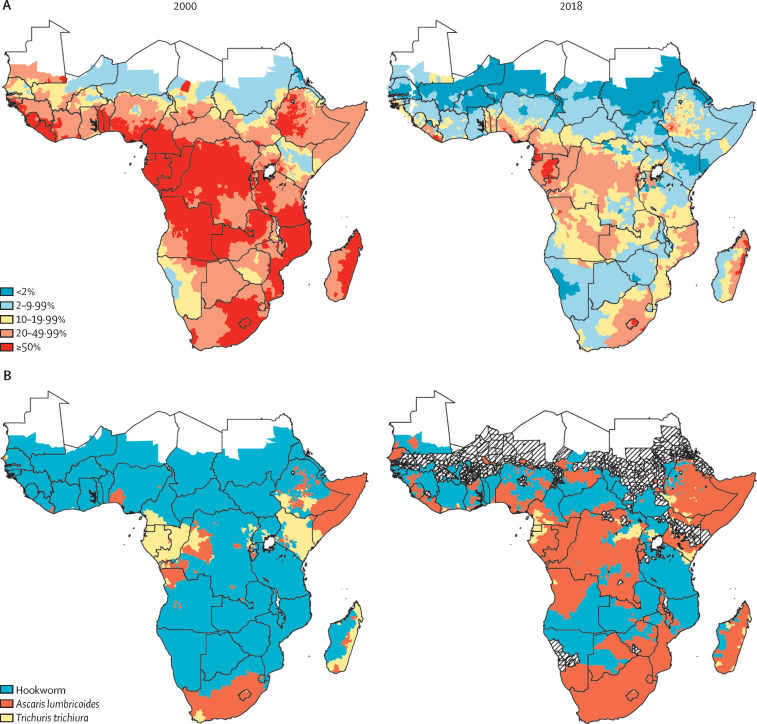
Prevalence of any soil-transmitted helminth infection (A) and predominant soil-transmitted helminth species (B), by implementation unit, in 2000 and 2018 Predominant species was only assessed if the overall prevalence of soil-transmitted helminths was ≥2%.

Our model suggests that, in 2000, before the upscaling of NTD control programmes across much of sub-Saharan Africa, soil-transmitted helminths were widespread, with an estimated 76·5 million (95% CI 63·1 million to 91·5 million) prevalent cases among school-aged children (figure 1A), and 4290 (83%) of 5183 implementation units exceeding the 20% prevalence threshold, 2589 (60%) of 4290 with high probability (exceedance p>0·80; figure 2A). Since 2000, there has been a substantial decrease in the geographic extent and burden of soil-transmitted helminths, from an estimated population-weighted prevalence of 44% in children aged 5–14 years in 2000 to 13% by 2018, meaning there were an estimated 35·0 million (95% CI 29·5 million to 41·1 million) prevalent cases among children aged 5–14 years in 2018. By 2018, the majority of implementation units that still had a prevalence of more than 20% were concentrated in four countries: Nigeria (247 implementation units), Democratic Republic of the Congo (DRC; 241 implementation units), Ethiopia (132 implementation units), and Cameroon (112 implementation units; appendix pp 69–73).

The largest and most consistent reduction over the 2000–18 period was for hookworm (from 30% to 5%) with more modest reductions for *A lumbricoides* (17% to 9%) and *T trichiura* (12% to 2%; figure 1B). By 2018, *A lumbricoides* prevalence had marginally surpassed hookworm prevalence at continental scale, replacing hookworm as the most prevalent soil-transmitted helminths in most of central Africa (eg, Congo and DRC), parts of western African (eg, Nigeria), and much of Ethiopia, Madagascar, Zimbabwe, and southern Africa (figure 2). The spatial extent of *T trichiura* also shrank slightly over the study period, with focal transmission remaining high along coastlines in Equatorial Guinea, Gabon, Cameroon and Kenya, with additional inland focal transmission areas in DRC, Uganda, and Ethiopia (figure 2B).

**Figure 3:**
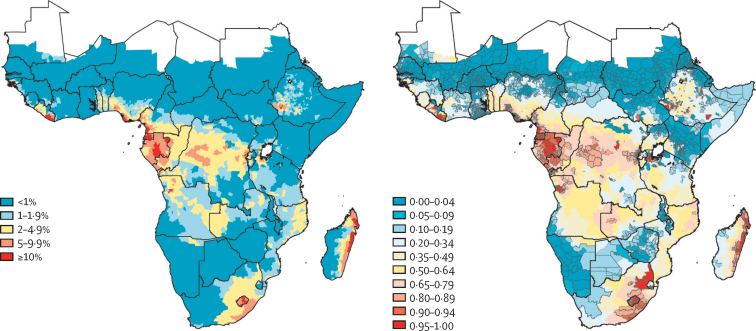
Prevalence of moderate-to-heavy intensity infection with soil-transmitted helminths, by implementation unit, in 2018 (A), and probability that a given implementation unit had a prevalence of 2% of more (B)

The prevalence of moderate-to-heavy intensity infections in school-aged children in sub-Saharan Africa appears to have reduced from 7·4% in 2000 to 1·0% by 2018 (figure 1C), with an estimated 15·6 million (95% CI 11·5 million to 21·8 million) cases in 2000 reducing to 2·9 million (2·0 million to 3·9 million) cases by 2018 (figure 1A). However, 1301 (25%) of 5183 implementation units still had an estimated prevalence of moderate-to-heavy intensity infection exceeding the 2% target threshold in 2018, largely concentrated in nine countries (in 1026 [79%] of 1301 implementation units): Nigeria, DRC, Ethiopia, Cameroon, Angola, Mozambique, Madagascar, Equatorial Guinea, and Gabon. Our estimates suggest that 3057 (72%) of 4264 implementation units previously above 2% prevalence of moderate-to-heavy intensity infections in 2000 had attained the 2% target threshold by 2018, based on posterior median prevalence (figure 3A; appendix pp 74–76). However, 862 (28%) of 3057 implementation units were estimated to be significantly below the 2% threshold in 2018, based on exceedance probability (>0·80; figure 3B).

**Figure 4:**
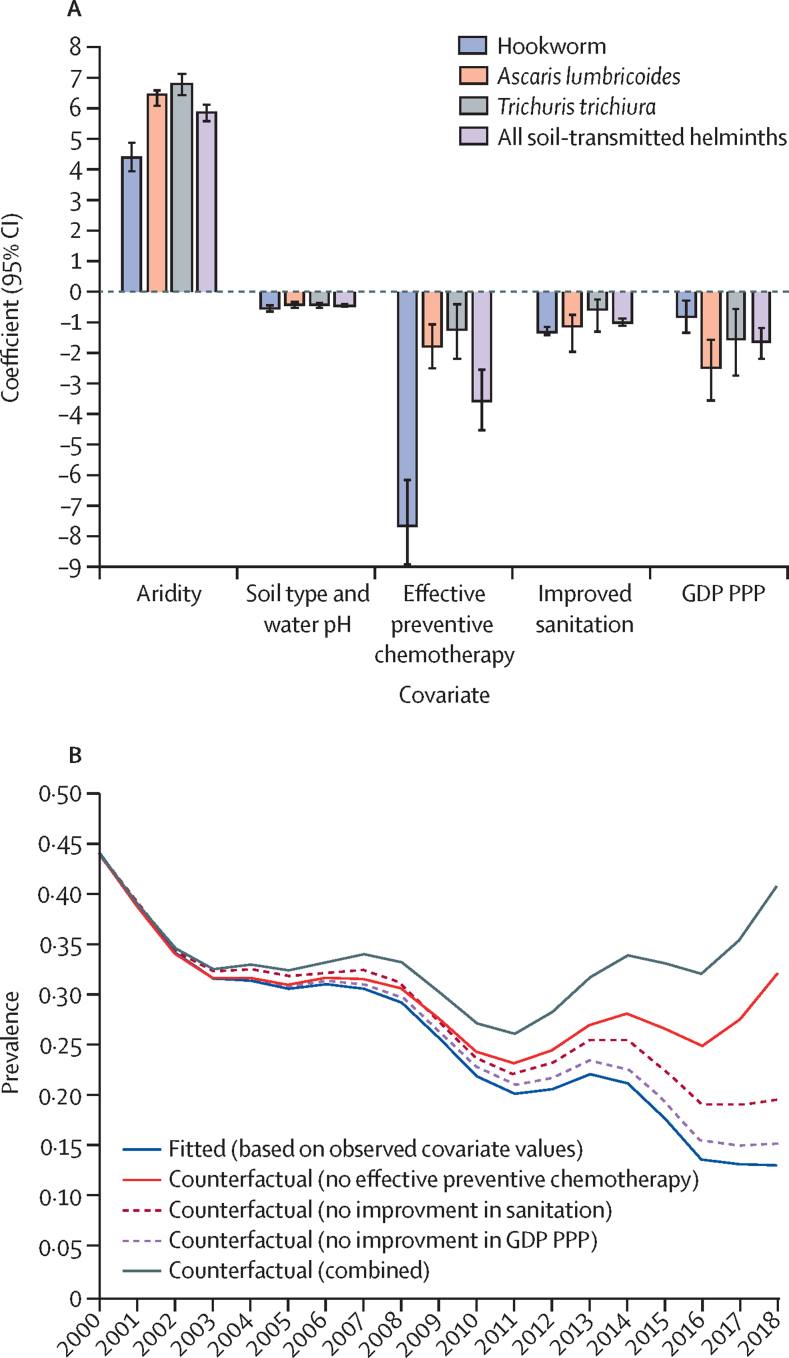
Association between key indicators and the impact of interventions and development and prevalence of soil-transmitted helminth infections among children aged 5–14 years in sub-Saharan Africa, 2000–18 (A) Coefficients and 95% CIs for the multivariable risk factor analysis. (B) Time series of predicted population-weighted prevalence of soil-transmitted helminths for sub-Saharan Africa (blue line) and counterfactual predictions (red and black lines), assuming no effective mass drug administrations, changes in GDP PPP from baseline (2000), or improved sanitation coverage, by implementation unit. GDP PPP=gross domestic product at purchasing power parity.

The data suggest a strong positive association between increasing aridity index (that is, less arid environments) and all three soil-transmitted helminth species (figure 4A). Decreasing sand content in the soil and ground water pH were negatively associated with prevalence of soil-transmitted helminths. Increasing cumulative number of anthelmintic mass treatment rounds, access to improved sanitation, and increased GDP PPP all had significant reductive effects on prevalence of soil-transmitted helminths, with mass treatment having a greater effect for hookworm compared with *A lumbricoides* and *T trichiura* (figure 4A). Similarly, improved sanitation access had a marginally stronger reductive effect on hookworm compared with the other two species.

On the basis of the counterfactual analysis, cumulative rounds of anthelmintic mass treatment had the largest contribution to reductions in the prevalence of soil-transmitted helminth infection over the study period, followed by improved sanitation and economic development (figure 4B). The counterfactual scenario suggests that if there had been no mass treatment or improvements in access to sanitation or economic development, prevalence of soil-transmitted helminths among school-aged children in sub-Saharan Africa would have been around triple the estimated value by 2018.

The majority of high burden implementation units (estimated soil-transmitted helminths prevalence in 2018 exceeding 10%) in the bottom tenth percentile for key socioeconomic (sanitation and GDP) and intervention indicators were concentrated in DRC, Angola, Ethiopia, and Madagascar (appendix pp 3–4). By 2018, preventive chemotherapy through school-based deworming or other mass drug administration interventions had not yet been reported to the WHO for 309 (23%) of 1359 implementation units where prevalence was still predicted to exceed 20% (60 in DRC, 55 in Angola, 52 in Nigeria, and 46 in Gabon; appendix p 71).

## Discussion

Our estimates of soil-transmitted helminth prevalence and intensity among children in sub-Saharan Africa highlight substantial differences in the changing burden of infection between and within countries. By 2018, an estimated 35·0 million children aged 5–14 years were infected with at least one soil-transmitted helminth species, with an estimated 2·9 million still harbouring moderate-to-heavy intensity infections, predominantly in a band stretching from southern Nigeria through central Africa, western-central Ethiopia, and areas of southern Africa and eastern Madagascar. Nevertheless, these estimates reflect substantial reductions in risk over the past two decades, with almost 72% of endemic implementation units predicted to have achieved the WHO 2030 target of bringing prevalence of moderate-to-heavy intensity infections in children to less than 2%. Our results suggest that reductions in the spatial extent and burden of soil-transmitted helminths were driven by multiple factors, most notably the scale up of preventive chemotherapy through both lymphatic filariasis and soil-transmitted helminth control programmes, and by improvements in access to sanitation. Crucially, results highlight remaining areas with inadequate coverage or impact, providing an evidence base to support the direction of resources to areas of greatest need.

Historically, there has been a notable absence of standardised, subnational treatment coverage data for deworming programmes. Compounded by an absence of impact assessment surveys in many settings, this absence of data has prevented large-scale evaluation of programme impact and progress towards WHO targets. Incorporating available survey data and treatment data reported by Ministries of Health to WHO, and now made available through the WHO ESPEN data portal, within a geospatial framework has clearly shown the substantial cumulative effect school-based deworming and lymphatic filariasis preventive chemotherapy campaigns had on the burden soil-transmitted helminths, in broad agreement with analysis of data from sentinel sites across multiple countries.^23^ This approach also supports the identification of regions that might benefit from tailored or enhanced intervention packages (appendix p 87). For example, inclusion of ivermectin within preventive chemotherapy regimens will augment treatment efficacy in areas with persistent *T trichiura*,^24^ whereas expansion of deworming programmes to include adults in hookworm dominated areas and increasing to biannual frequency in *A lumbricoides* dominated areas might have larger effects.^25^, ^26^, ^27^

Although results suggest that mass preventive chemotherapy has led to substantial reductions in prevalence, it is unlikely to eliminate soil-transmitted helminths as a public health problem in settings where moderate-to-heavy intensity infection remains above target thresholds and where reinfection through environmental reservoirs of soil-transmitted helminths eggs remain high.^28^ Our ecological analysis suggests concomitant improvement in access to sanitation has acted to substantially reduce the prevalence of soil-transmitted helminth infection, probably by reducing soil contamination and the risk of reinfection after treatment.^29^ The 2030 WHO Roadmap additionally identifies the crucial role water, sanitation, and hygiene has in the control of soil-transmitted helminth infection and persistence,^30^ and our observation further emphasises the urgent need to identify and evaluate context-specific, feasible complementary sanitation and hygiene interventions to best support deworming programmes.^31^, ^32^

Our results highlight key gaps in empirical data across the continent. Specifically, we found high uncertainty around estimates in central Africa, Guinea, Ethiopia, Mozambique, South Africa, and Madagascar, which include areas with the greatest predicted burden of infection, driven by an absence of survey data. Furthermore, although an increasing density of empirical data by year has reduced overall uncertainty around continental prevalence estimates, the majority of data included in our analysis were collected before the advent of mass treatment campaigns. Many countries are now planning impact assessment surveys and reduce the frequency of mass treatment campaigns, having implemented 5 years or more of preventive chemotherapy, and guidance is required to inform their appropriate design. Our maps can help identify areas most likely to have reached a prevalence of soil-transmitted helminths of less than 10% (which includes an estimated 331 implementation units) and thus indicated by the WHO as being suitable for reassessment.^33^ Conversely, our model also identified many implementation units (n=399) that have implemented 5 years or more of preventive chemotherapy but not attained the 10% prevalence threshold.

This study has several limitations. First, for many parts of sub-Saharan Africa, input data were absent or insufficient in space–time, yielding larger CIs. We also cannot discount that our estimates might have been systematically biased if survey sites in certain locations were chosen on the basis of high-risk of infection, potentially overestimating the burden of soil-transmitted helminths in certain areas. Most of the input data were from pre-intervention mapping surveys and there is considerably less information from impact evaluation surveys, which might have upwardly biased estimates of moderate-to-heavy intensity infection prevalence. Conversely, in several locations, soil-transmitted helminths baseline surveys were done after the start of mass drug administration for lymphatic filariasis, which could have distorted the modelled association with key covariates in these areas. Second, although we provide estimates of the prevalence of moderate-to-heavy intensity infection, it is estimated from the prevalence of any infection using evidence from peer-reviewed literature, owing to the absence or scarcity of reports of infection intensity in existing survey data. Third, although we have included covariates, this ecological modeling framework could lead to residual confounding. Results in off-sample implementation units are also vulnerable to inaccuracies in the used covariate layers. Fourth, we cannot discount the potential incompleteness of deworming data due to unreported or non-programmatic deworming and deworming coverage through child health days and antenatal care. Finally, the prevalence of soil-transmitted helminths is estimated using coprological approaches, primarily Kato-Katz. Although these approaches are sufficiently sensitive at higher prevalence (for example, during planning and initial evaluation phases of soil-transmitted helminths programmes), sensitivity decreases as intensity of infection reduces, especially for hookworm.^34^ This might have contributed to an overestimation of the reduction in the burden of soil-transmitted helminths.

Our estimates highlight the high heterogeneity within the region and within countries, with further variability in the rates of reduction in burden. Although much of the region has made admirable progress towards achieving elimination of soil-transmitted helminths as a public health problem, many of the gains observed over the last decade will be lost if preventive chemotherapy interventions are stopped too soon or interrupted, especially in the context of COVID-19. To support continued investment in control, it is essential that our findings are confirmed empirically using a robust impact assessment framework. Our estimates provide an updated tool for researchers, policymakers, and programme implementers when prioritising areas requiring assessment to assess localised needs and to more efficiently target interventions.

## Data sharing

The study prevalence data are also available online. The online sources for the covariate data are provided in the appendix (pp 60–62).
