# Supplementary material for: Prevalence and intensity of soil-transmitted helminth infections of children in sub-Saharan Africa, 2000–18: a geospatial analysis
Source: Lancet Glob Health. 2020 Dec 15;9(1):e52–60. doi: 10.1016/S2214-109X(20)30398-3 (PMC7786448; doi:10.1016/S2214-109X(20)30398-3)
Supplement: Supplementary appendix [file mmc1.pdf]

THE LANCET
Global Health

Supplementary appendix

This appendix formed part of the original submission and has been peer reviewed. We post it as supplied by the authors.

Supplement to: Sartorius B, Cano J, Simpson H, et al. Prevalence and intensity of soil-transmitted helminth infections of children in sub-Saharan Africa, 2000–18: a geospatial analysis. *Lancet Glob Health* 2021; **9:** e52–60.

Supplementary Material

Section A: Country specific STH profiles

A1: Key STH indicators by country in 2018

ISO 3	Total number of IU's	National STH prevalence, 2018	National moderate-to-heavy intensity STH prevalence, 2018	Number of IU's with STH prevalence ≥ 20%, 2018	Proportion of IU's with STH prevalence ≥ 20%, 2018	Number of IU's above elimination target of 2% prevalence for moderate-to-heavy intensity, 2018	Proportion of IU's above elimination target of 2% prevalence for moderate-to-heavy intensity, 2018	Proportion of IUs in lowest 10 percentile i, ii for cumulative effective PC rounds, 2018	Proportion of IUs in lowest 10 percentile i for improved sanitation, 2018	Proportion of IUs in lowest 10 percentile i for slum-like living conditions, 2018	Proportion of IUs in lowest 10 percentile i for GDP PPP, 2018
AGO	164	20.4%	2.2%	88	53.7%	80	48.8%	62.8%	0.0%	51.2%	6.7%
BDI	46	15.8%	1.5%	15	32.6%	14	30.4%	0.0%	0.0%	0.0%	0.0%
BEN	77	15.4%	1.4%	15	19.5%	14	18.2%	7.8%	0.0%	0.0%	0.0%
BFA	70	1.4%	0.0%	0	0.0%	0	0.0%	0.0%	0.0%	0.0%	8.6%
BWA	24	6.5%	0.4%	0	0.0%	0	0.0%	91.7%	0.0%	0.0%	8.3%
CAF	17	13.3%	1.1%	1	5.9%	1	5.9%	52.9%	0.0%	11.8%	94.1%
CIV	83	8.0%	0.5%	1	1.2%	0	0.0%	8.4%	0.0%	0.0%	0.0%
CMR	189	20.6%	2.8%	112	59.3%	109	57.7%	0.5%	0.0%	0.0%	2.6%
COD	516	19.8%	2.2%	241	46.7%	234	45.3%	29.5%	0.0%	39.9%	36.4%
COG	43	29.4%	3.9%	31	72.1%	31	72.1%	4.7%	0.0%	0.0%	9.3%
DJI	5	3.2%	0.1%	0	0.0%	0	0.0%	100.0%	0.0%	20.0%	40.0%
ERI	58	0.3%	0.0%	0	0.0%	0	0.0%	79.3%	0.0%	0.0%	8.6%
ETH	744	14.9%	1.4%	132	17.7%	123	16.5%	26.5%	58.9%	14.8%	3.5%
GAB	51	43.0%	7.0%	51	100.0%	50	98.0%	90.2%	0.0%	0.0%	0.0%
GHA	216	4.4%	0.2%	0	0.0%	0	0.0%	6.0%	0.0%	0.0%	0.0%
GIN	38	11.0%	0.9%	4	10.5%	4	10.5%	28.9%	0.0%	0.0%	26.3%
GMB	44	7.9%	0.6%	2	4.5%	2	4.5%	100.0%	0.0%	0.0%	0.0%
GNB	118	18.4%	2.2%	55	46.6%	55	46.6%	15.3%	0.0%	0.8%	0.0%
GNQ	17	72.9%	26.9%	17	100.0%	17	100.0%	100.0%	0.0%	0.0%	0.0%
KEN	290	9.0%	0.7%	28	9.7%	23	7.9%	57.2%	0.3%	0.3%	5.2%
LBR	15	24.8%	4.0%	11	73.3%	10	66.7%	6.7%	0.0%	0.0%	53.3%
LSO	10	54.7%	11.3%	10	100.0%	10	100.0%	50.0%	0.0%	0.0%	0.0%
MDG	114	30.9%	5.3%	68	59.6%	67	58.8%	19.3%	23.7%	36.0%	7.0%
MLI	66	1.6%	0.0%	0	0.0%	0	0.0%	1.5%	0.0%	1.5%	36.4%
MOZ	159	18.9%	1.9%	72	45.3%	70	44.0%	1.3%	0.6%	6.9%	39.6%
MRT	42	9.2%	0.7%	0	0.0%	0	0.0%	100.0%	0.0%	4.8%	4.8%
MWI	29	5.0%	0.3%	0	0.0%	0	0.0%	0.0%	0.0%	0.0%	0.0%
NAM	34	7.1%	0.5%	4	11.8%	4	11.8%	100.0%	20.6%	0.0%	11.8%
NER	39	1.2%	0.0%	0	0.0%	0	0.0%	0.0%	5.1%	41.0%	59.0%
NGA	774	15.6%	1.8%	247	31.9%	238	30.7%	15.6%	0.0%	0.0%	0.0%
RWA	30	21.0%	2.8%	13	43.3%	13	43.3%	0.0%	0.0%	0.0%	0.0%
SDN	157	1.2%	0.0%	0	0.0%	0	0.0%	100.0%	0.0%	0.0%	7.6%

SEN	76	5.9%	0.4%	1	1.3%	1	1.3%	1.3%	0.0%	0.0%	0.0%
SLE	14	13.6%	1.2%	3	21.4%	3	21.4%	0.0%	0.0%	0.0%	7.1%
SOM	18	8.8%	0.6%	0	0.0%	0	0.0%	100.0%	0.0%	0.0%	94.4%
SSD	80	4.8%	0.3%	2	2.5%	2	2.5%	91.3%	11.3%	0.0%	2.5%
SWZ	55	10.4%	0.8%	4	7.3%	3	5.5%	52.7%	0.0%	0.0%	0.0%
TCD	91	3.7%	0.2%	0	0.0%	0	0.0%	58.2%	30.8%	29.7%	19.8%
TGO	40	16.6%	1.5%	11	27.5%	11	27.5%	12.5%	0.0%	0.0%	0.0%
TZA	186	13.8%	1.3%	36	19.4%	34	18.3%	0.5%	0.0%	0.0%	19.9%
TZZ	11	18.6%	1.9%	5	45.5%	2	18.2%	0.0%	0.0%	9.1%	9.1%
UGA	116	13.0%	1.1%	15	12.9%	14	12.1%	13.8%	5.2%	0.0%	0.0%
ZAF	52	27.7%	3.7%	31	59.6%	30	57.7%	28.8%	0.0%	0.0%	5.8%
ZMB	103	14.7%	1.3%	33	32.0%	32	31.1%	17.5%	0.0%	14.6%	8.7%
ZWE	62	5.9%	0.3%	0	0.0%	0	0.0%	58.1%	0.0%	0.0%	8.1%

i: based on centile values for SSA in 2018
ii: Only IU's with estimated baseline STH prevalence ≥20%

A2: High burden IU's (estimated prevalence exceeding ≥ 10%) and in the lowest 10 percentile range for key intervention/development indicators (red) in 2018

Lowest cumulative PC, 2018

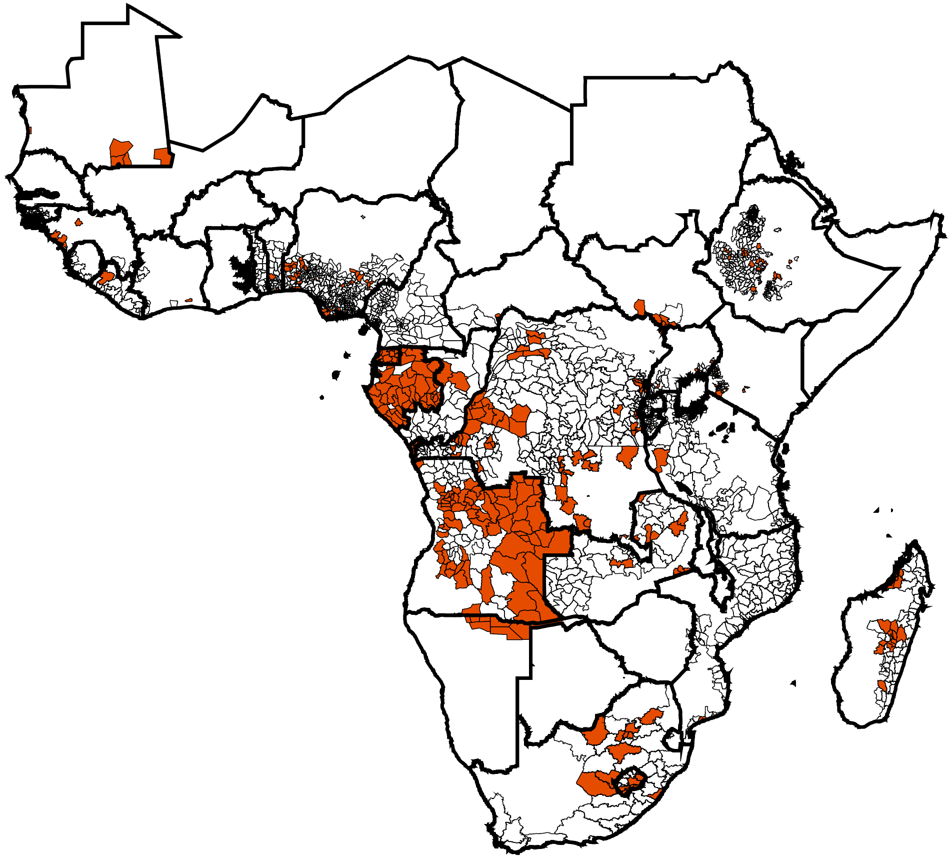

Poor sanitation, 2018

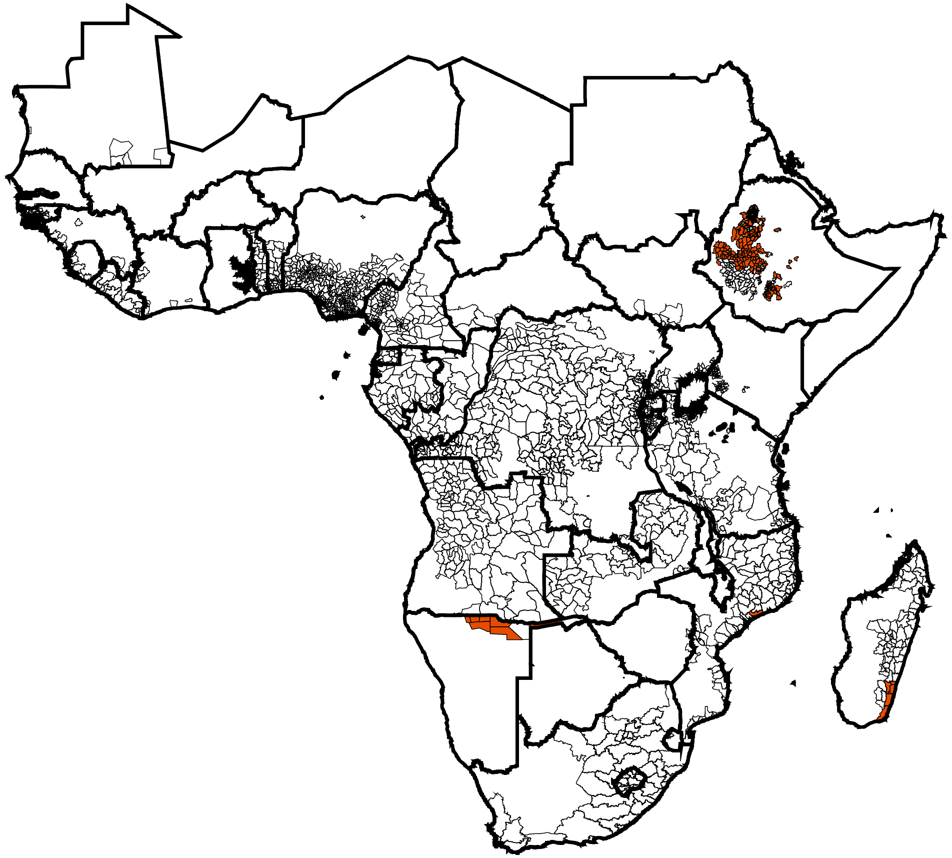

34 Slum living conditions, 2018

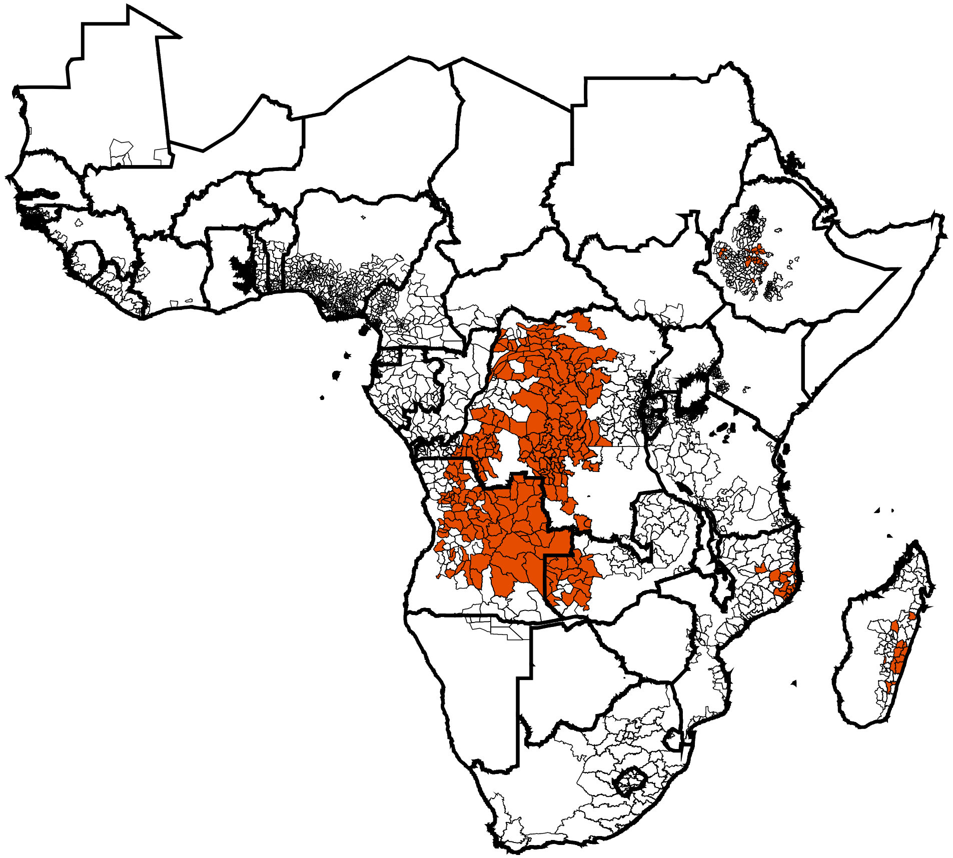

Poverty (GDP PPP), 2018

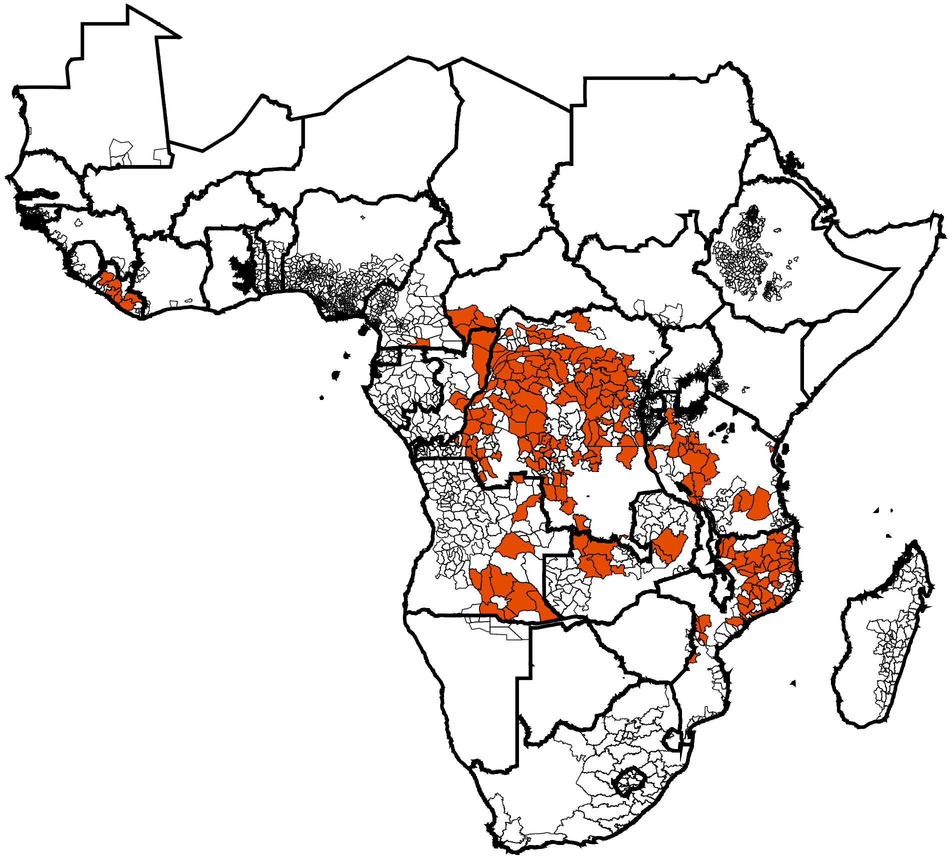

35

A3. Moderate-to-heavy intensity infection by country and exceedance probability uncertainty associated with this threshold, 2018. Note: list of country names and ISO3 codes provided below the table.

ISO3	STH prevalence (any and moderate-to-heavy intensity) by year showing range across IU's using a box plot. Key provided below to aid interpretation:	Prevalence of moderate-to-heavy intensity infection in 2018 at IU level	Probability that a given IU is below the target 2% elimination threshold in 2018 (areas towards red spectrum significantly more likely to exceed the threshold and include solid black outline while those areas towards blue spectrum and in black outline were significantly less likely to exceed aforementioned threshold)
	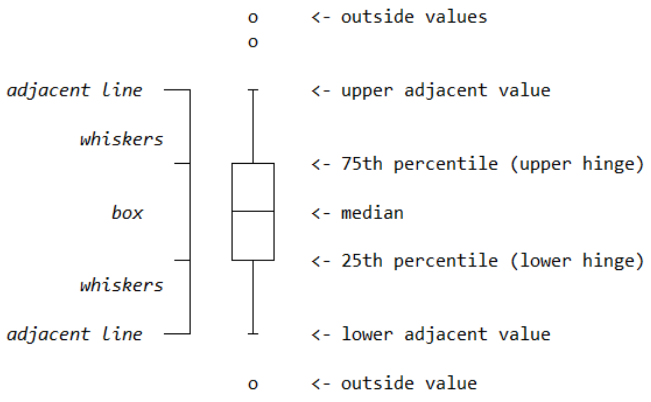	<2%<br>2-4.9%<br>5-9.9%<br>10-14.9%<br>15+%	0.00 - 0.04<br>0.05 - 0.09<br>0.10 - 0.19<br>0.20 - 0.34<br>0.35 - 0.49<br>0.50 - 0.64<br>0.65 - 0.79<br>0.80 - 0.89<br>0.90 - 0.94<br>0.95 - 1.00

AGO

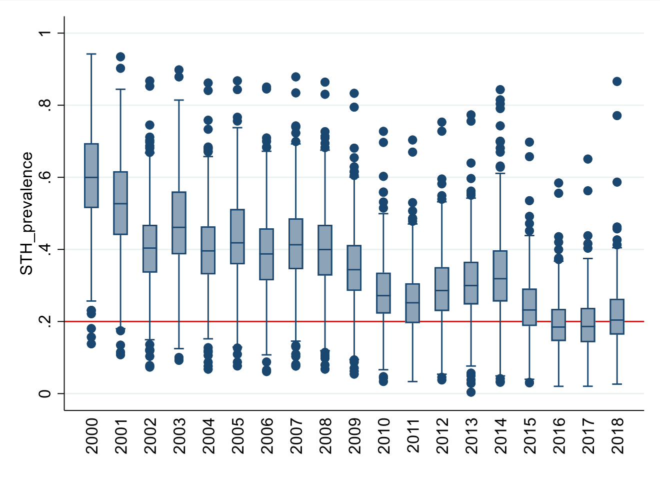
STH_prevalence
0
.2
.4
.6
.8
1
2000 2001 2002 2003 2004 2005 2006 2007 2008 2009 2010 2011 2012 2013 2014 2015 2016 2017 2018

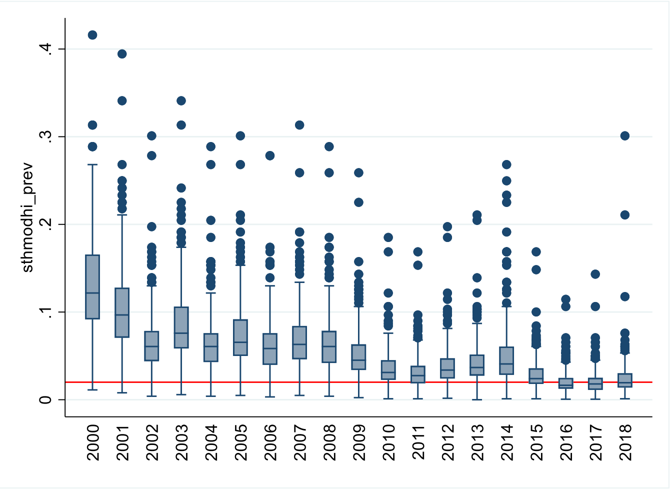
sthmodhi_prev
0
.1
.2
.3
.4
2000 2001 2002 2003 2004 2005 2006 2007 2008 2009 2010 2011 2012 2013 2014 2015 2016 2017 2018

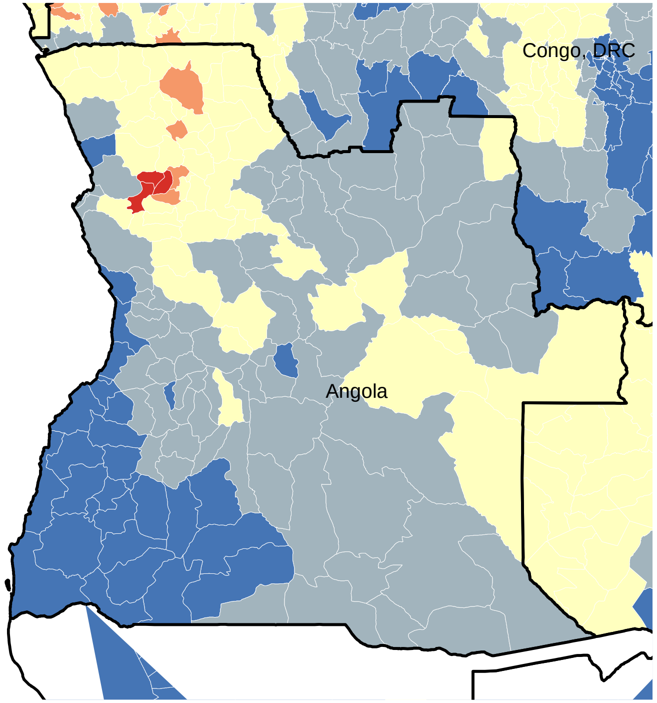
Congo, DRC
Angola

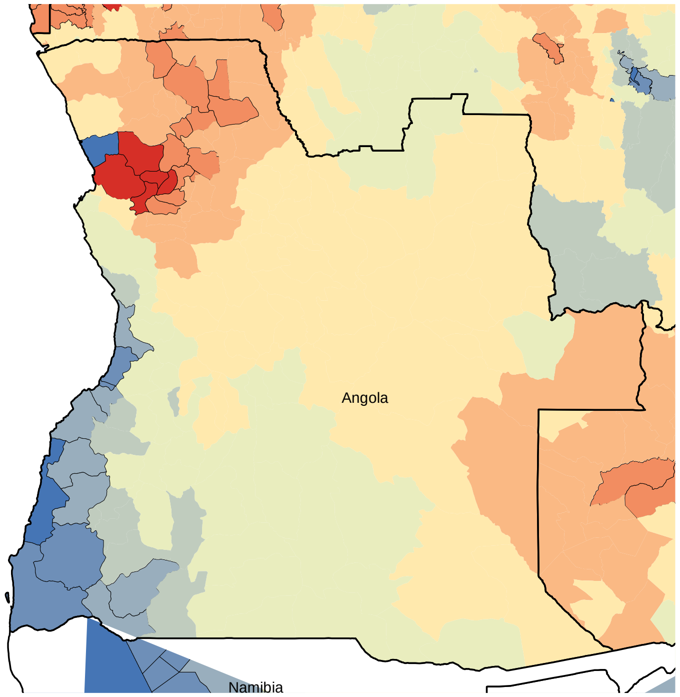
Angola
Namibia

BDI

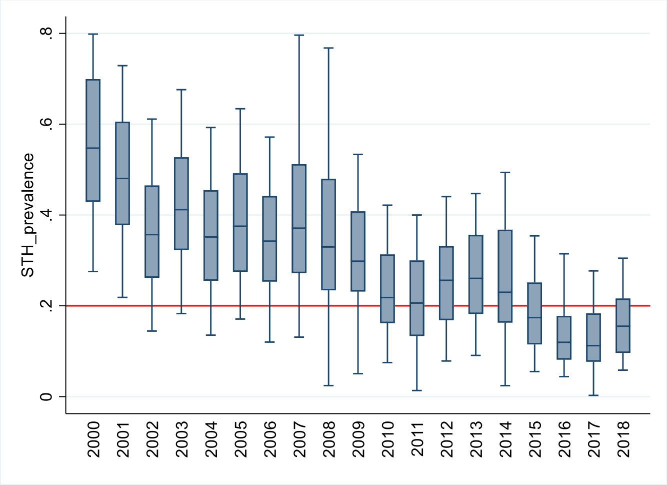
STH_prevalence
0
.2
.4
.6
.8
2000 2001 2002 2003 2004 2005 2006 2007 2008 2009 2010 2011 2012 2013 2014 2015 2016 2017 2018

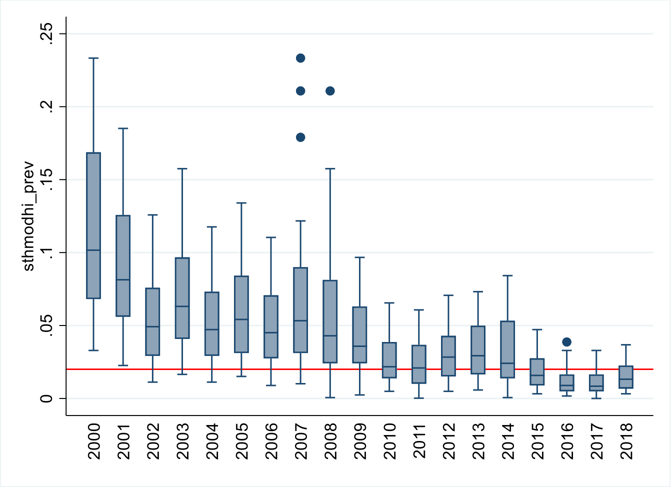
sthmodhi_prev
0
.05
.1
.15
.2
.25
2000 2001 2002 2003 2004 2005 2006 2007 2008 2009 2010 2011 2012 2013 2014 2015 2016 2017 2018

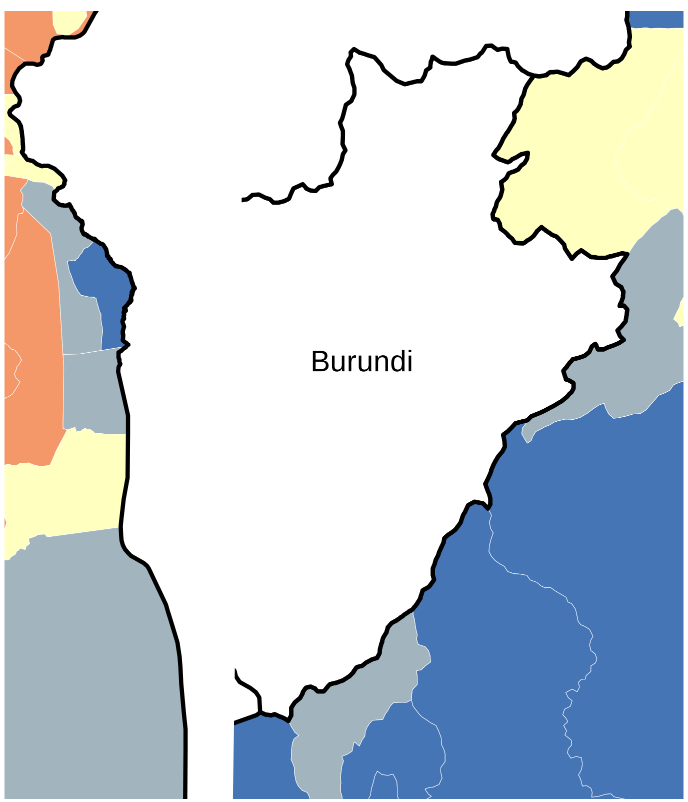
Burundi

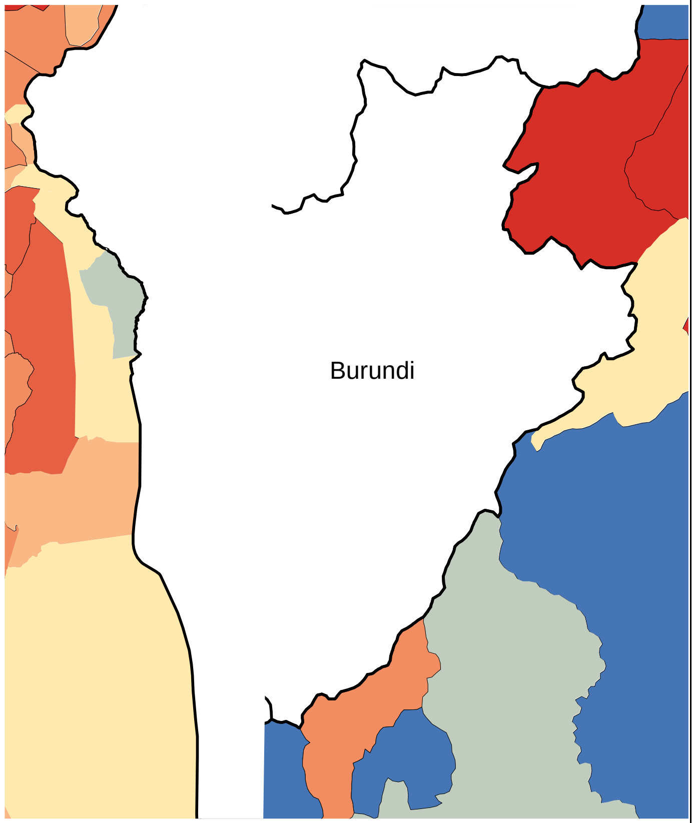
Burundi

**BEN**	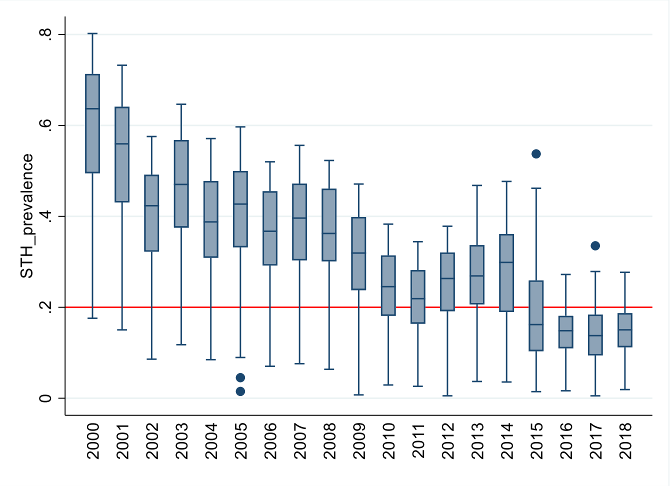 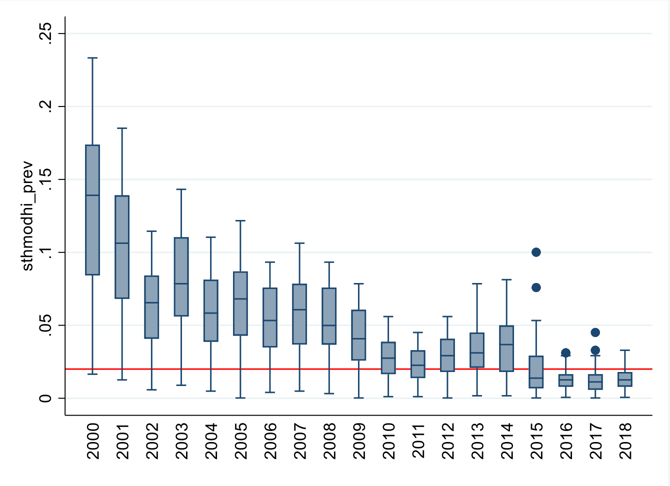	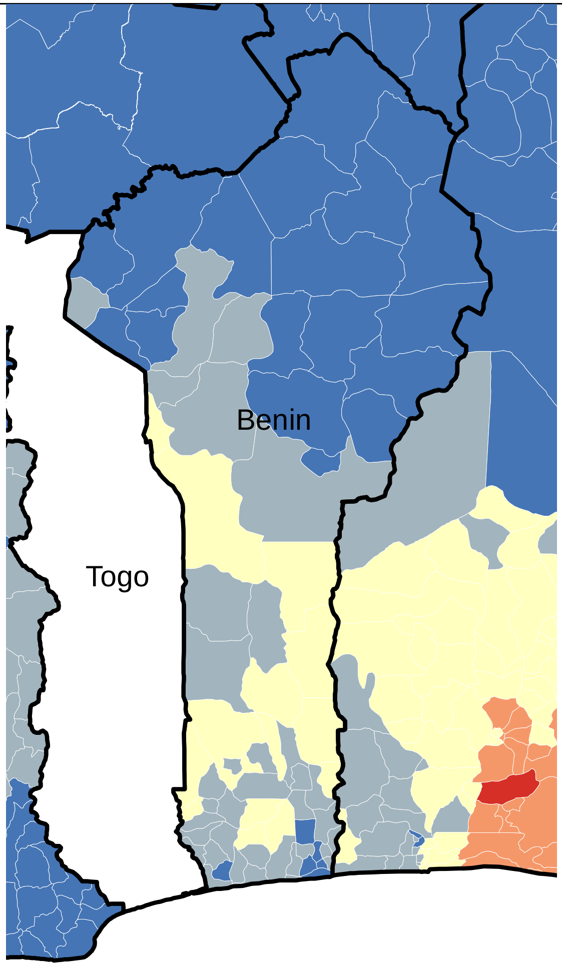	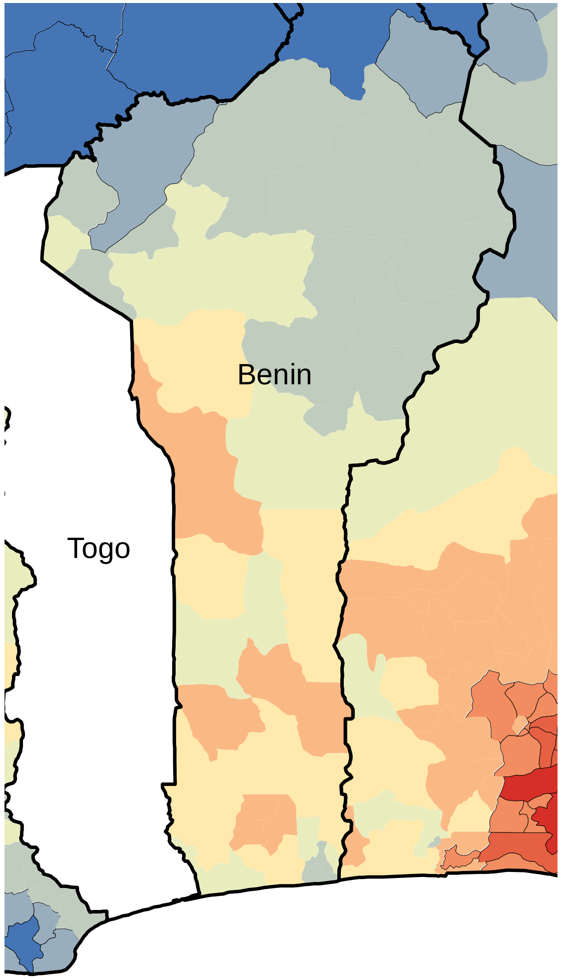

STH_prevalence
0
.2
.4
.6
.8
2000
2001
2002
2003
2004
2005
2006
2007
2008
2009
2010
2011
2012
2013
2014
2015
2016
2017
2018
sthmodhi_prev
0
.05
.1
.15
.2
.25
Togo
Benin

BFA

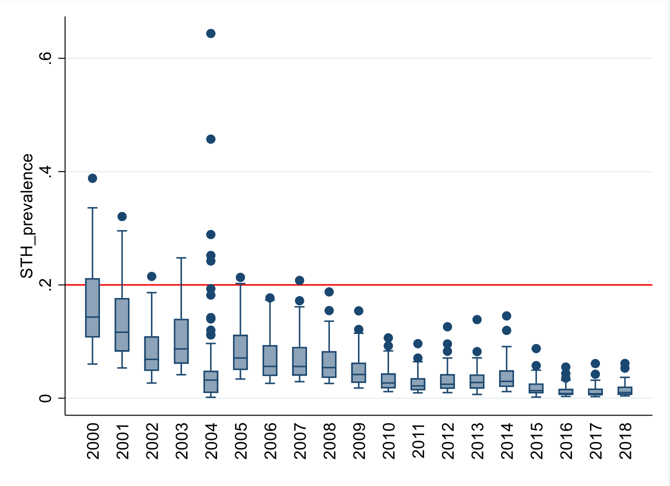
STH_prevalence
0
.2
.4
.6
2000 2001 2002 2003 2004 2005 2006 2007 2008 2009 2010 2011 2012 2013 2014 2015 2016 2017 2018

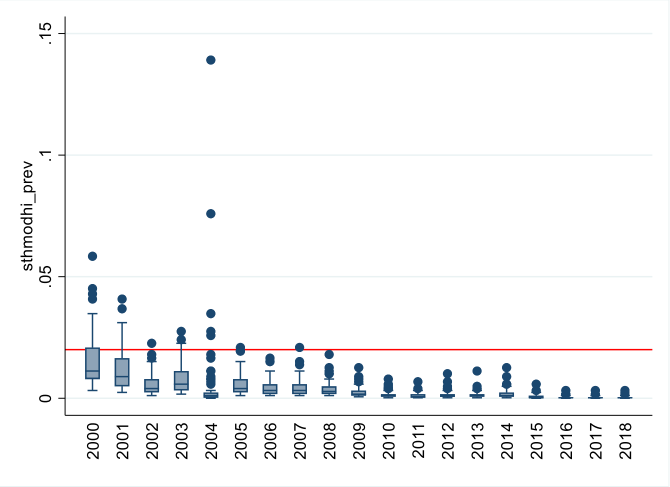
sthmodhi_prev
0
.05
.1
.15
2000 2001 2002 2003 2004 2005 2006 2007 2008 2009 2010 2011 2012 2013 2014 2015 2016 2017 2018

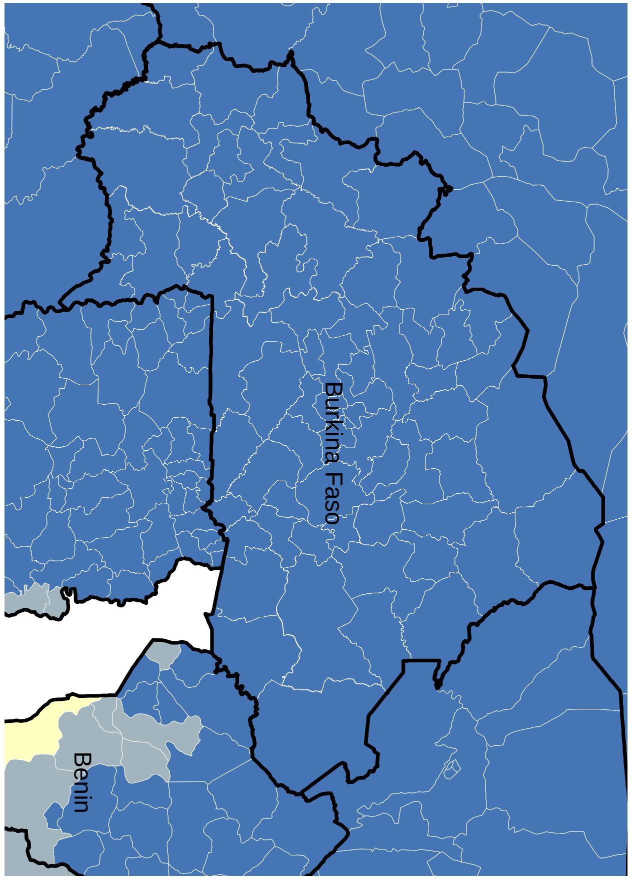
Burkina Faso
Benin

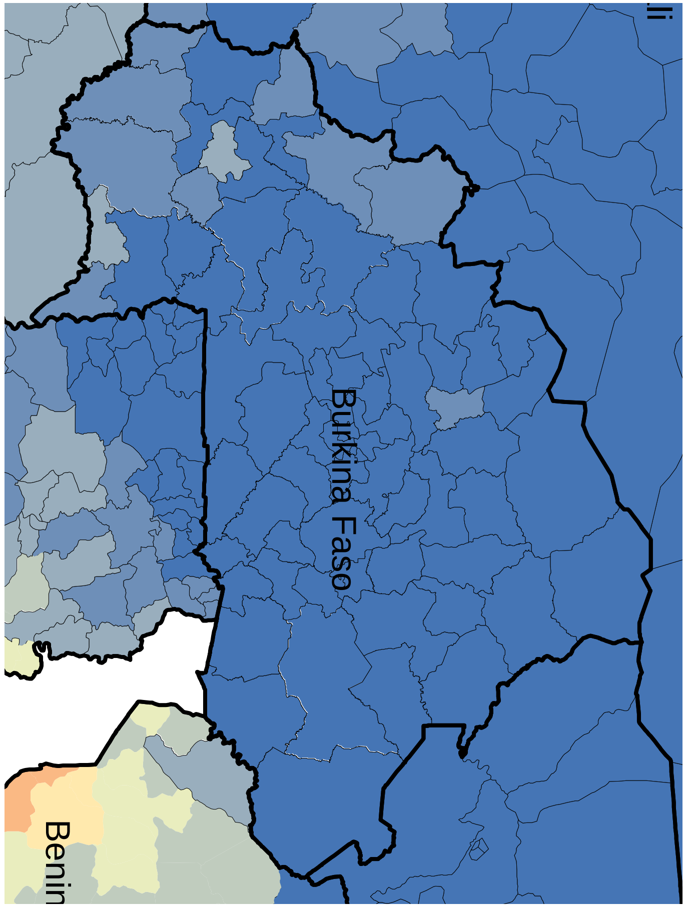
Burkina Faso
Benin

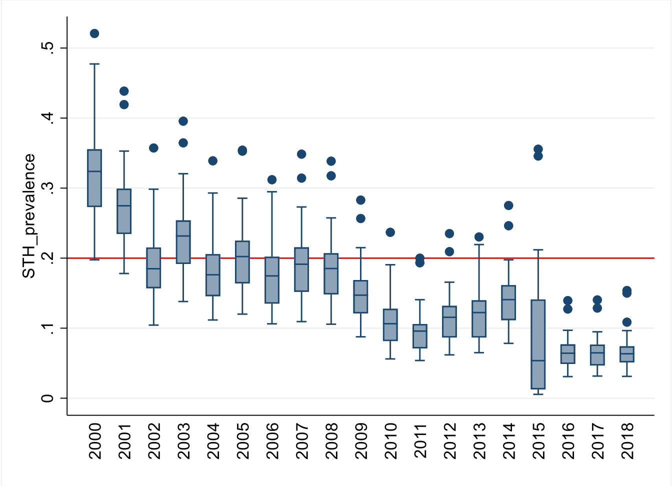
STH_prevalence
0
.1
.2
.3
.4
.5
2000
2001
2002
2003
2004
2005
2006
2007
2008
2009
2010
2011
2012
2013
2014
2015
2016
2017
2018

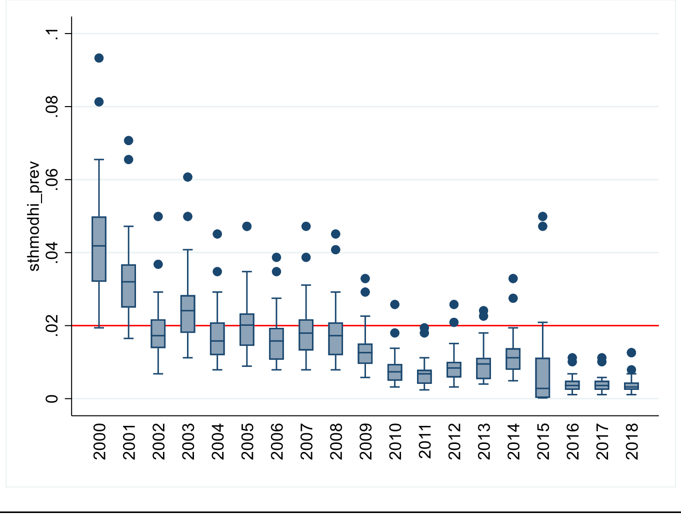
sthmodhi_prev
0
.02
.04
.06
.08
.1
2000
2001
2002
2003
2004
2005
2006
2007
2008
2009
2010
2011
2012
2013
2014
2015
2016
2017
2018

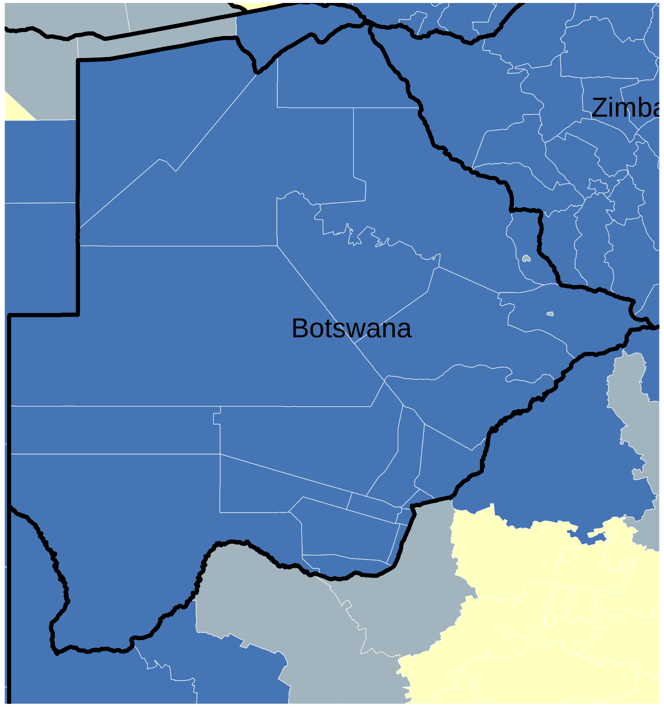
Botswana
Zimba

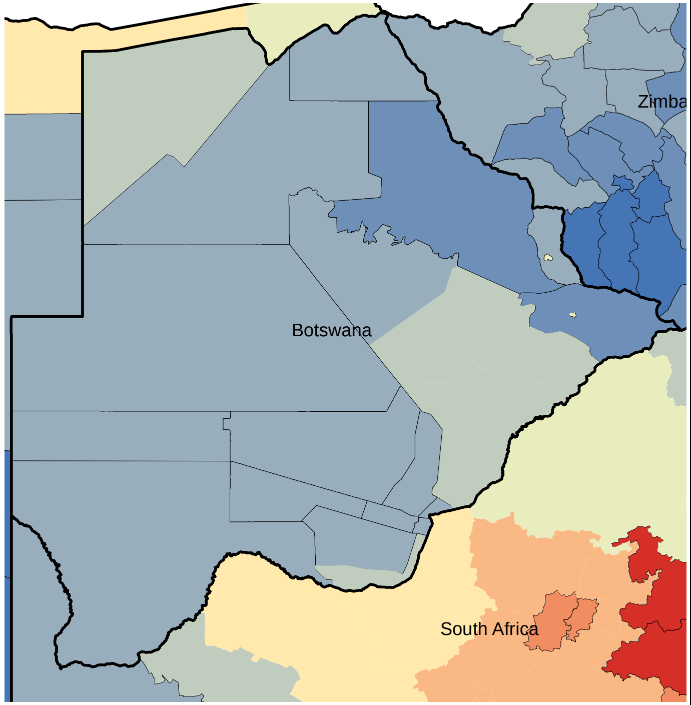
Botswana
Zimba
South Africa

BWA

**CAF**	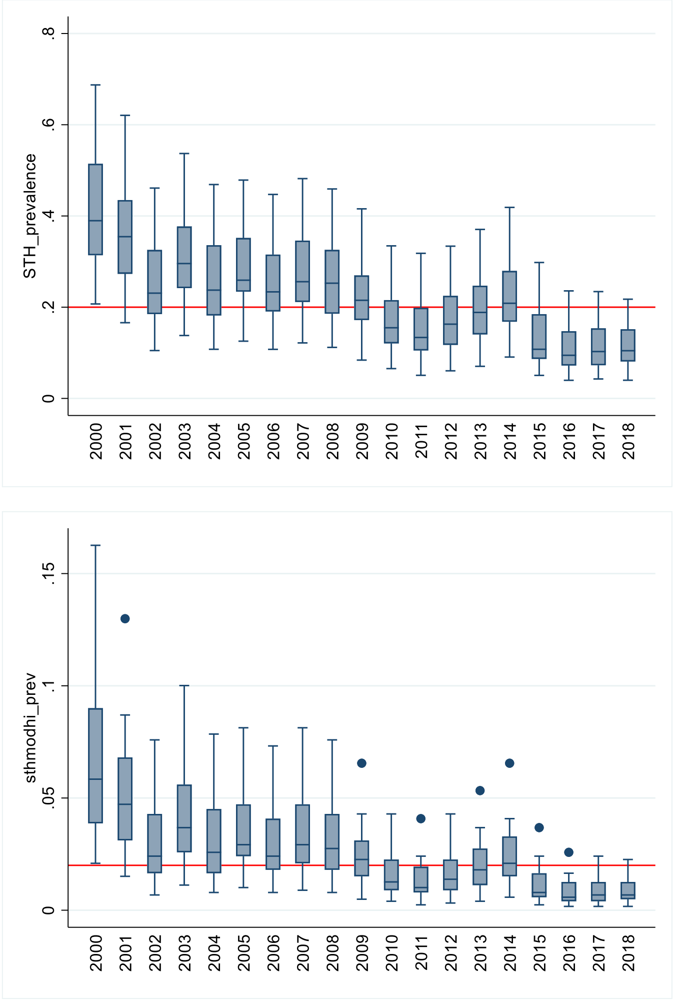STH_prevalence<br>sthmodhi_prev<br>2000 2001 2002 2003 2004 2005 2006 2007 2008 2009 2010 2011 2012 2013 2014 2015 2016 2017 2018	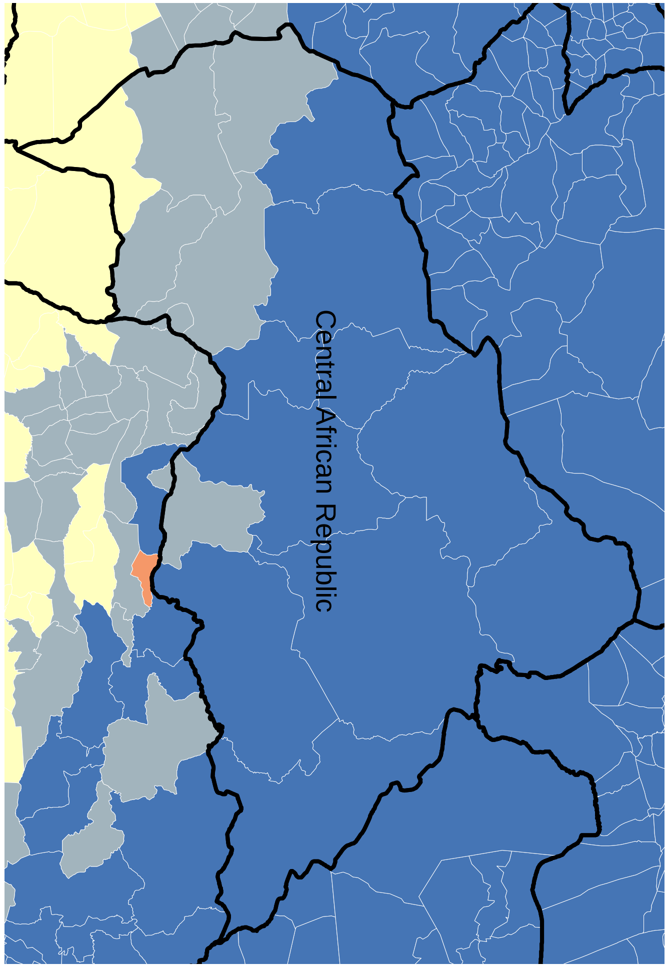Central African Republic	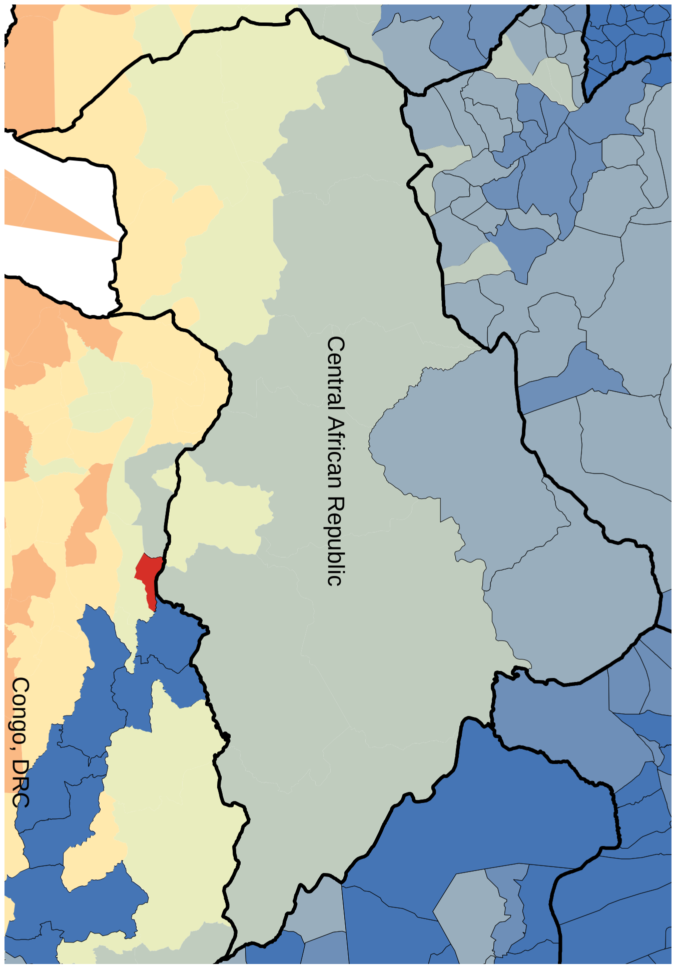Central African Republic<br>Congo, DRC

CIV

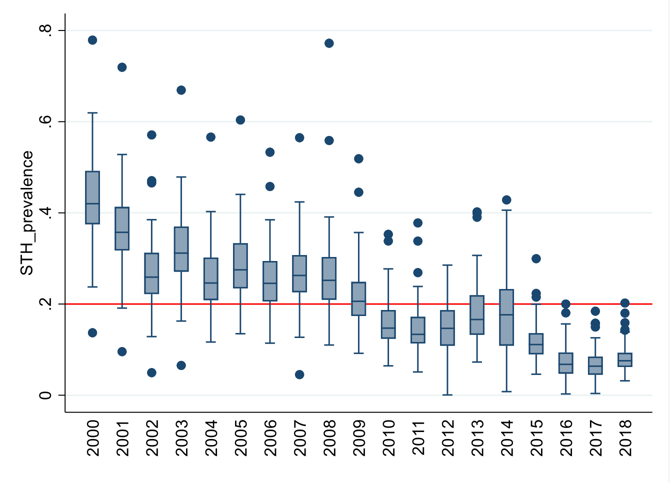
STH_prevalence
0
.2
.4
.6
.8
2000 2001 2002 2003 2004 2005 2006 2007 2008 2009 2010 2011 2012 2013 2014 2015 2016 2017 2018

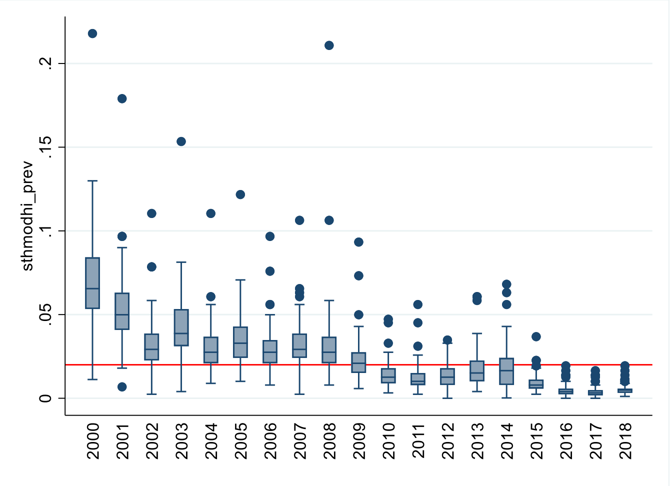
sthmodhi_prev
0
.05
.1
.15
.2
2000 2001 2002 2003 2004 2005 2006 2007 2008 2009 2010 2011 2012 2013 2014 2015 2016 2017 2018

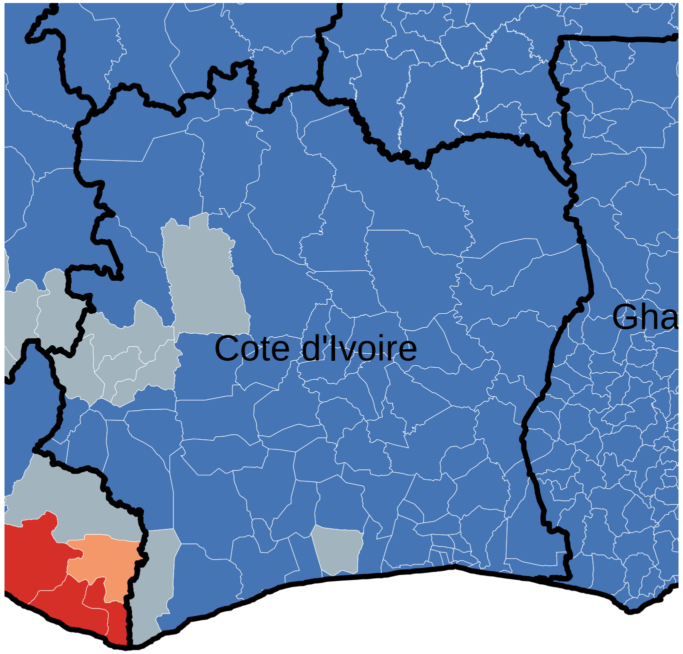
Cote d'Ivoire
Gha

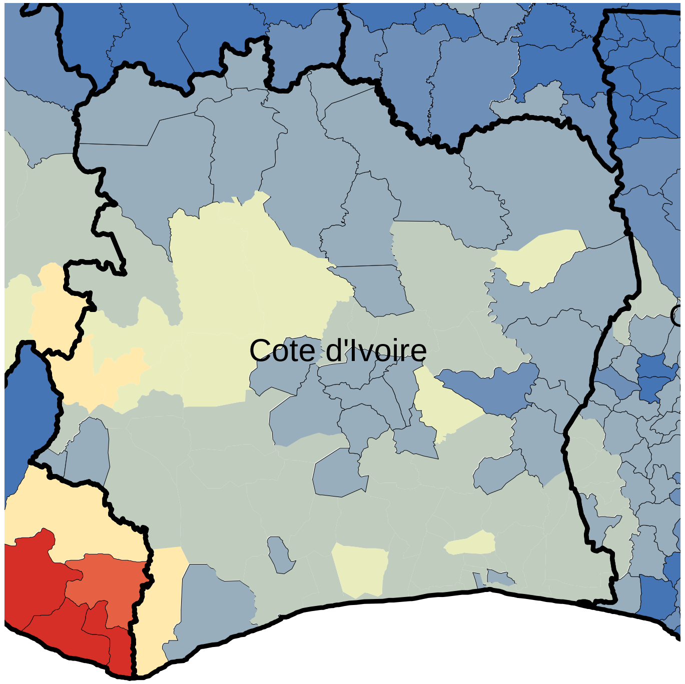
Cote d'Ivoire

CMR	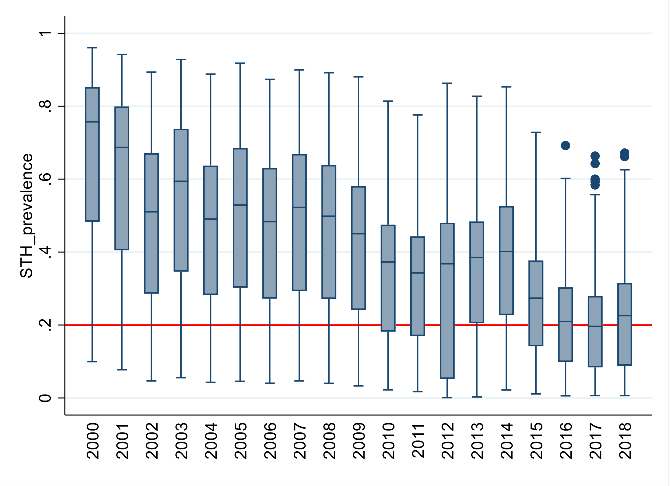<br>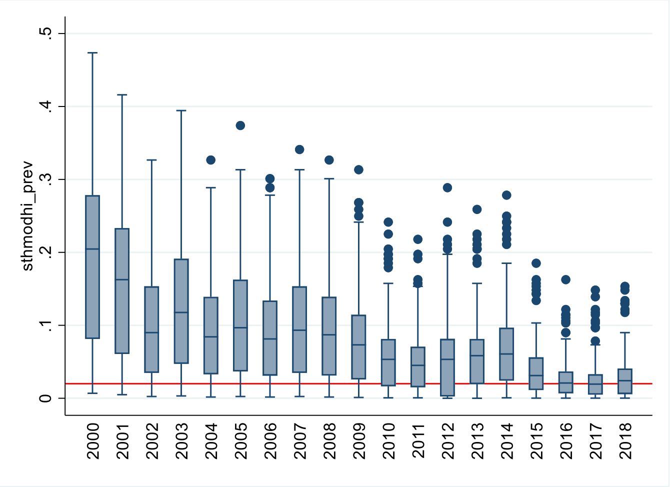	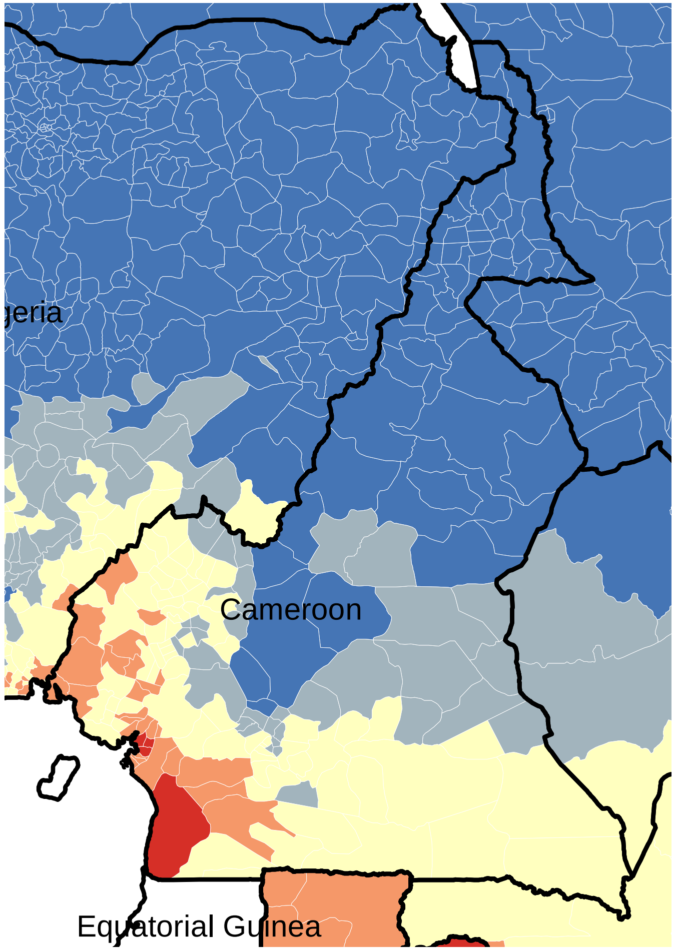	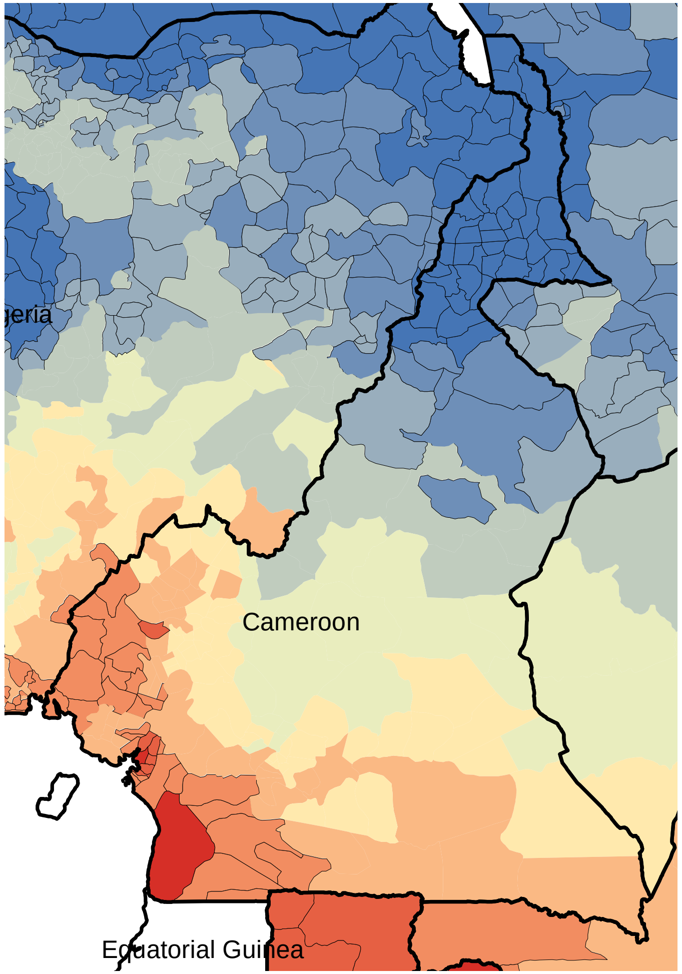

STH_prevalence
0 .2 .4 .6 .8 1
2000 2001 2002 2003 2004 2005 2006 2007 2008 2009 2010 2011 2012 2013 2014 2015 2016 2017 2018
sthmodhi_prev
0 .1 .2 .3 .4 .5
2000 2001 2002 2003 2004 2005 2006 2007 2008 2009 2010 2011 2012 2013 2014 2015 2016 2017 2018
Cameroon
Equatorial Guinea
Cameroon
Equatorial Guinea

**COD**	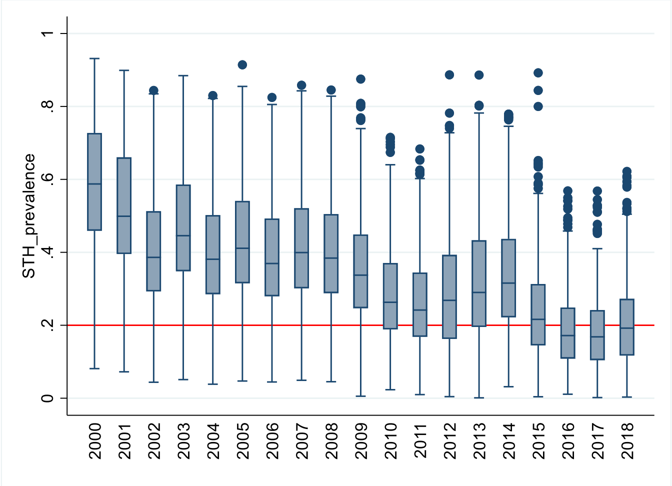 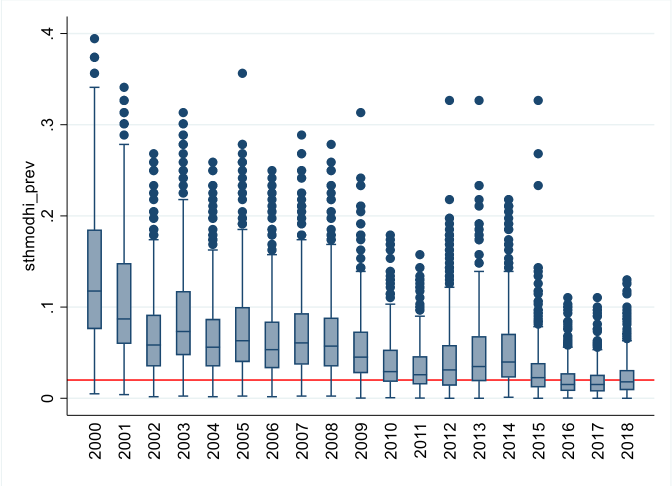	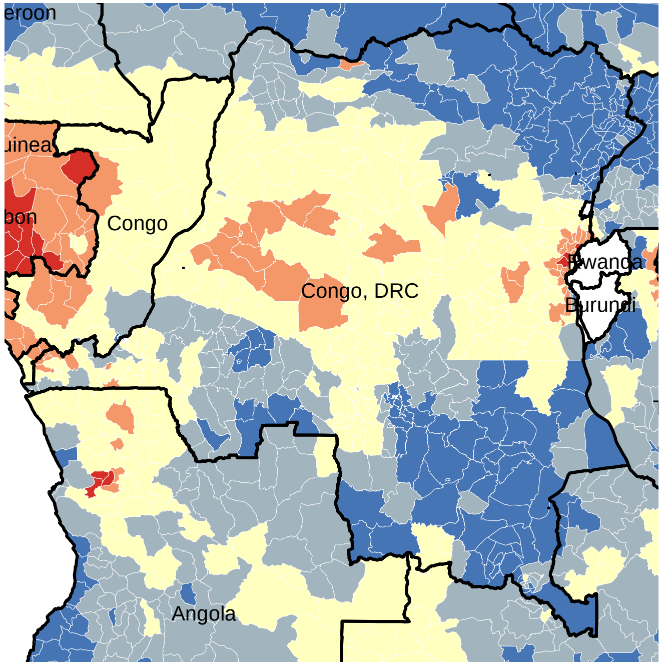	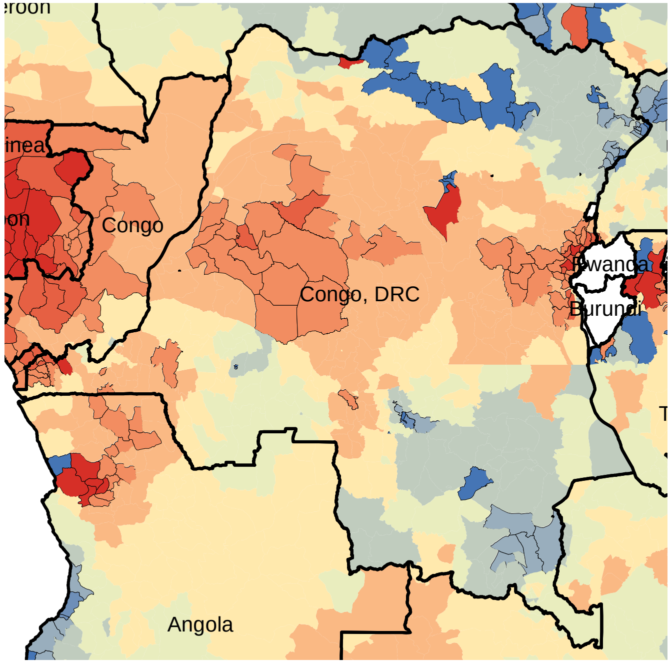

STH_prevalence
0
.2
.4
.6
.8
1
2000 2001 2002 2003 2004 2005 2006 2007 2008 2009 2010 2011 2012 2013 2014 2015 2016 2017 2018

sthmodhi_prev
0
.1
.2
.3
.4
2000 2001 2002 2003 2004 2005 2006 2007 2008 2009 2010 2011 2012 2013 2014 2015 2016 2017 2018

Congo
Congo, DRC
Rwanda
Burundi
Angola

Congo
Congo, DRC
Rwanda
Burundi
Angola

COG	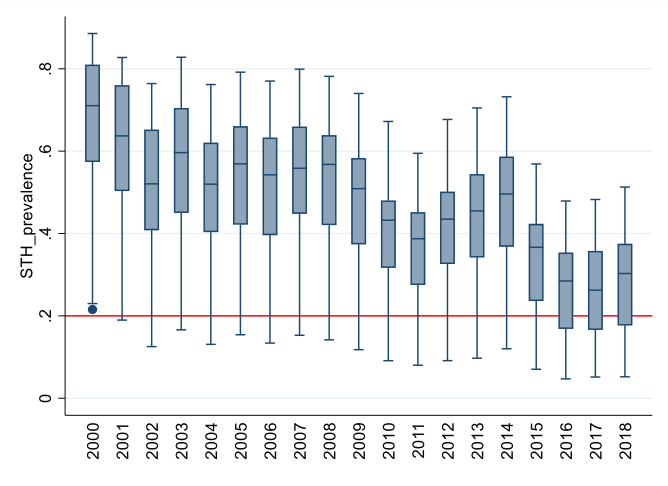<br><br>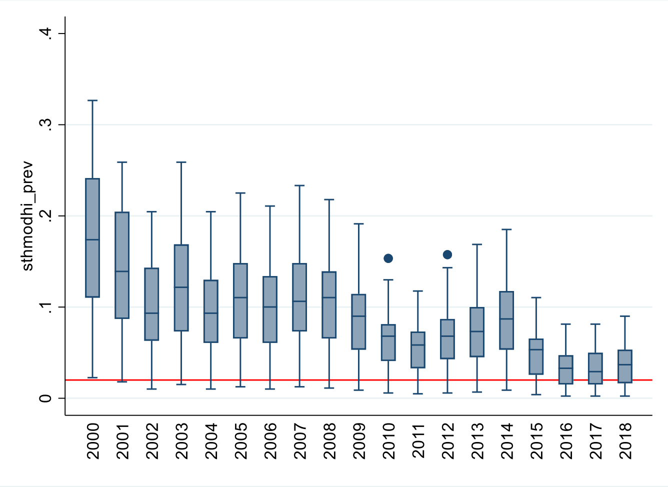 	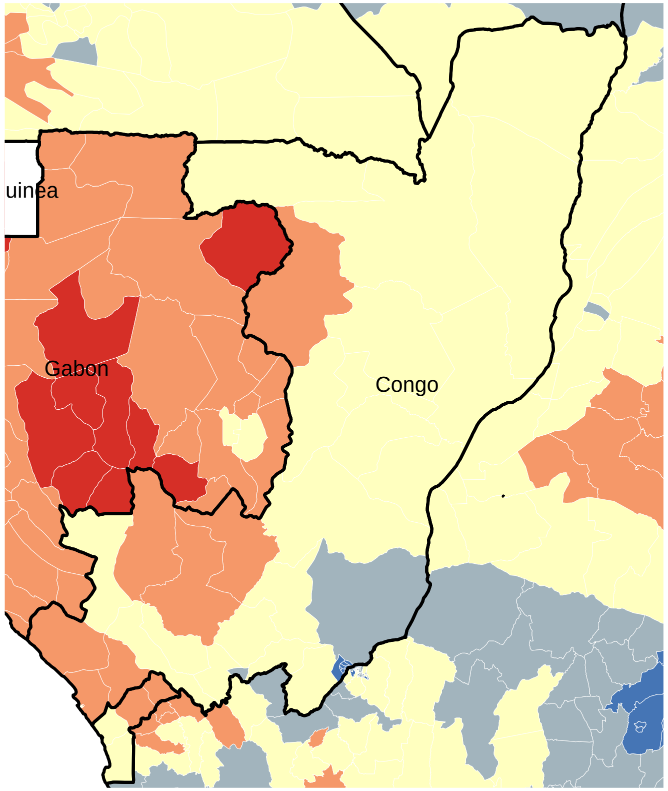 	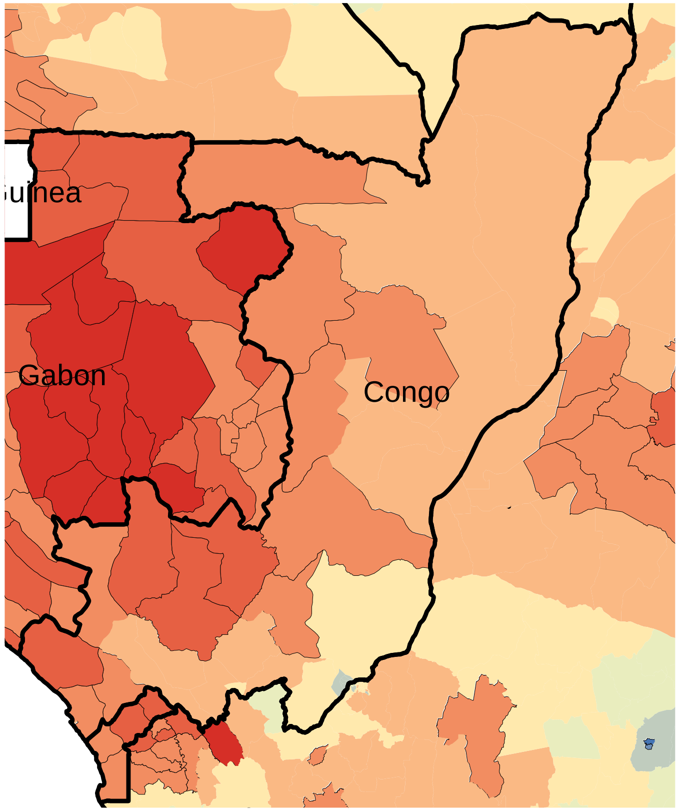

DJI

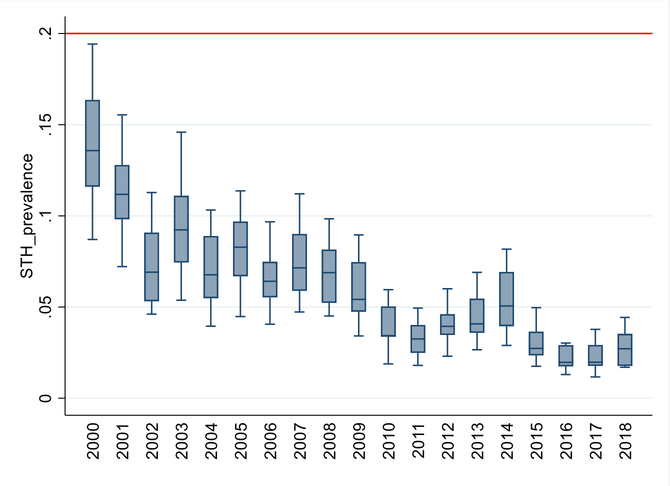
STH_prevalence
0
.05
.1
.15
.2
2000 2001 2002 2003 2004 2005 2006 2007 2008 2009 2010 2011 2012 2013 2014 2015 2016 2017 2018

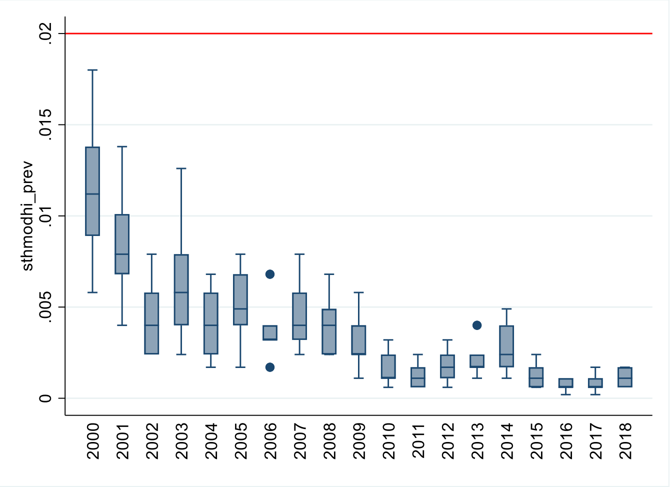
sthmodhi_prev
0
.005
.01
.015
.02
2000 2001 2002 2003 2004 2005 2006 2007 2008 2009 2010 2011 2012 2013 2014 2015 2016 2017 2018

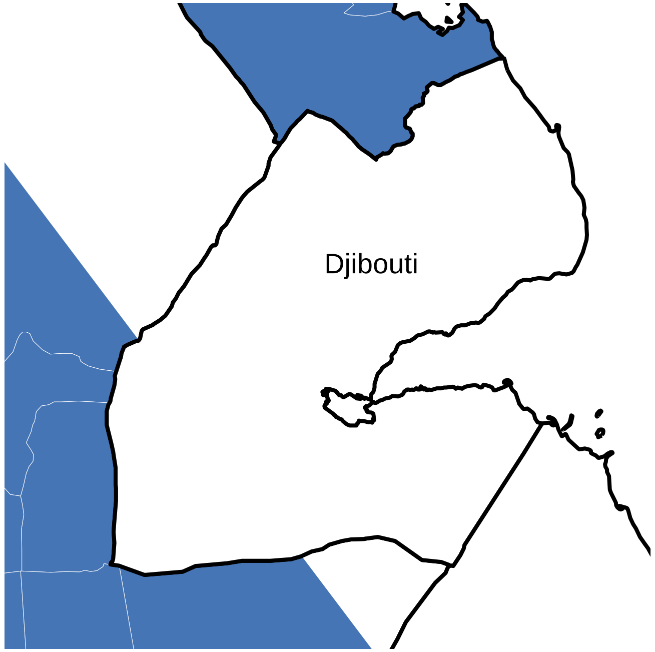
Djibouti

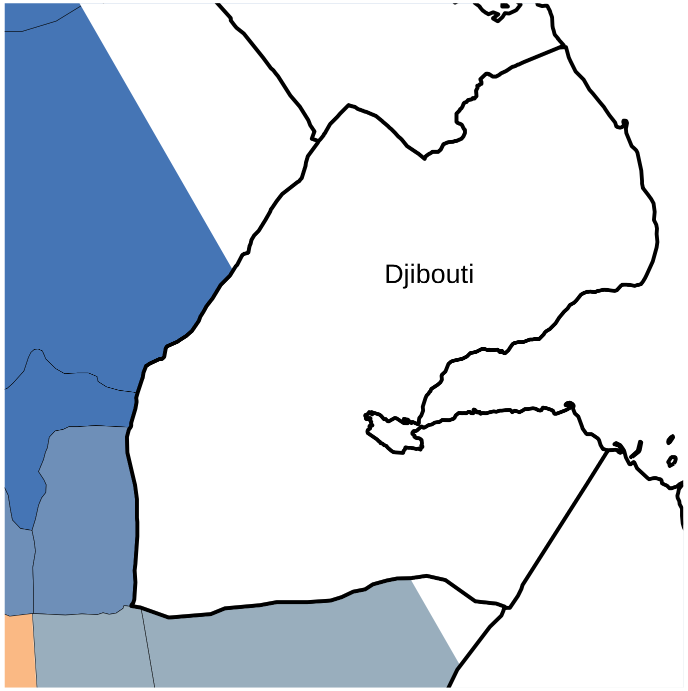
Djibouti

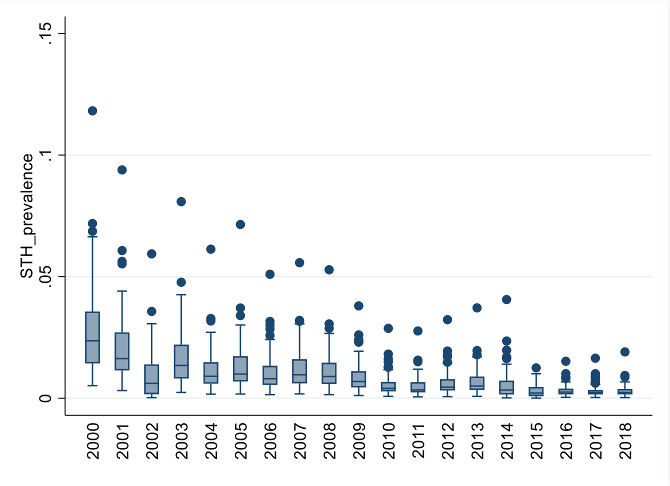
STH_prevalence
0
.05
.1
.15
2000
2001
2002
2003
2004
2005
2006
2007
2008
2009
2010
2011
2012
2013
2014
2015
2016
2017
2018

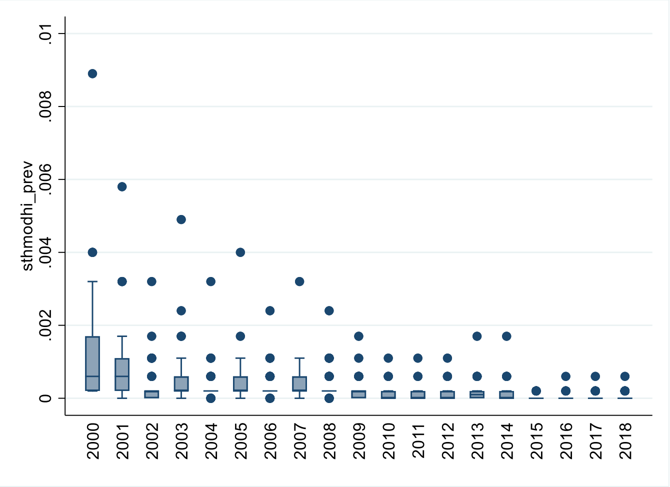
sthmodhi_prev
0
.002
.004
.006
.008
.01
2000
2001
2002
2003
2004
2005
2006
2007
2008
2009
2010
2011
2012
2013
2014
2015
2016
2017
2018

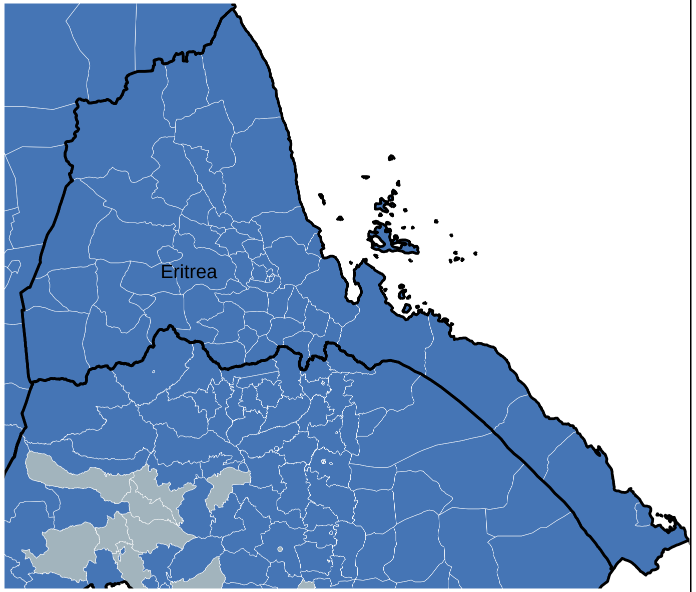
Eritrea

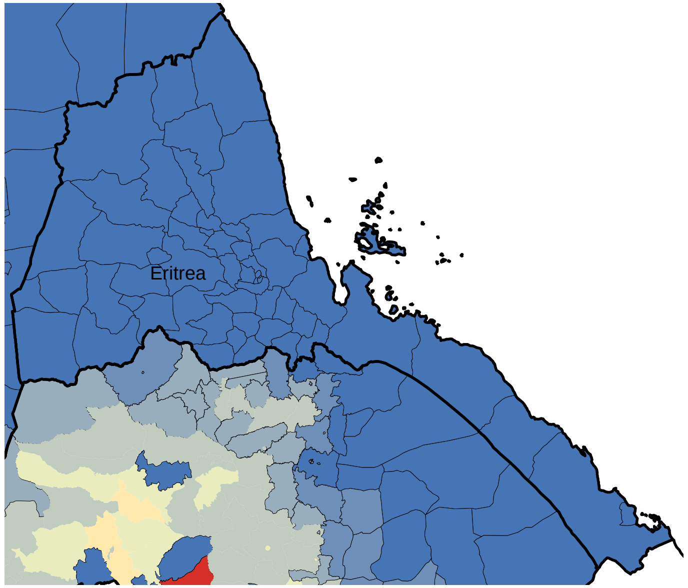
Eritrea

ERI

ETH

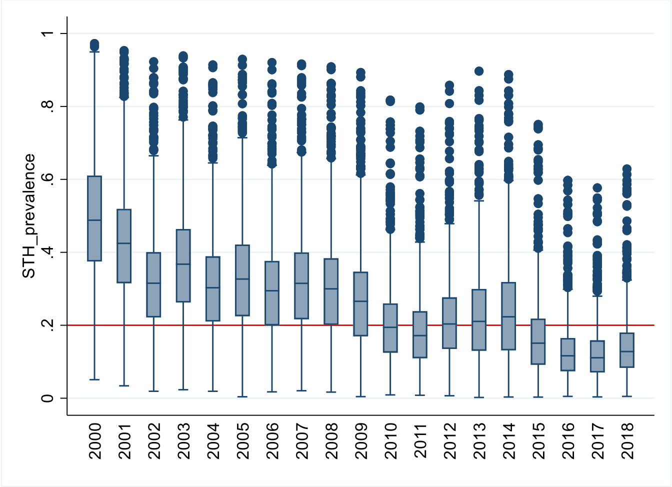
STH_prevalence
0
.2
.4
.6
.8
1
2000 2001 2002 2003 2004 2005 2006 2007 2008 2009 2010 2011 2012 2013 2014 2015 2016 2017 2018

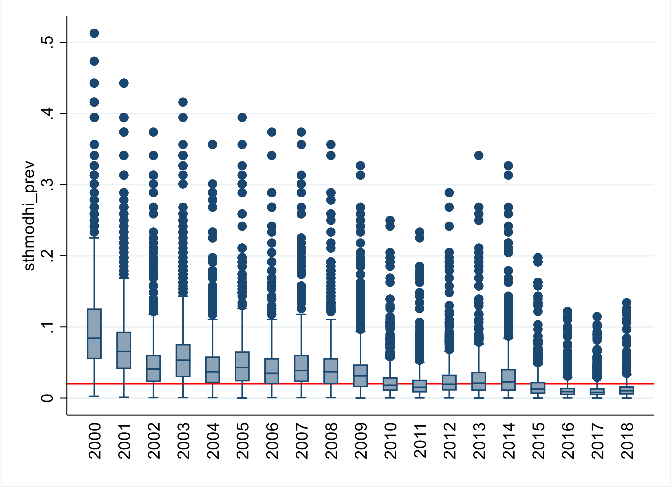
sthmodhi_prev
0
.1
.2
.3
.4
.5
2000 2001 2002 2003 2004 2005 2006 2007 2008 2009 2010 2011 2012 2013 2014 2015 2016 2017 2018

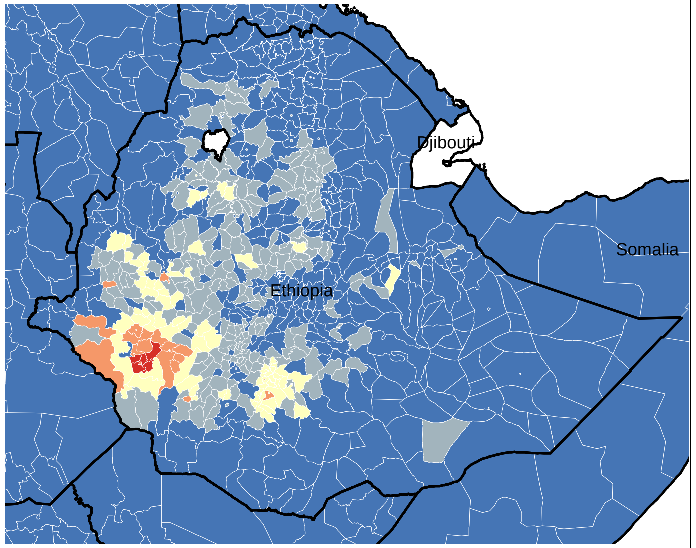
Djibouti
Somalia
Ethiopia

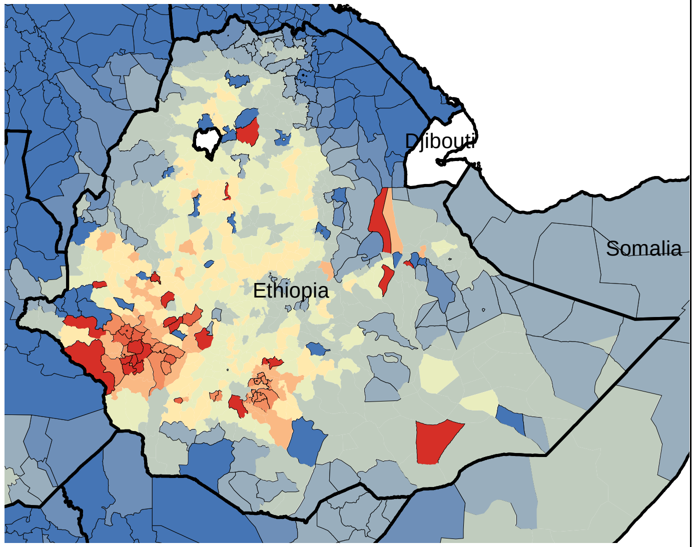
Djibouti
Somalia
Ethiopia

**GAB**	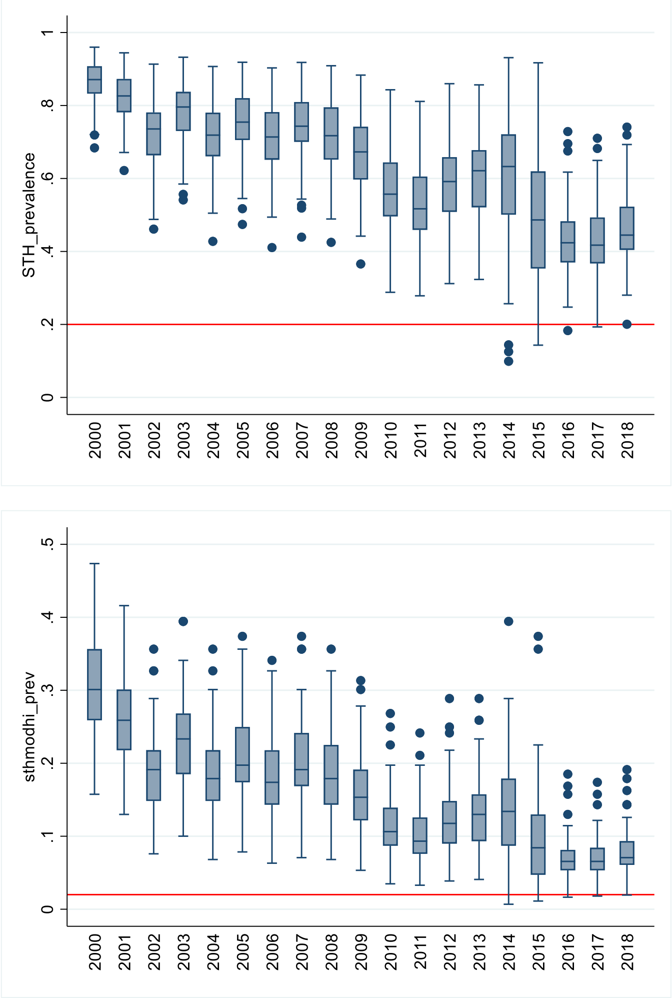STH_prevalence<br>0 .2 .4 .6 .8 1<br>2000 2001 2002 2003 2004 2005 2006 2007 2008 2009 2010 2011 2012 2013 2014 2015 2016 2017 2018<br>sthmodhi_prev<br>0 .1 .2 .3 .4 .5<br>2000 2001 2002 2003 2004 2005 2006 2007 2008 2009 2010 2011 2012 2013 2014 2015 2016 2017 2018	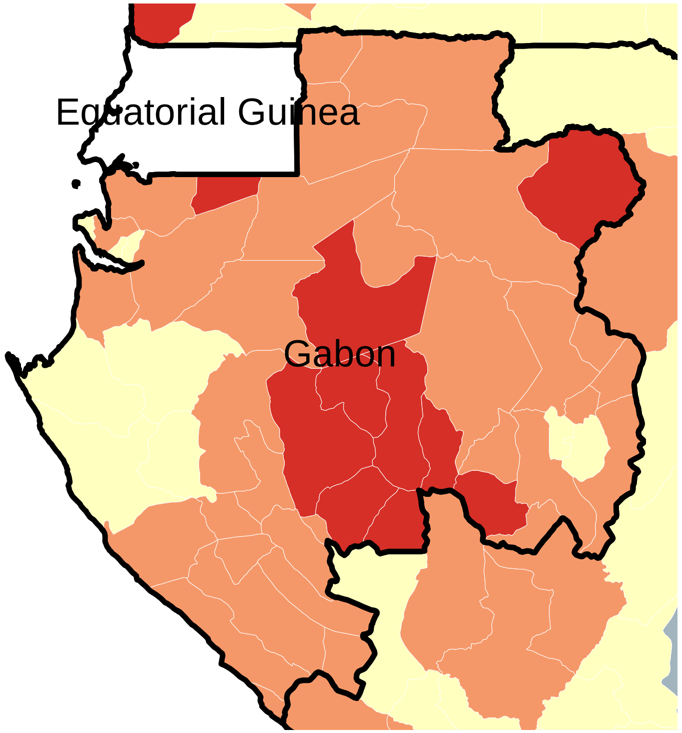Equatorial Guinea<br>Gabon	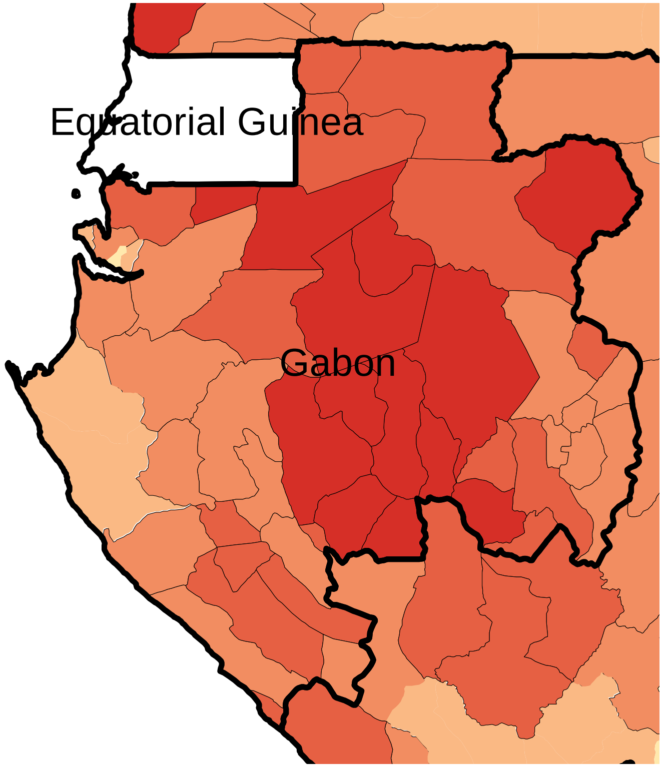Equatorial Guinea<br>Gabon

GHA

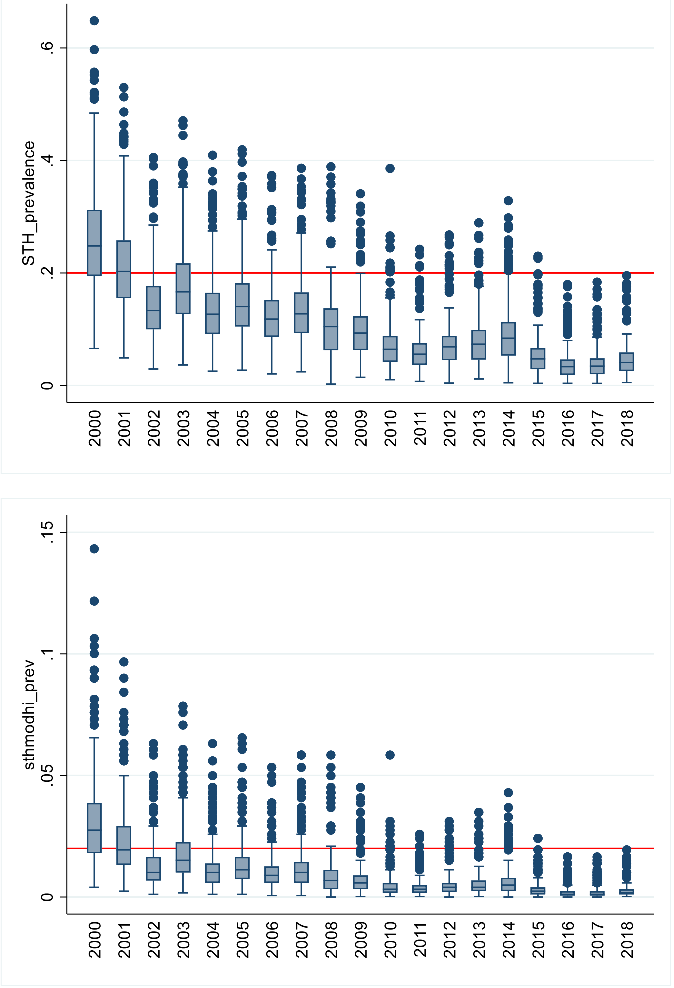
STH_prevalence
0
.2
.4
.6
sthmodhi_prev
0
.05
.1
.15
2000
2001
2002
2003
2004
2005
2006
2007
2008
2009
2010
2011
2012
2013
2014
2015
2016
2017
2018

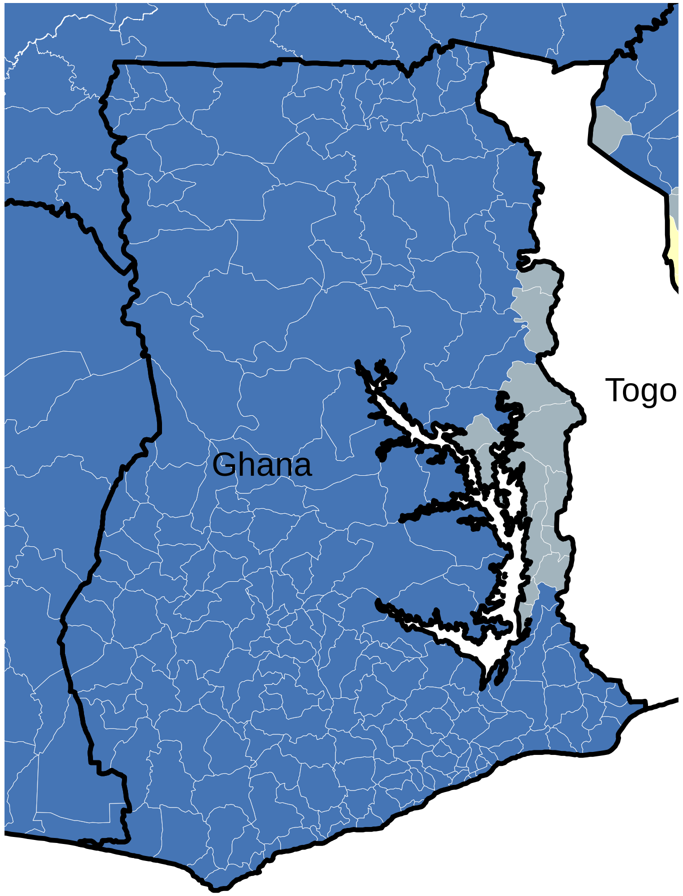
Ghana
Togo

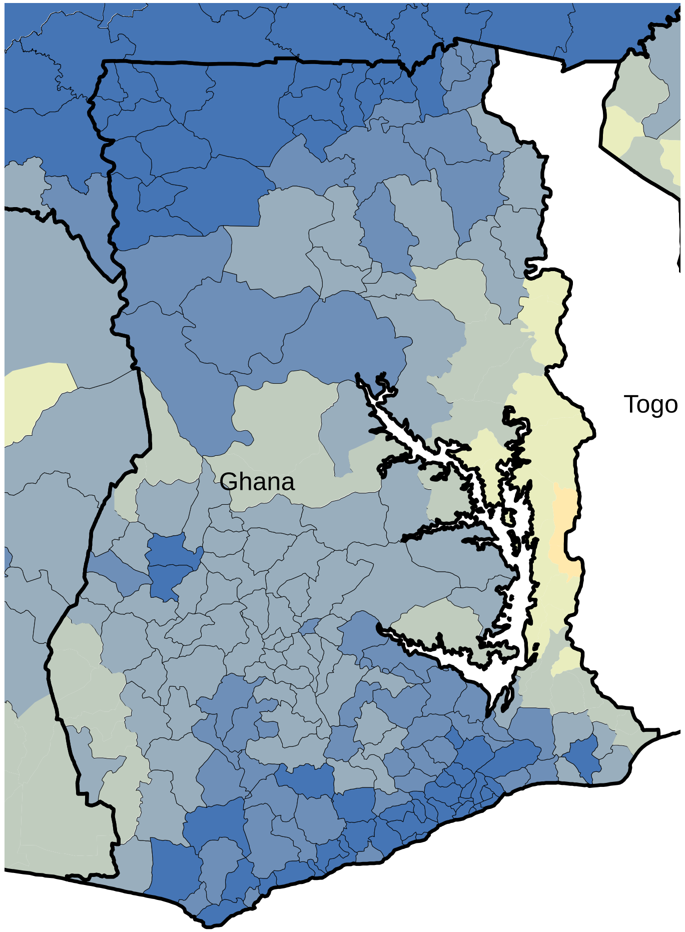
Ghana
Togo

GIN

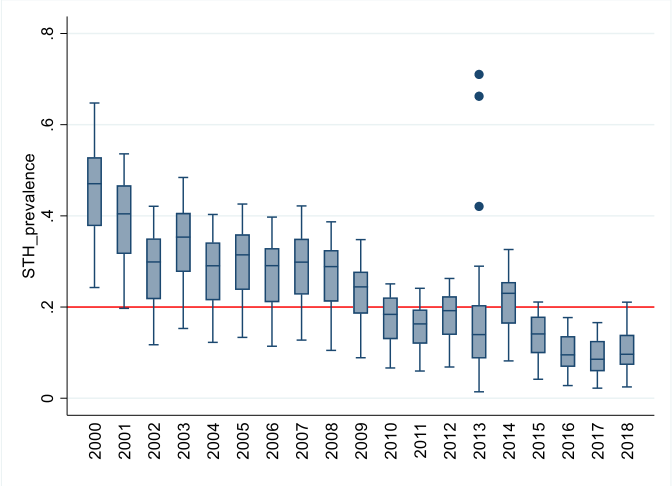
STH_prevalence
0
.2
.4
.6
.8
2000 2001 2002 2003 2004 2005 2006 2007 2008 2009 2010 2011 2012 2013 2014 2015 2016 2017 2018

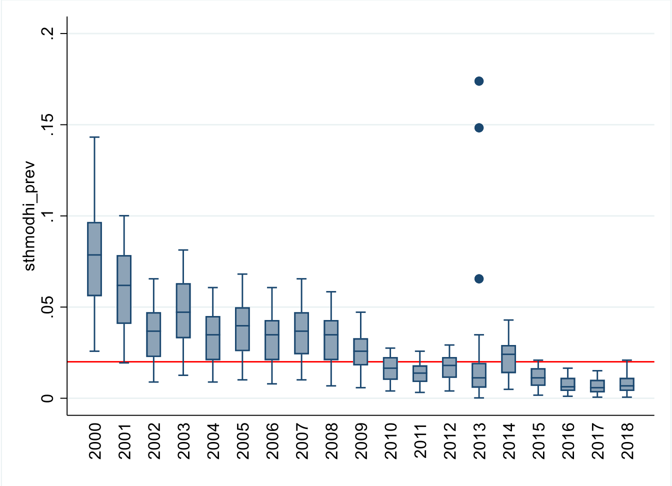
sthmodhi_prev
0
.05
.1
.15
.2
2000 2001 2002 2003 2004 2005 2006 2007 2008 2009 2010 2011 2012 2013 2014 2015 2016 2017 2018

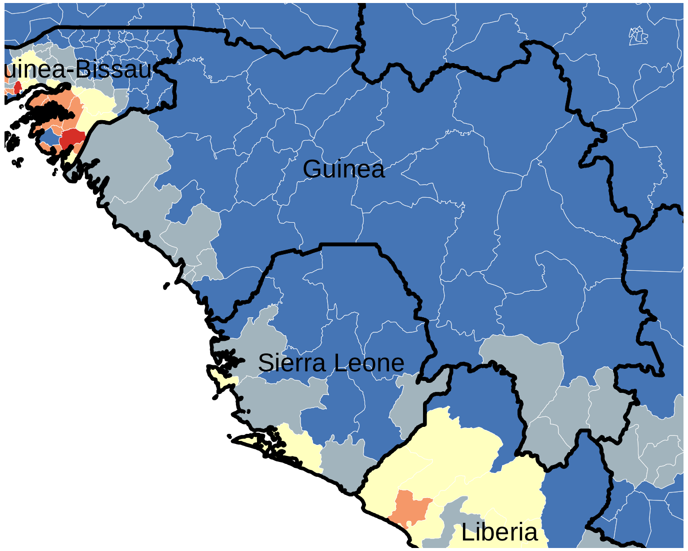
uinea-Bissau
Guinea
Sierra Leone
Liberia

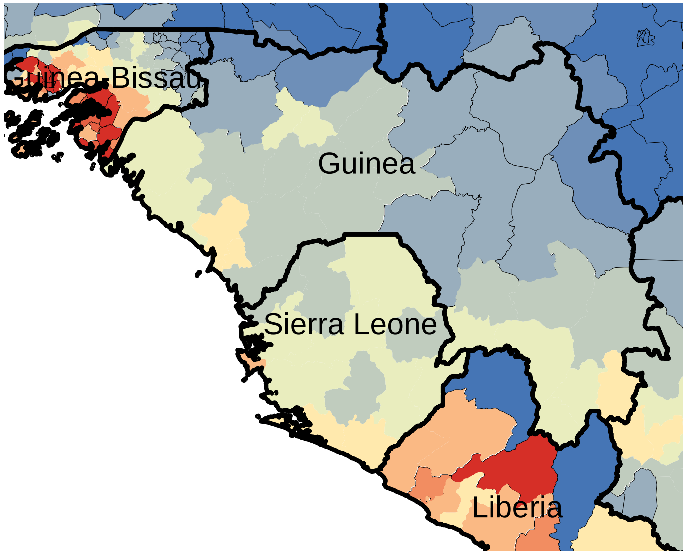
Guinea-Bissau
Guinea
Sierra Leone
Liberia

GMB

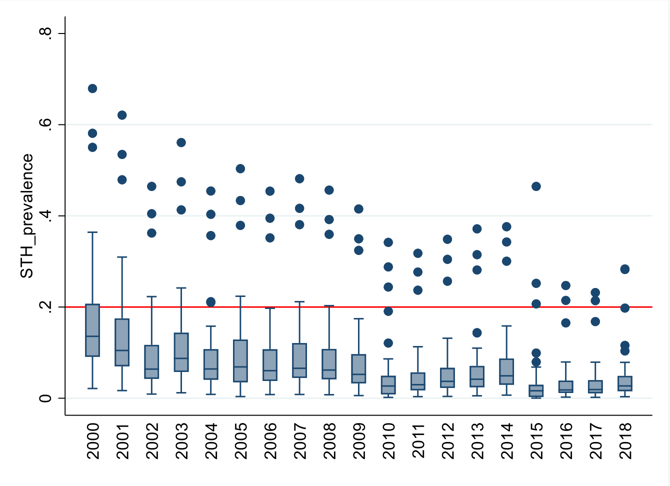
STH_prevalence
0
.2
.4
.6
.8
2000 2001 2002 2003 2004 2005 2006 2007 2008 2009 2010 2011 2012 2013 2014 2015 2016 2017 2018

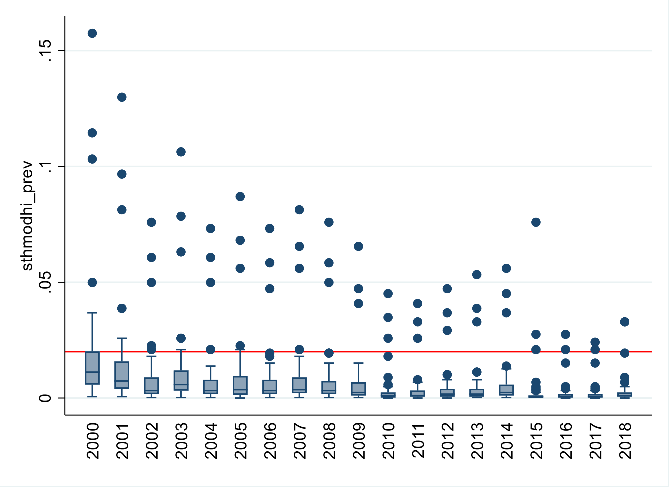
sthmodhi_prev
0
.05
.1
.15
2000 2001 2002 2003 2004 2005 2006 2007 2008 2009 2010 2011 2012 2013 2014 2015 2016 2017 2018

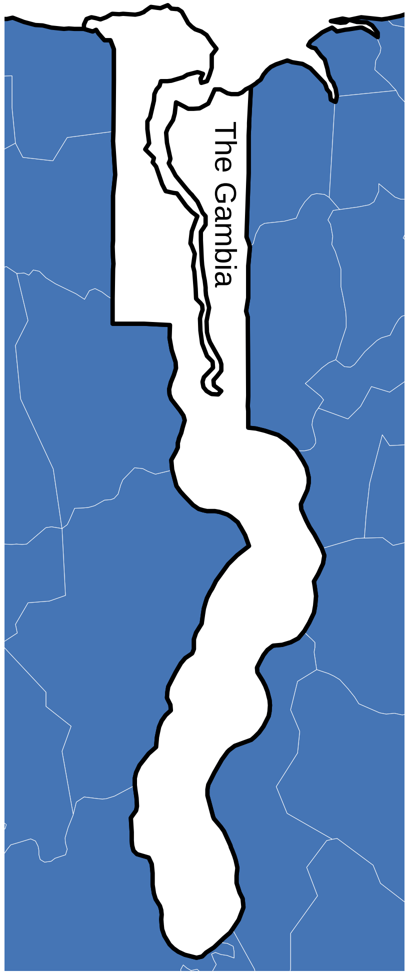
The Gambia

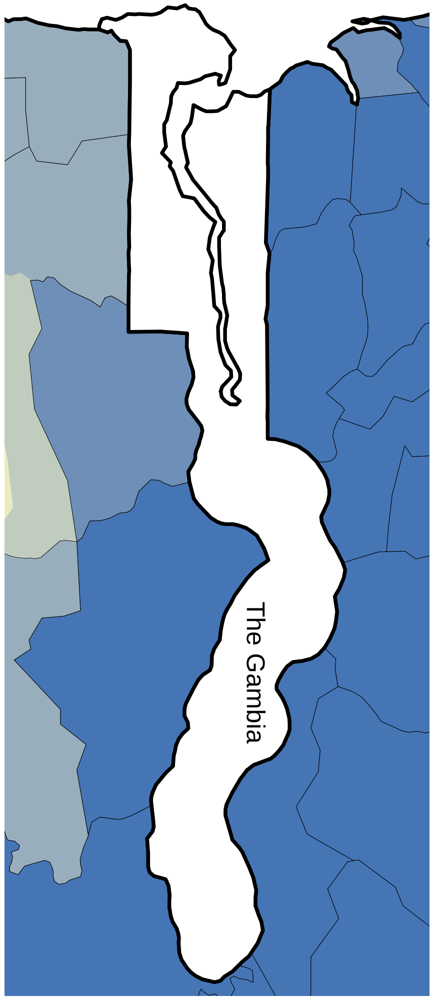
The Gambia

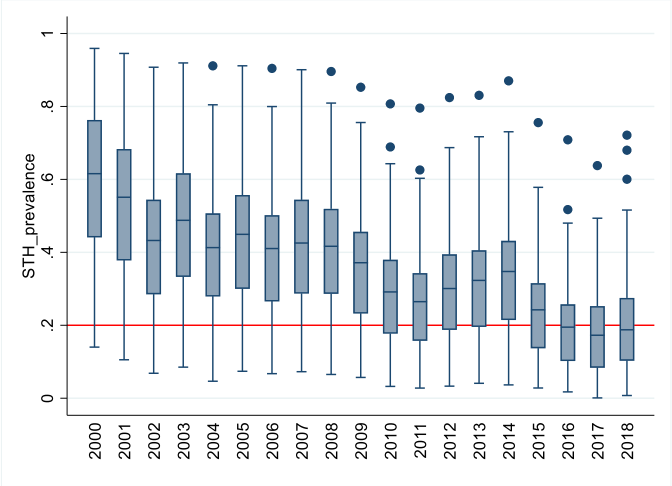
STH_prevalence
0
.2
.4
.6
.8
1
2000
2001
2002
2003
2004
2005
2006
2007
2008
2009
2010
2011
2012
2013
2014
2015
2016
2017
2018

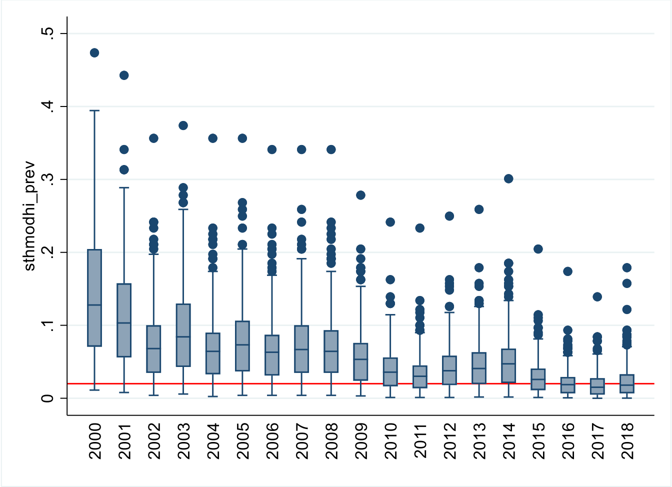
sthmodhi_prev
0
.1
.2
.3
.4
.5
2000
2001
2002
2003
2004
2005
2006
2007
2008
2009
2010
2011
2012
2013
2014
2015
2016
2017
2018

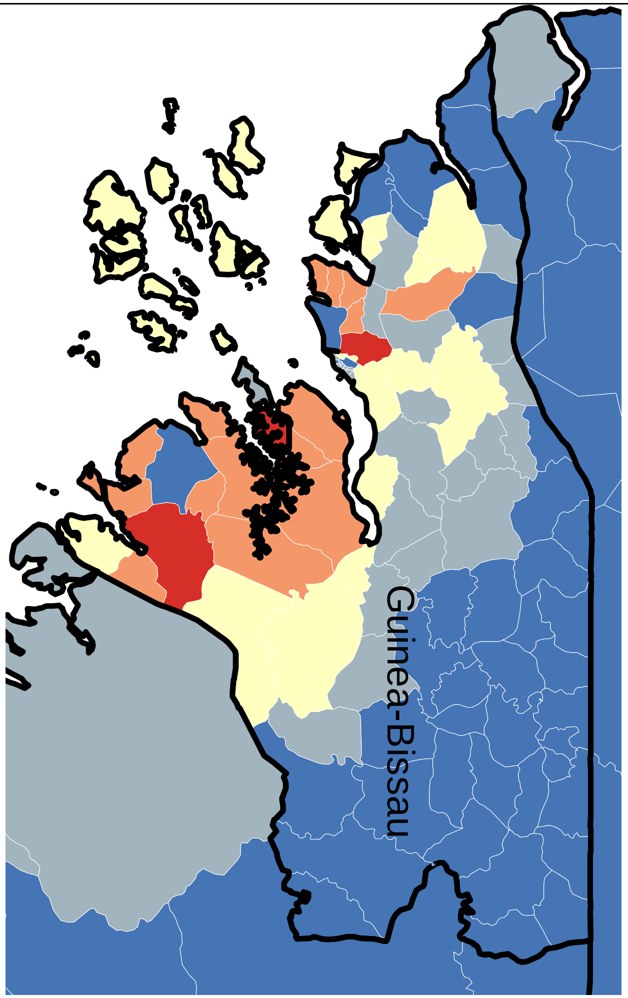
Guinea-Bissau

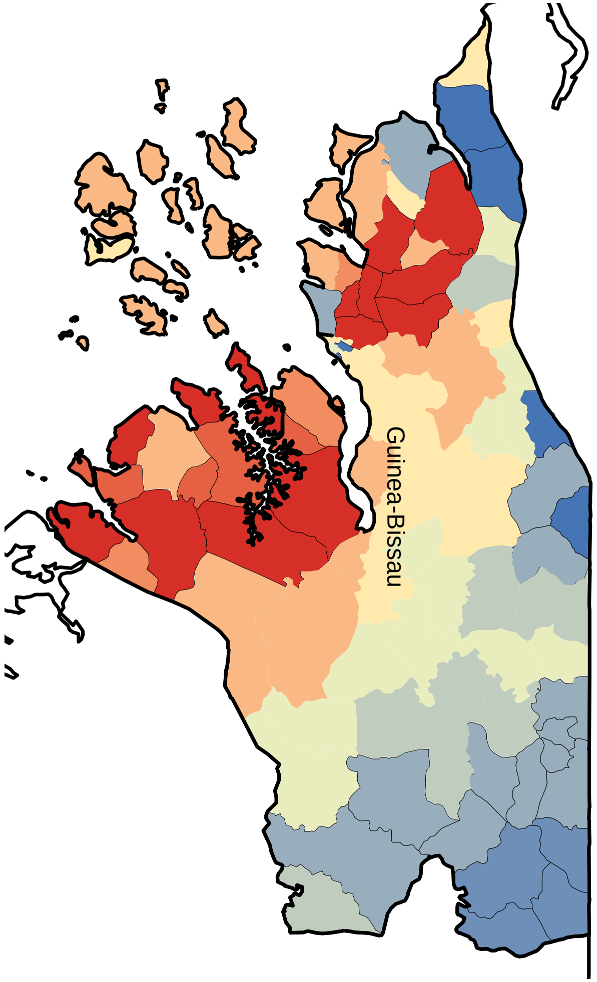
Guinea-Bissau

GNB

GNQ	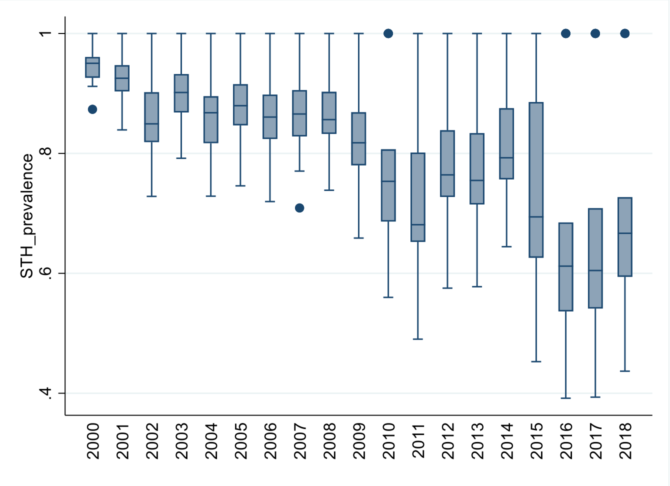STH_prevalence<br>.4<br>.6<br>.8<br>1<br>2000 2001 2002 2003 2004 2005 2006 2007 2008 2009 2010 2011 2012 2013 2014 2015 2016 2017 2018<br>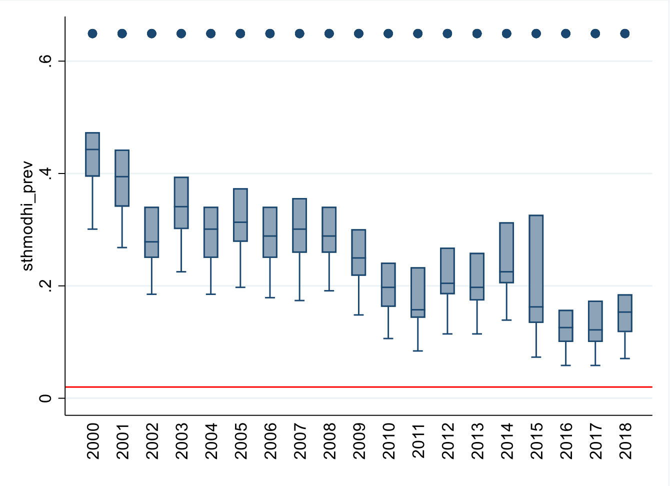sthmodhi_prev<br>0<br>.2<br>.4<br>.6<br>2000 2001 2002 2003 2004 2005 2006 2007 2008 2009 2010 2011 2012 2013 2014 2015 2016 2017 2018	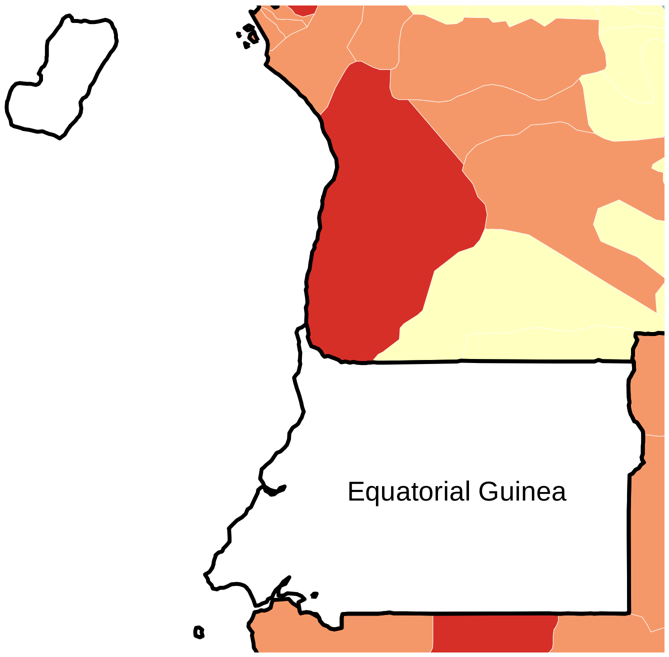Equatorial Guinea	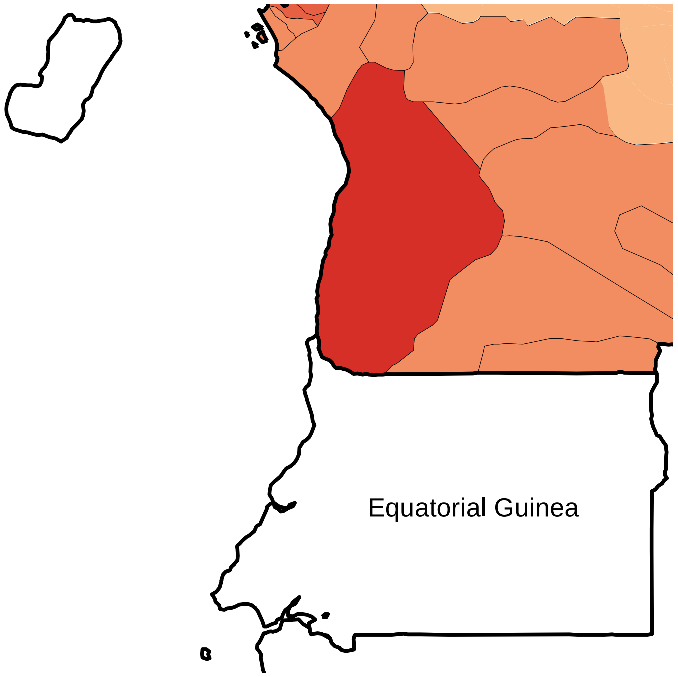Equatorial Guinea

KEN

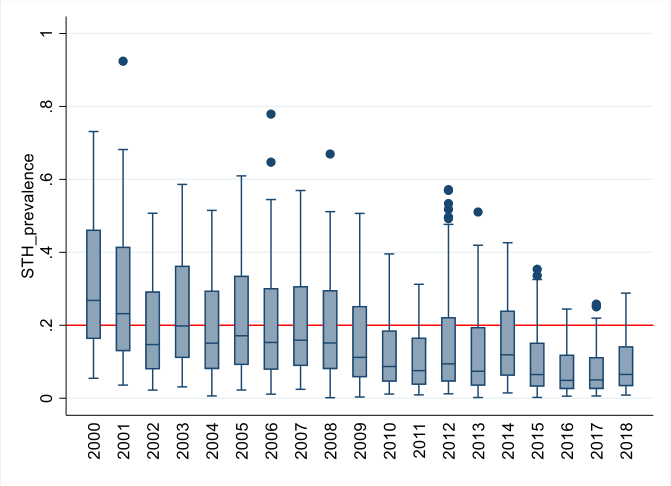
STH_prevalence
0
.2
.4
.6
.8
1
2000 2001 2002 2003 2004 2005 2006 2007 2008 2009 2010 2011 2012 2013 2014 2015 2016 2017 2018

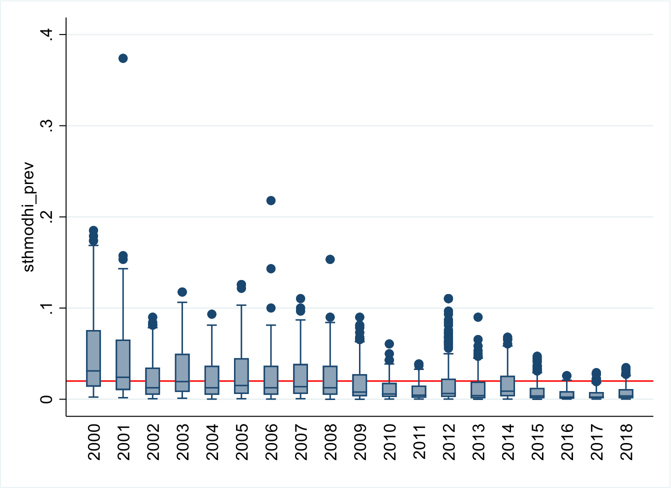
sthmodhi_prev
0
.1
.2
.3
.4
2000 2001 2002 2003 2004 2005 2006 2007 2008 2009 2010 2011 2012 2013 2014 2015 2016 2017 2018

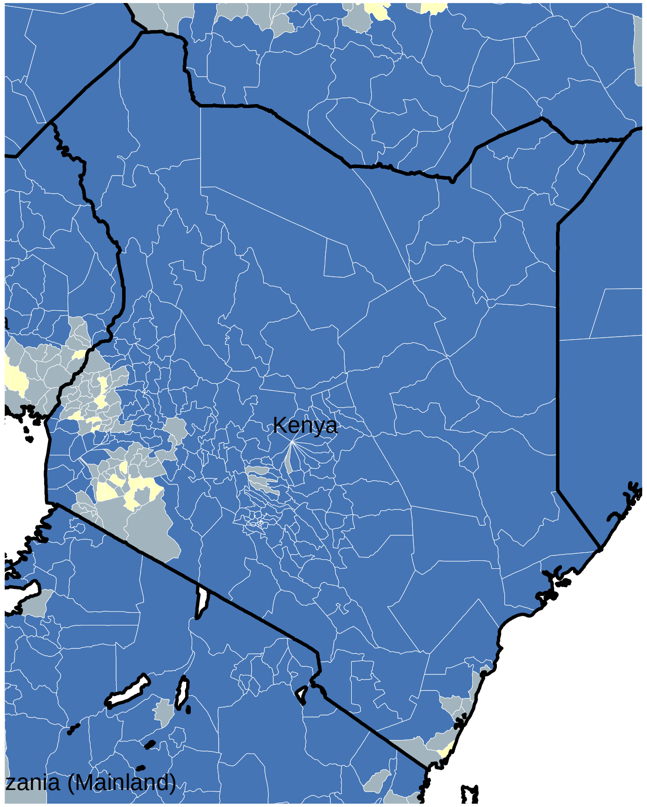
Kenya
zania (Mainland)

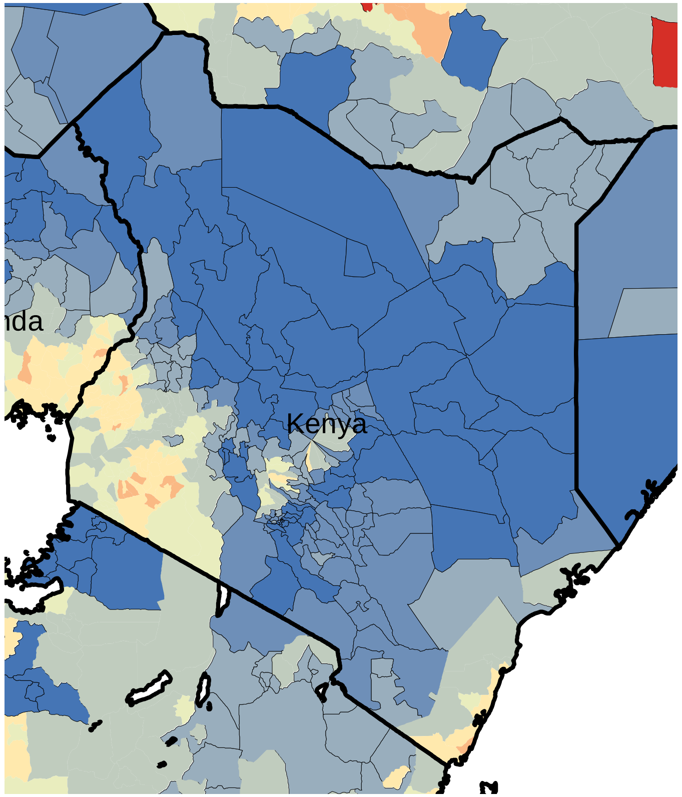
nda
Kenya

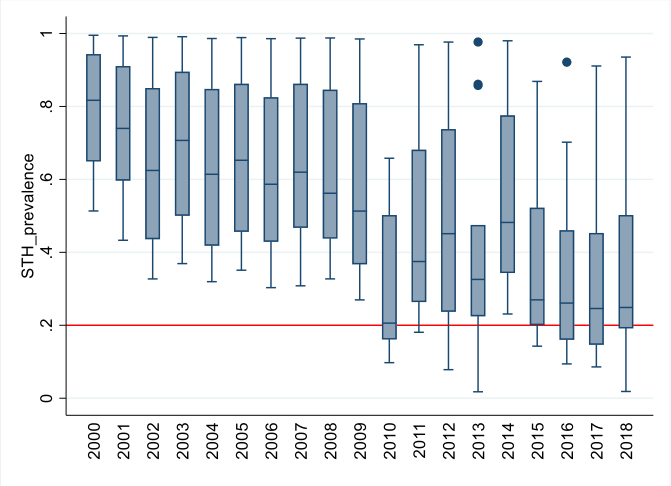
STH_prevalence
0
.2
.4
.6
.8
1
2000
2001
2002
2003
2004
2005
2006
2007
2008
2009
2010
2011
2012
2013
2014
2015
2016
2017
2018

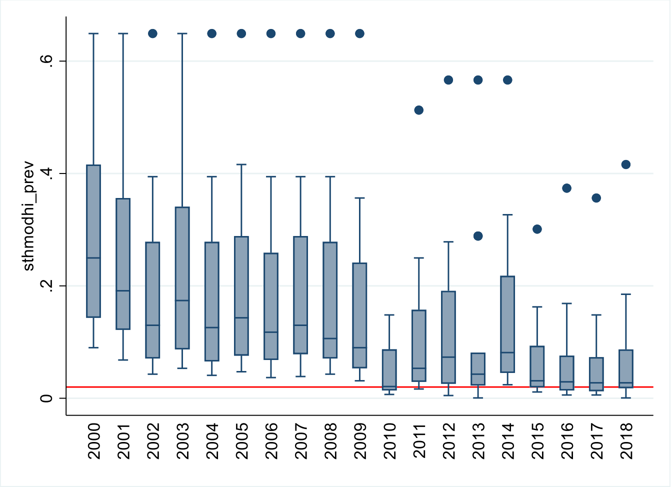
sthmodhi_prev
0
.2
.4
.6
2000
2001
2002
2003
2004
2005
2006
2007
2008
2009
2010
2011
2012
2013
2014
2015
2016
2017
2018

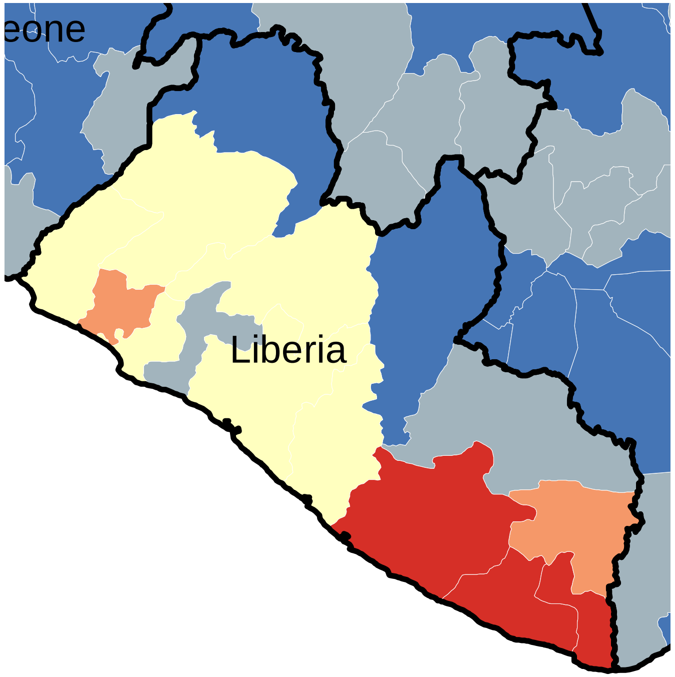
eone
Liberia

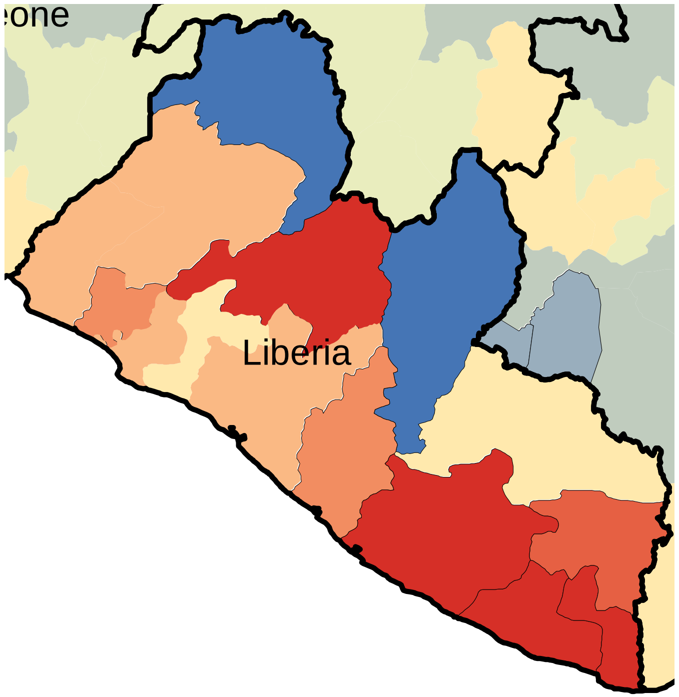
eone
Liberia

LBR

LSO	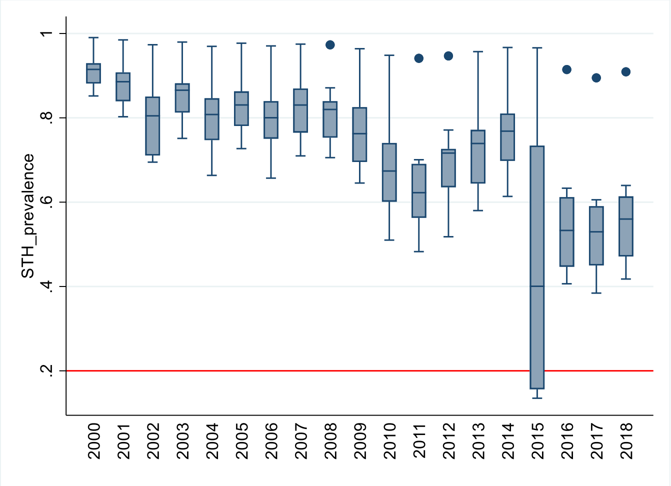STH_prevalence
.2
.4
.6
.8
1
2000 2001 2002 2003 2004 2005 2006 2007 2008 2009 2010 2011 2012 2013 2014 2015 2016 2017 2018 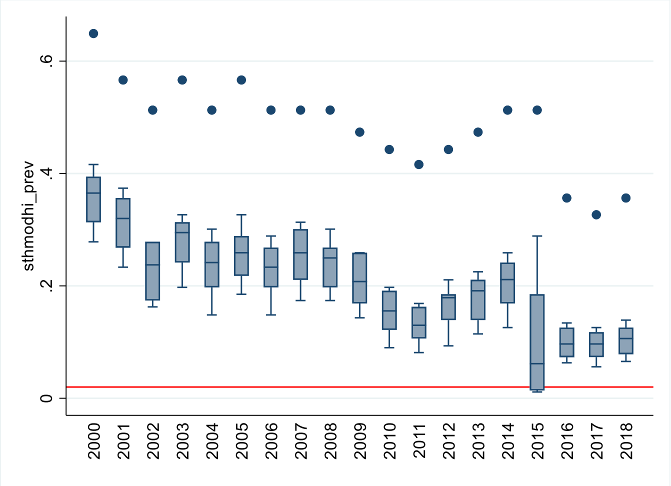sthmodhi_prev
0
.2
.4
.6
2000 2001 2002 2003 2004 2005 2006 2007 2008 2009 2010 2011 2012 2013 2014 2015 2016 2017 2018	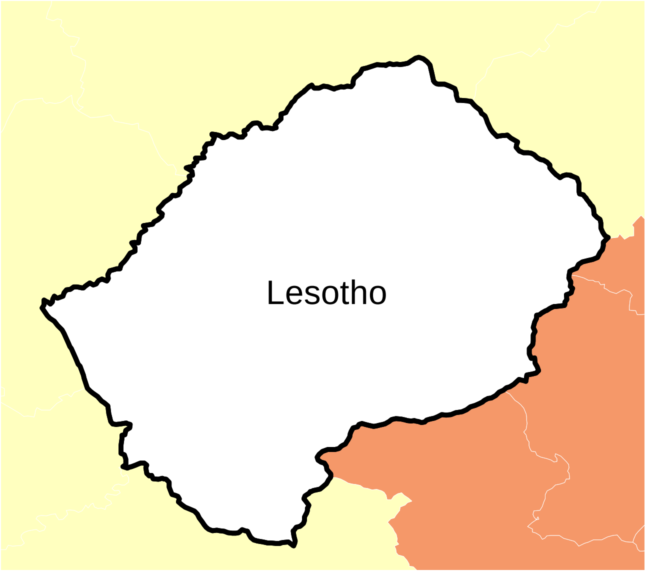Lesotho	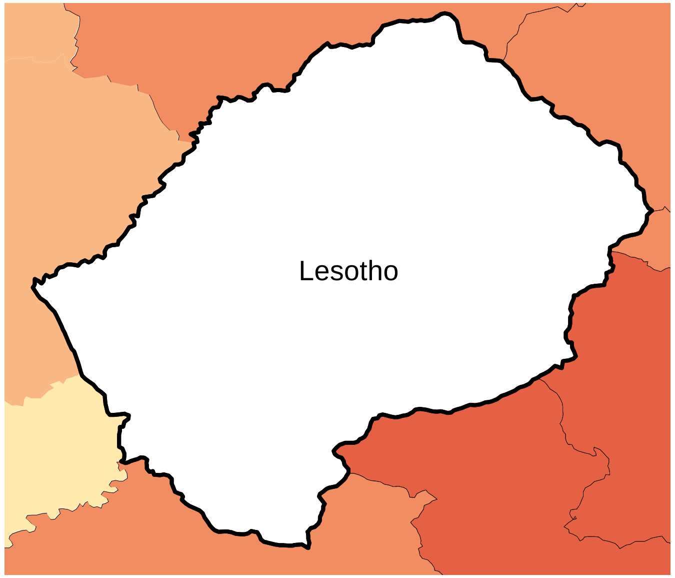Lesotho

MDG

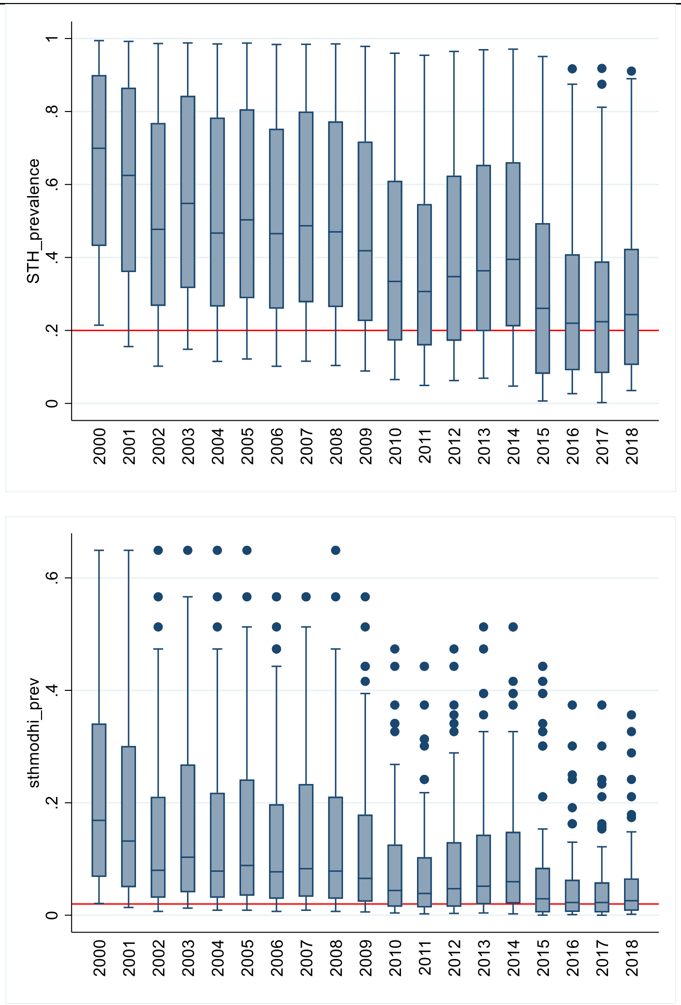
STH_prevalence
sthmodhi_prev
2000
2001
2002
2003
2004
2005
2006
2007
2008
2009
2010
2011
2012
2013
2014
2015
2016
2017
2018

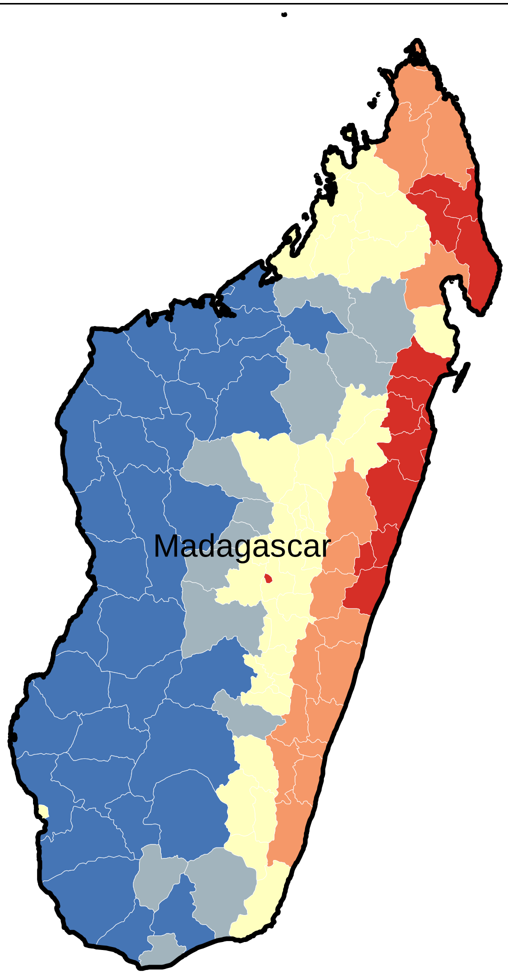
Madagascar

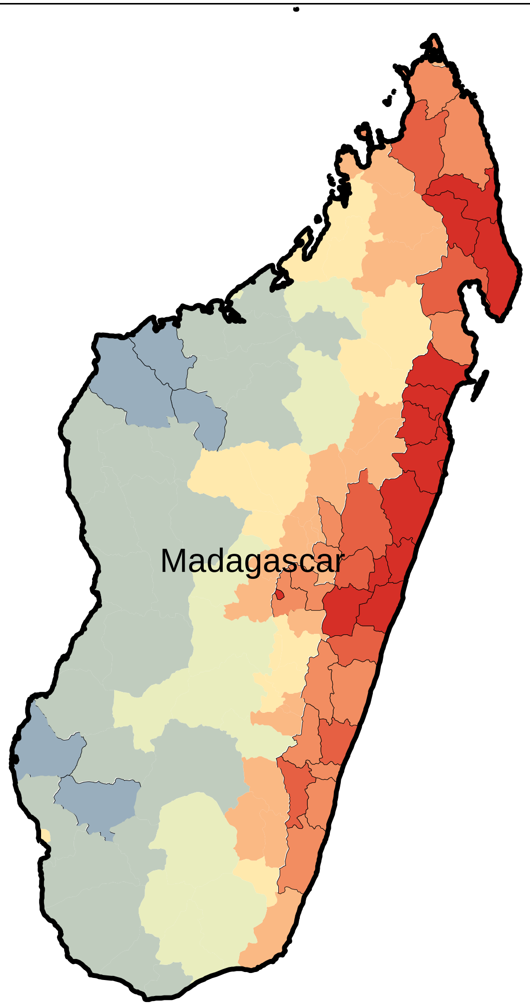
Madagascar

**MLI**	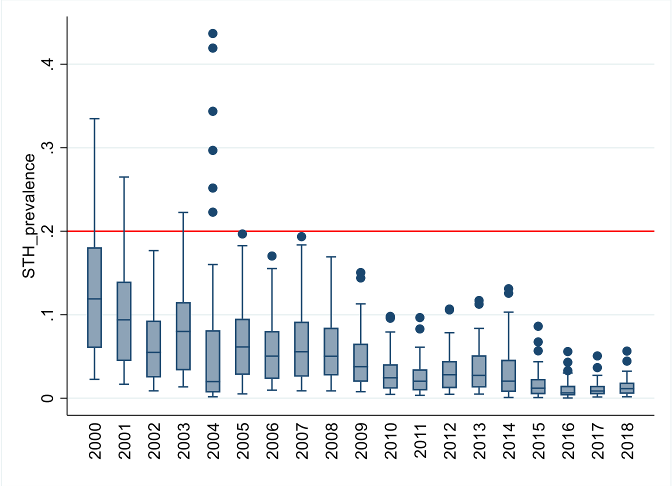 STH_prevalence<br>0<br>.1<br>.2<br>.3<br>.4<br>2000<br>2001<br>2002<br>2003<br>2004<br>2005<br>2006<br>2007<br>2008<br>2009<br>2010<br>2011<br>2012<br>2013<br>2014<br>2015<br>2016<br>2017<br>2018 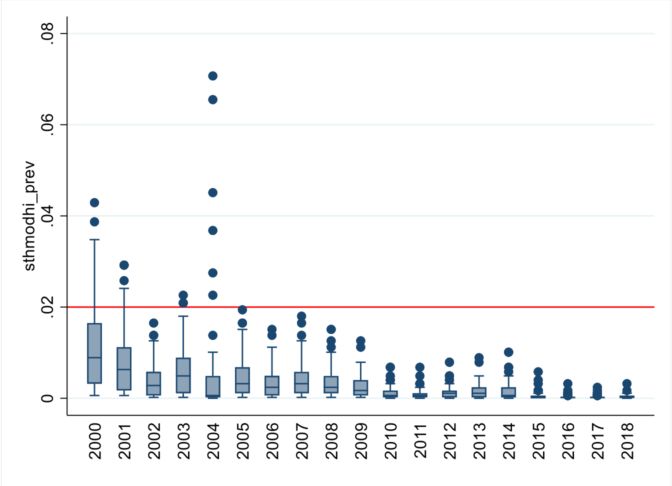 sthmodhi_prev<br>0<br>.02<br>.04<br>.06<br>.08<br>2000<br>2001<br>2002<br>2003<br>2004<br>2005<br>2006<br>2007<br>2008<br>2009<br>2010<br>2011<br>2012<br>2013<br>2014<br>2015<br>2016<br>2017<br>2018	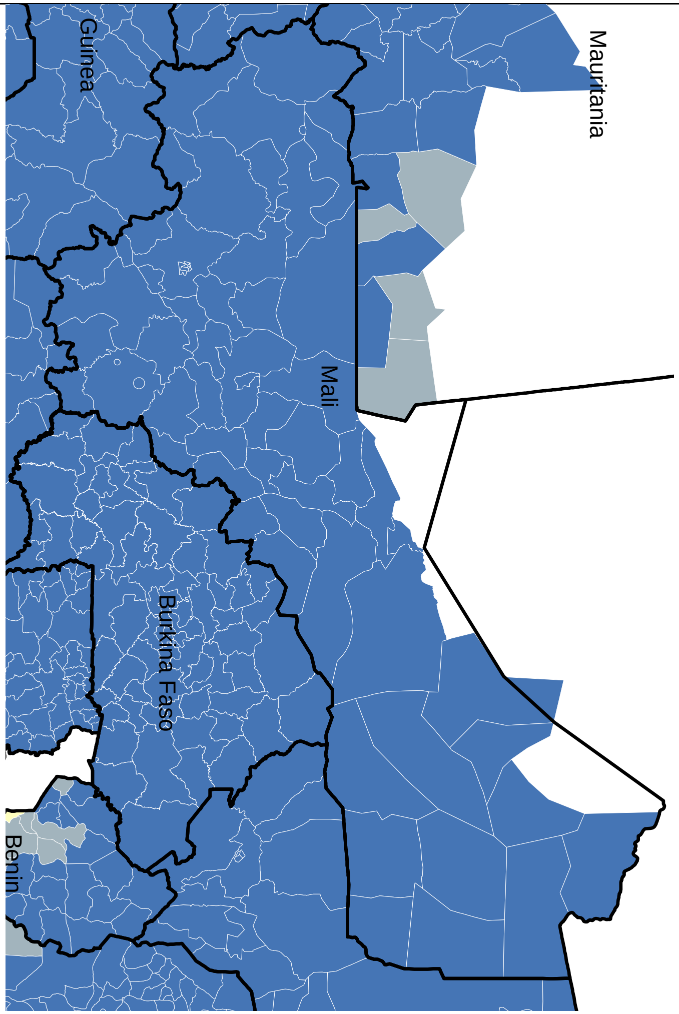 Guinea<br>Mauritania<br>Mali<br>Burkina Faso<br>Benin	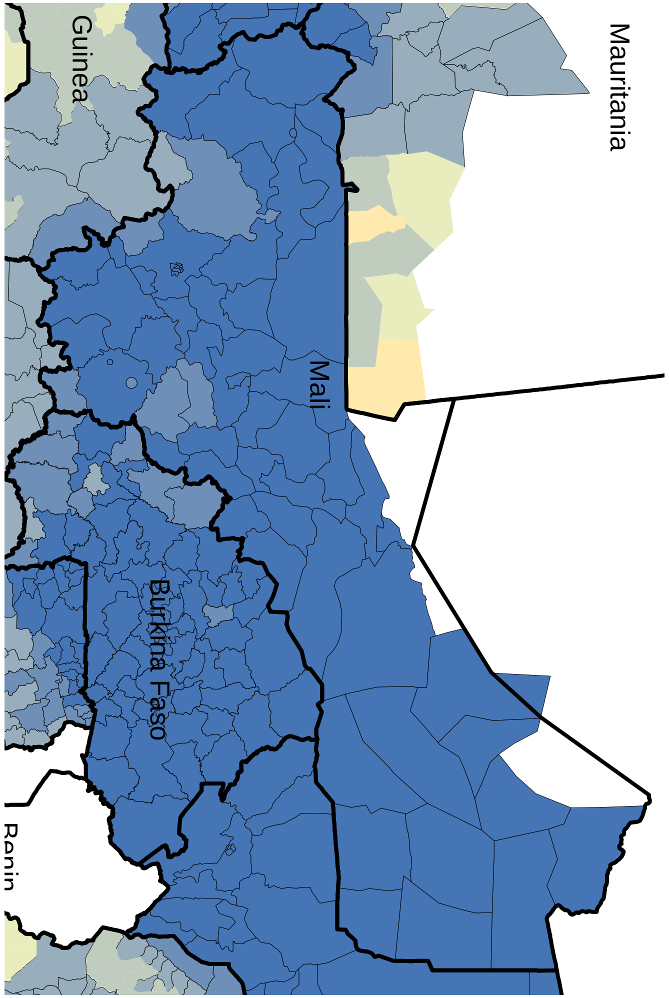 Guinea<br>Mauritania<br>Mali<br>Burkina Faso

MOZ

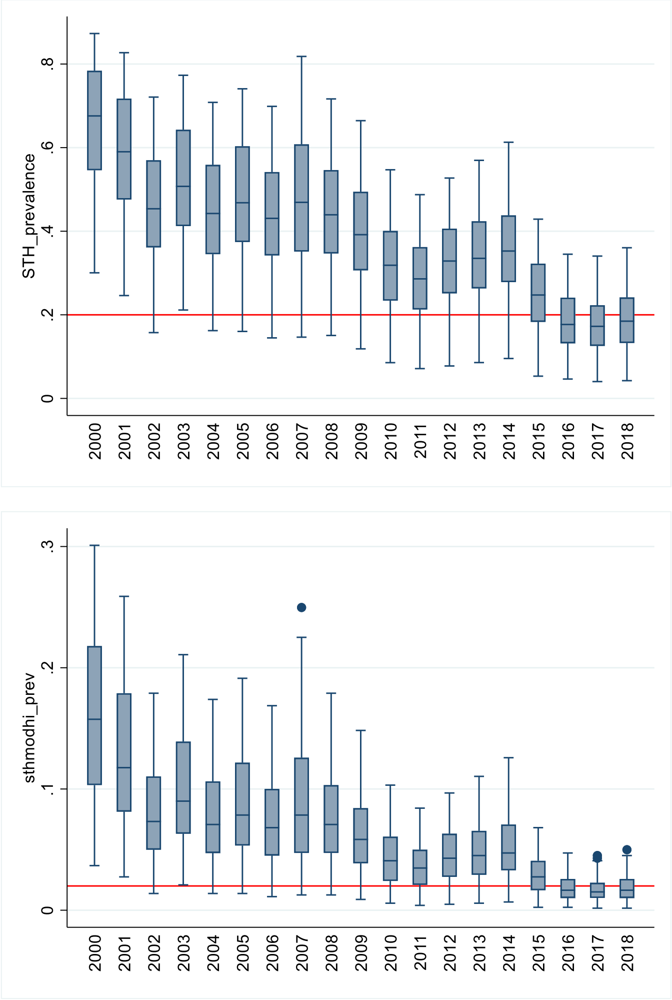
STH_prevalence
0
.2
.4
.6
.8
2000 2001 2002 2003 2004 2005 2006 2007 2008 2009 2010 2011 2012 2013 2014 2015 2016 2017 2018
sthmodhi_prev
0
.1
.2
.3
2000 2001 2002 2003 2004 2005 2006 2007 2008 2009 2010 2011 2012 2013 2014 2015 2016 2017 2018

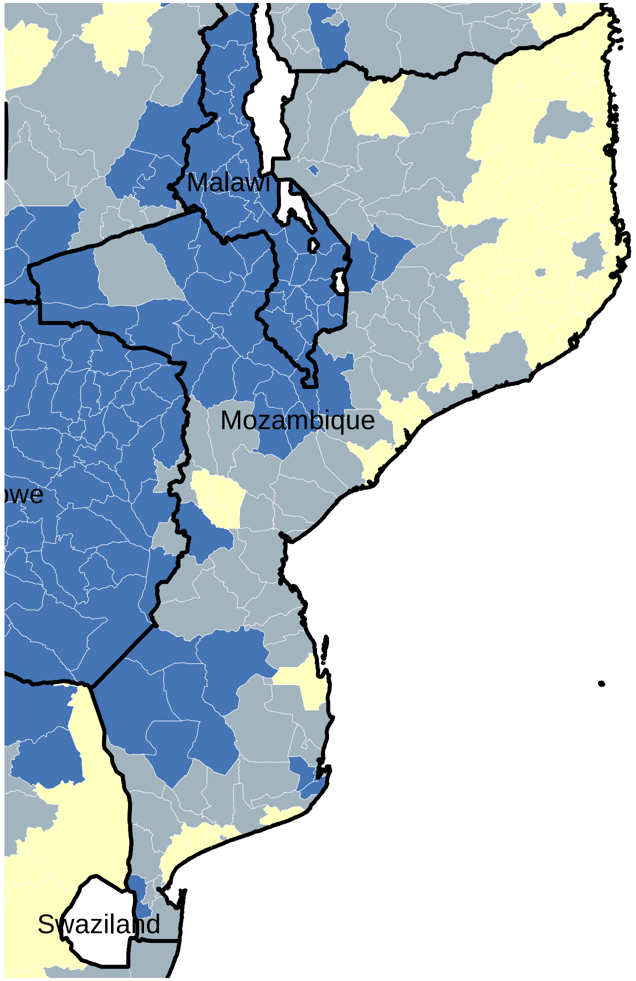
Malawi
Mozambique
bwe
Swaziland

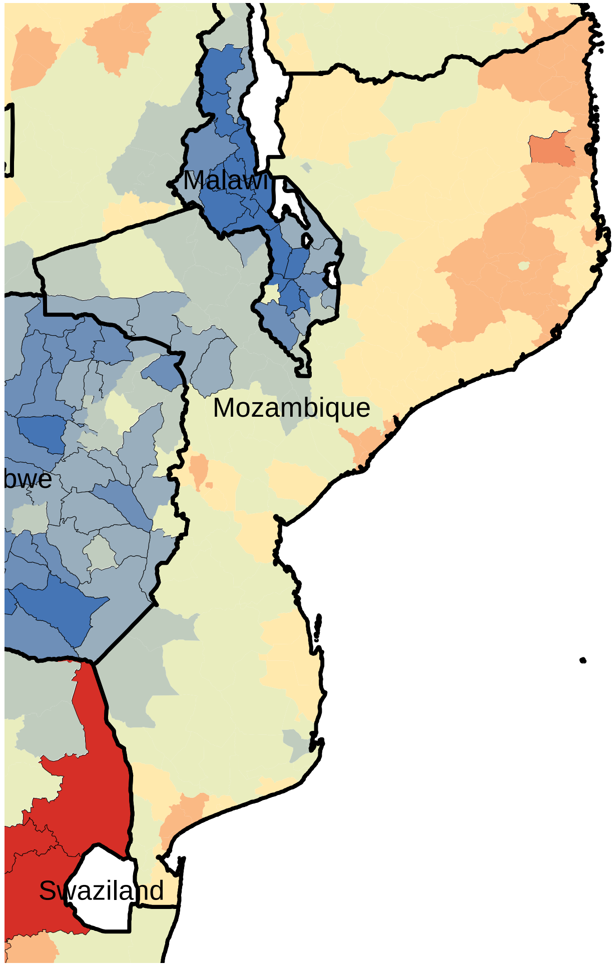
Malawi
Mozambique
bwe
Swaziland

MRT

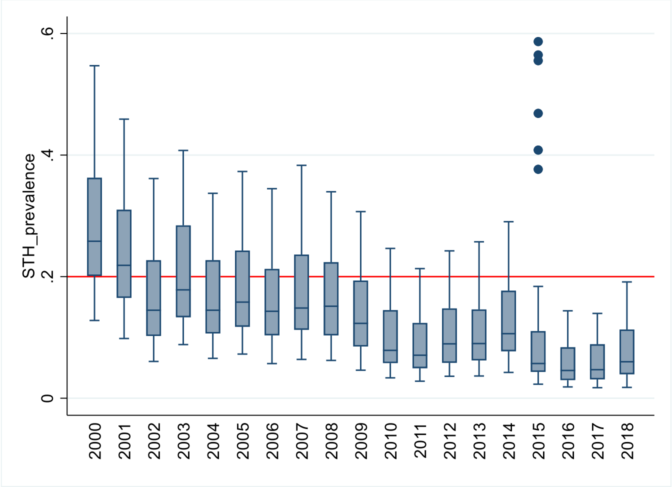
STH_prevalence
0
.2
.4
.6
2000 2001 2002 2003 2004 2005 2006 2007 2008 2009 2010 2011 2012 2013 2014 2015 2016 2017 2018

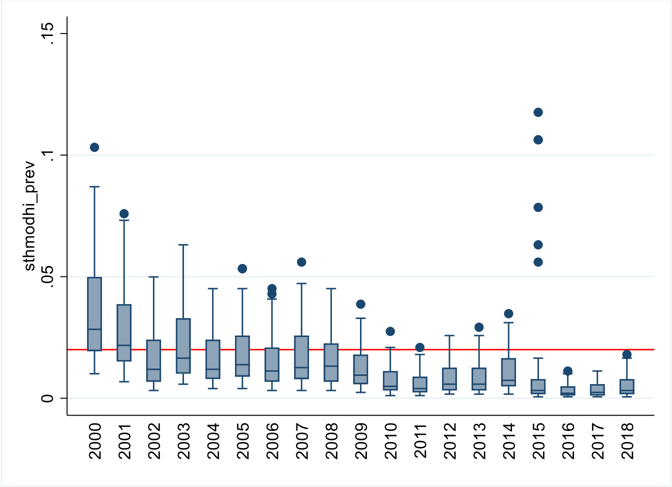
sthmodhi_prev
0
.05
.1
.15
2000 2001 2002 2003 2004 2005 2006 2007 2008 2009 2010 2011 2012 2013 2014 2015 2016 2017 2018

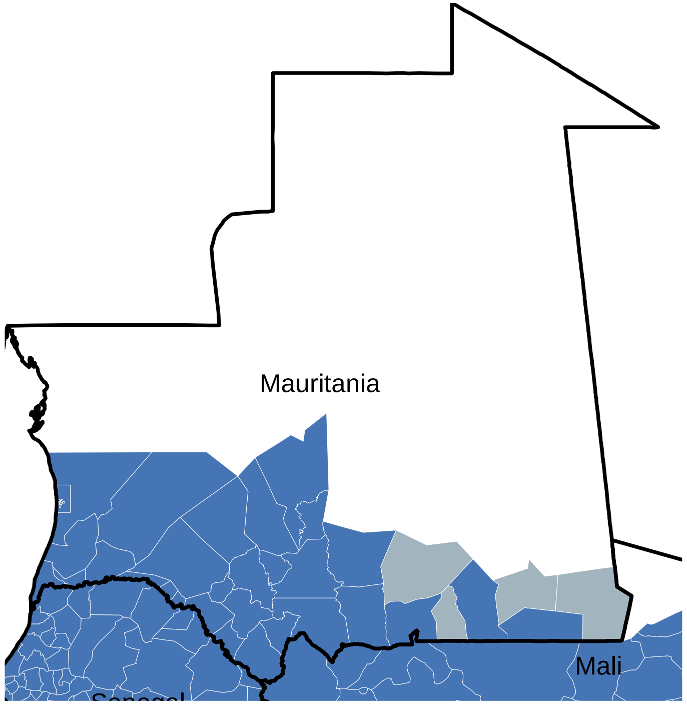
Mauritania
Mali

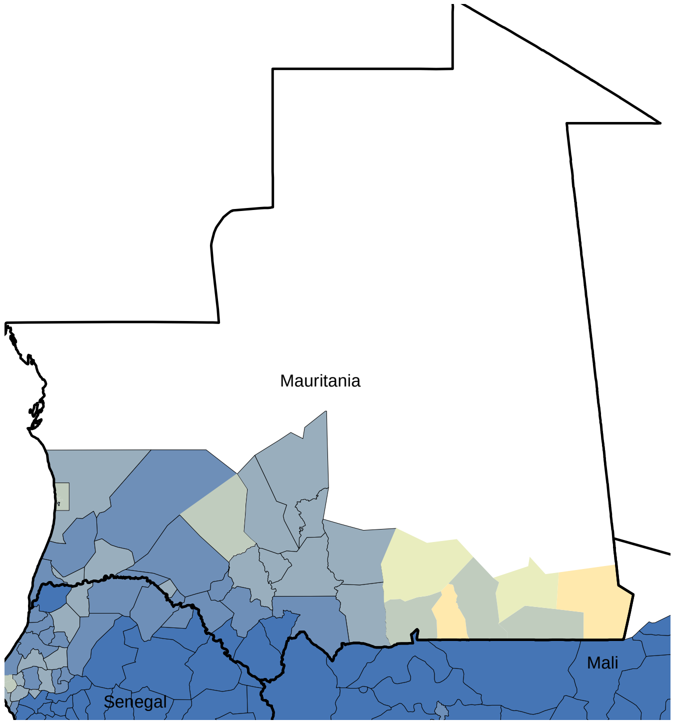
Mauritania
Senegal
Mali

MWI	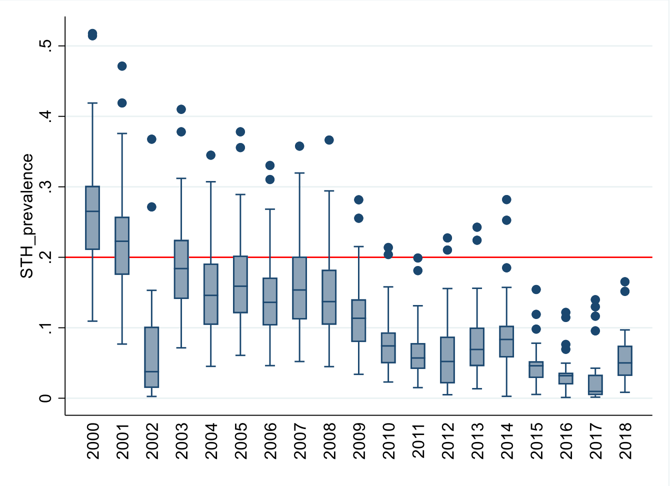STH_prevalence
0
.1
.2
.3
.4
.5
2000
2001
2002
2003
2004
2005
2006
2007
2008
2009
2010
2011
2012
2013
2014
2015
2016
2017
2018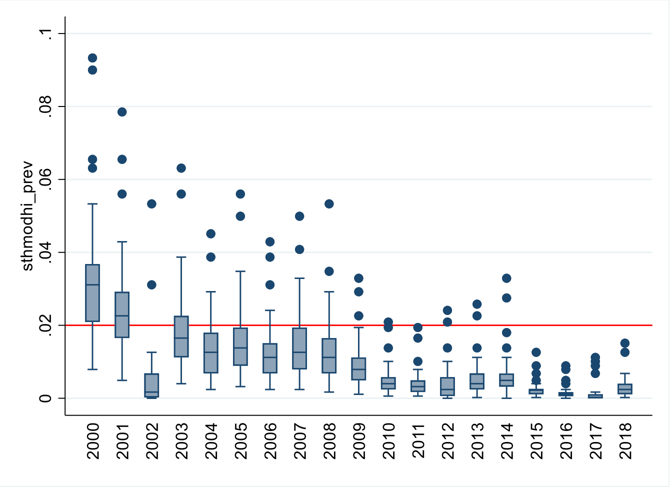sthmodhi_prev
0
.02
.04
.06
.08
.1
2000
2001
2002
2003
2004
2005
2006
2007
2008
2009
2010
2011
2012
2013
2014
2015
2016
2017
2018	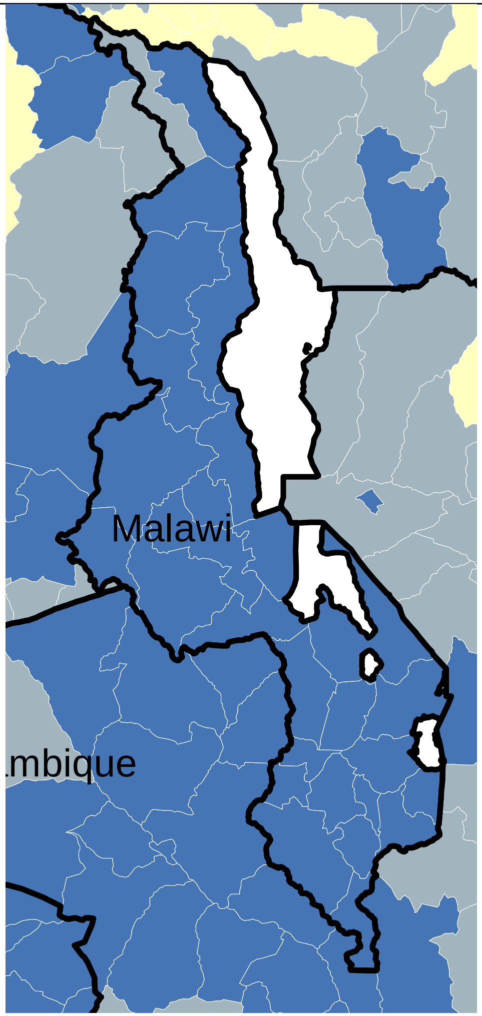Malawi
.mbique	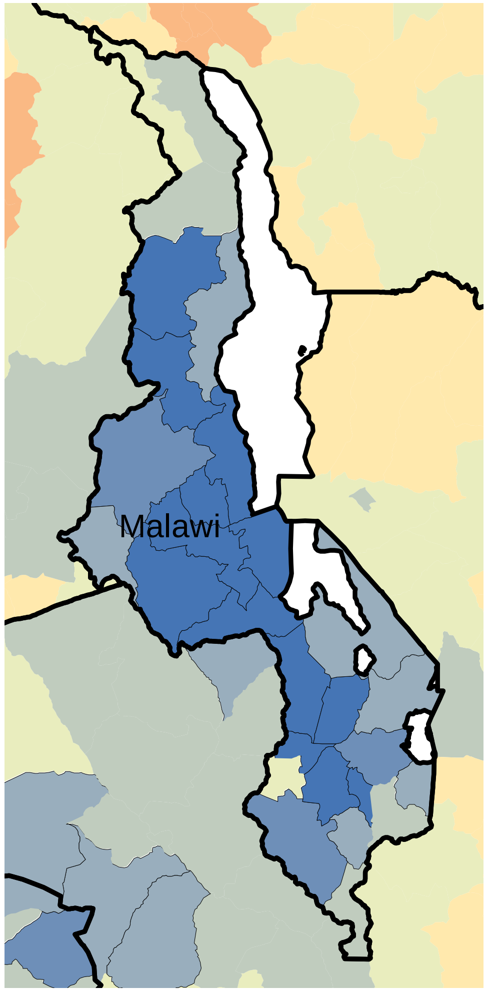Malawi

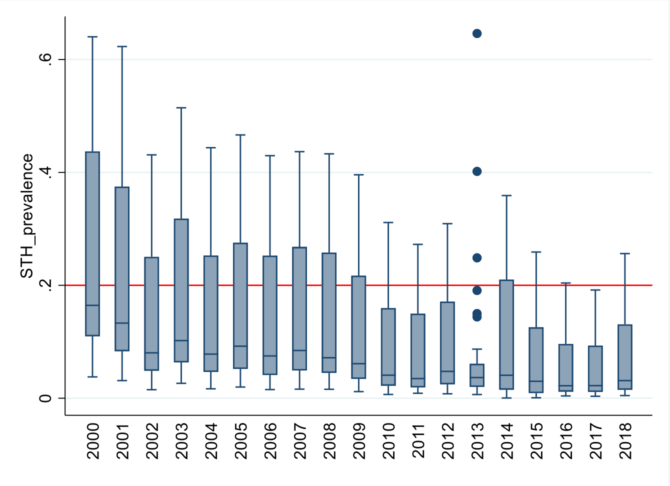
STH_prevalence
0
.2
.4
.6
2000
2001
2002
2003
2004
2005
2006
2007
2008
2009
2010
2011
2012
2013
2014
2015
2016
2017
2018

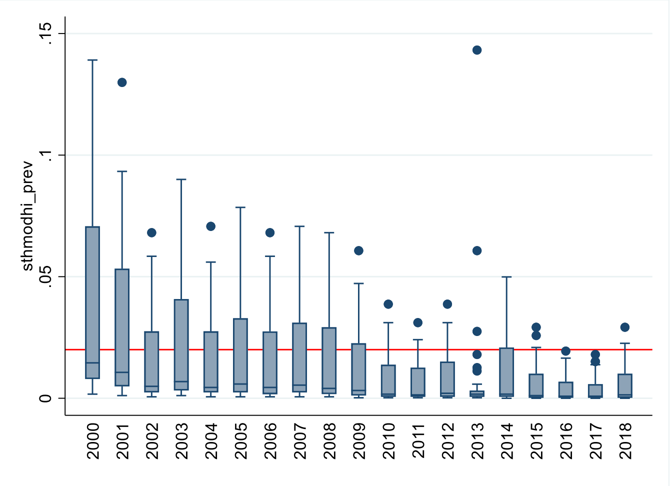
sthmodhi_prev
0
.05
.1
.15
2000
2001
2002
2003
2004
2005
2006
2007
2008
2009
2010
2011
2012
2013
2014
2015
2016
2017
2018

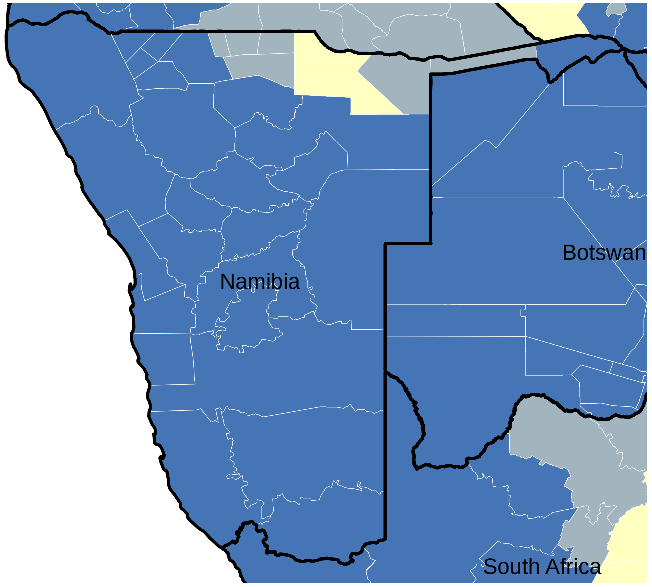
Namibia
Botswan
South Africa

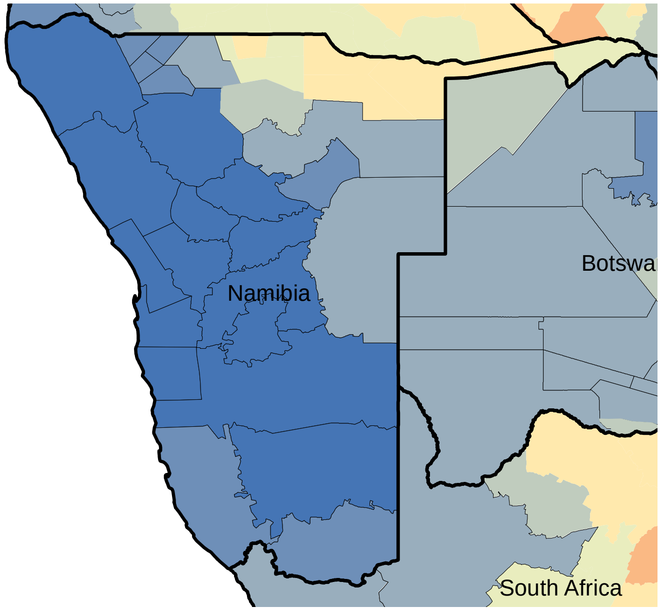
Namibia
Botswa
South Africa

NAM

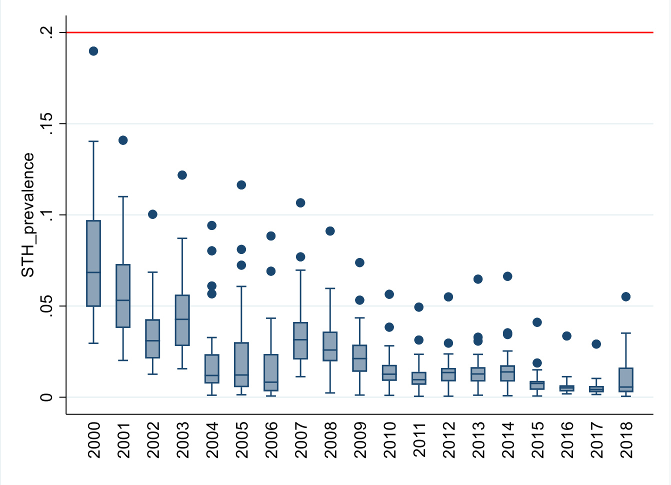
STH_prevalence
0
.05
.1
.15
.2
2000 2001 2002 2003 2004 2005 2006 2007 2008 2009 2010 2011 2012 2013 2014 2015 2016 2017 2018

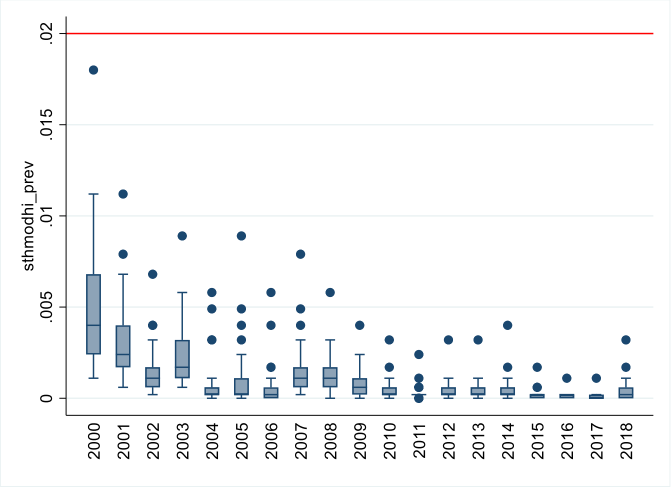
sthmodhi_prev
0
.005
.01
.015
.02
2000 2001 2002 2003 2004 2005 2006 2007 2008 2009 2010 2011 2012 2013 2014 2015 2016 2017 2018

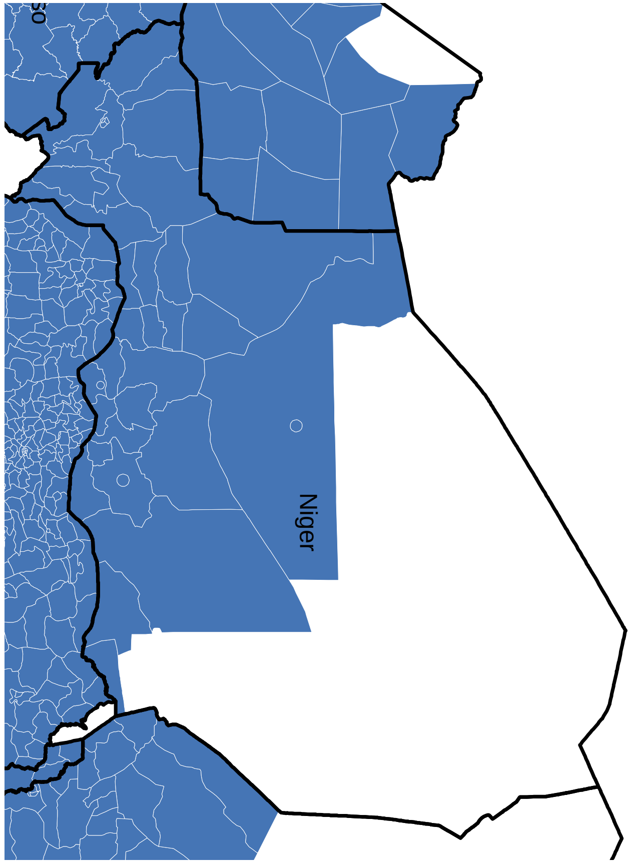
Niger

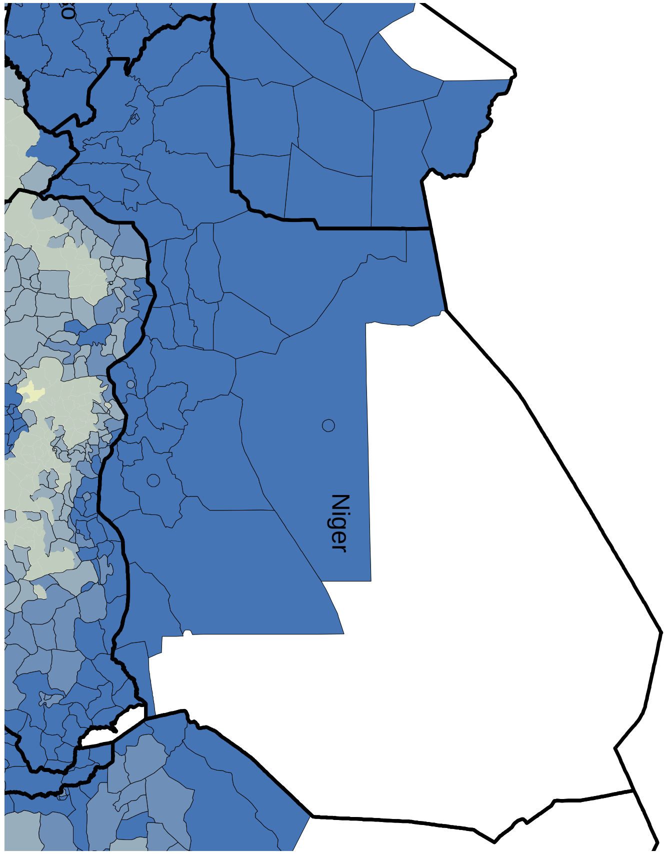
Niger

NER

NGA

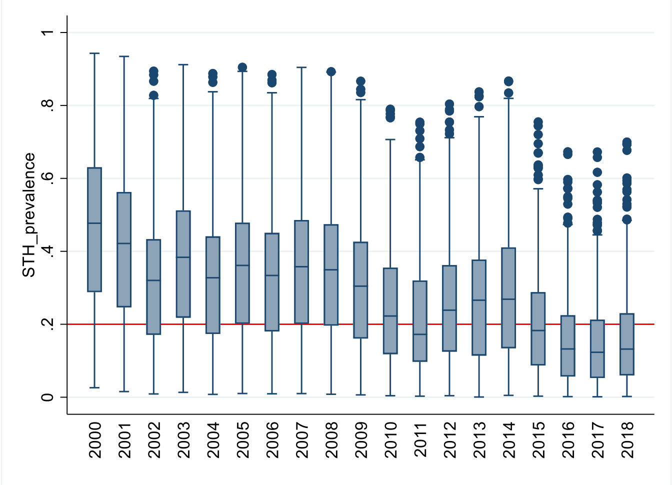
STH_prevalence
0
.2
.4
.6
.8
1
2000 2001 2002 2003 2004 2005 2006 2007 2008 2009 2010 2011 2012 2013 2014 2015 2016 2017 2018

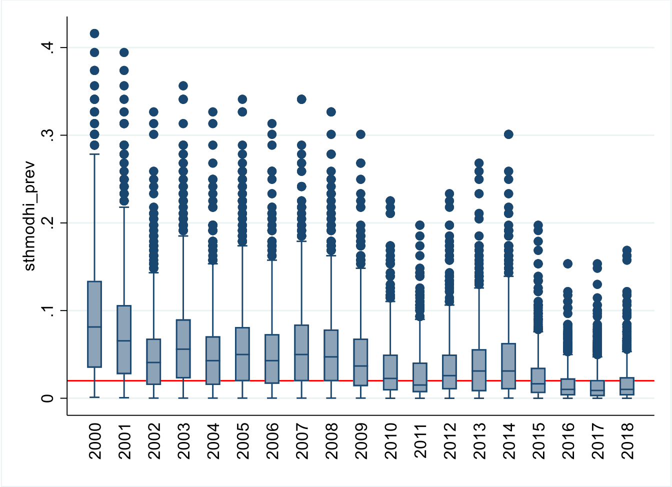
sthmodhi_prev
0
.1
.2
.3
.4
2000 2001 2002 2003 2004 2005 2006 2007 2008 2009 2010 2011 2012 2013 2014 2015 2016 2017 2018

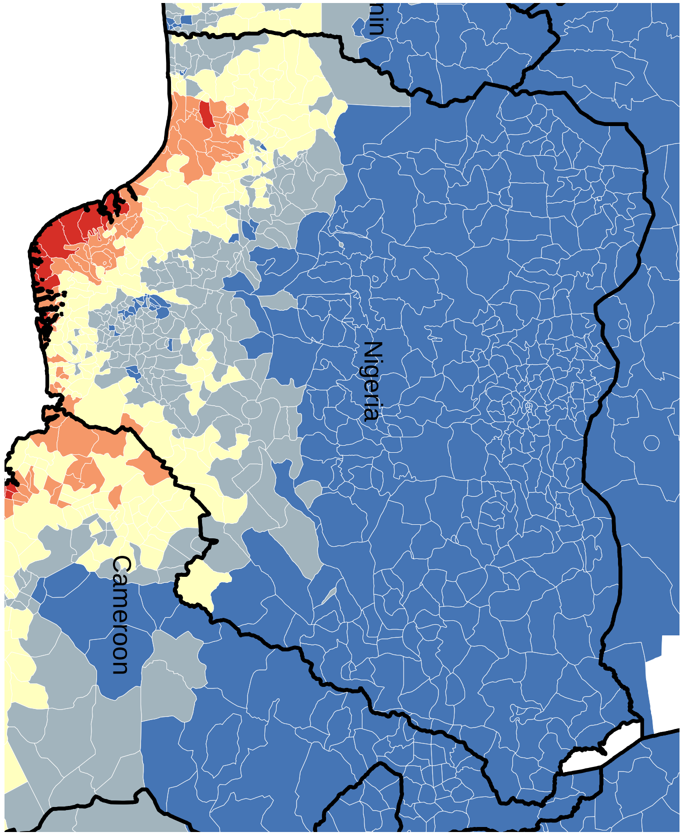
Nigeria
Cameroon

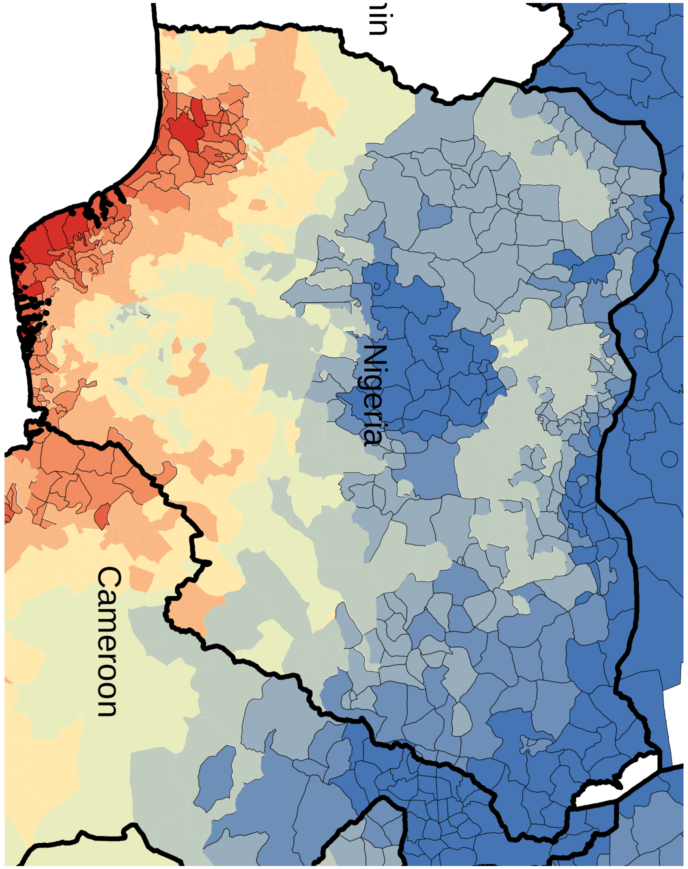
Nigeria
Cameroon

RWA

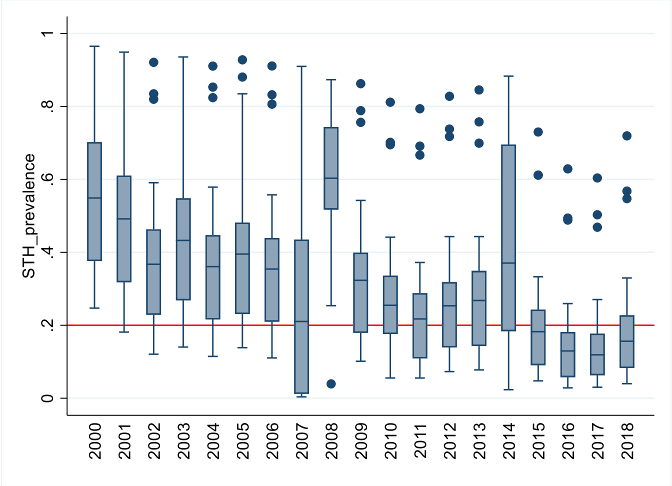
STH_prevalence
0
.2
.4
.6
.8
1
2000
2001
2002
2003
2004
2005
2006
2007
2008
2009
2010
2011
2012
2013
2014
2015
2016
2017
2018

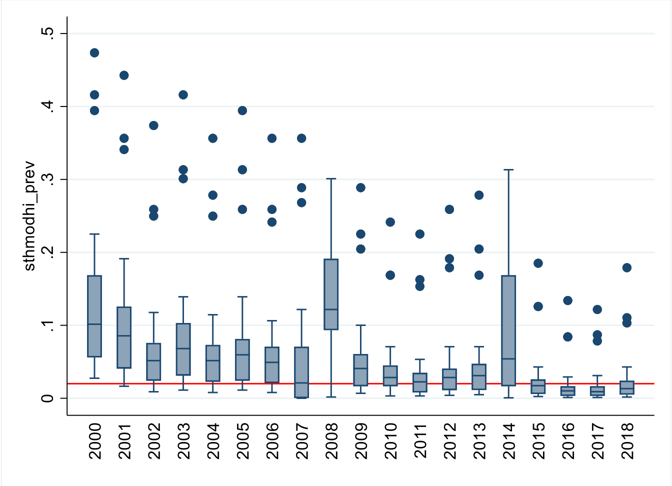
sthmodhi_prev
0
.1
.2
.3
.4
.5
2000
2001
2002
2003
2004
2005
2006
2007
2008
2009
2010
2011
2012
2013
2014
2015
2016
2017
2018

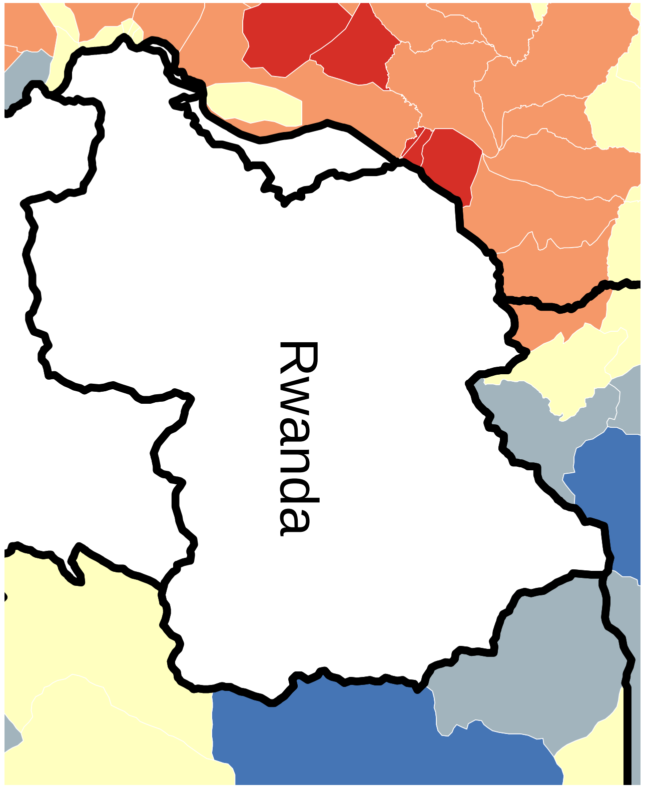
Rwanda

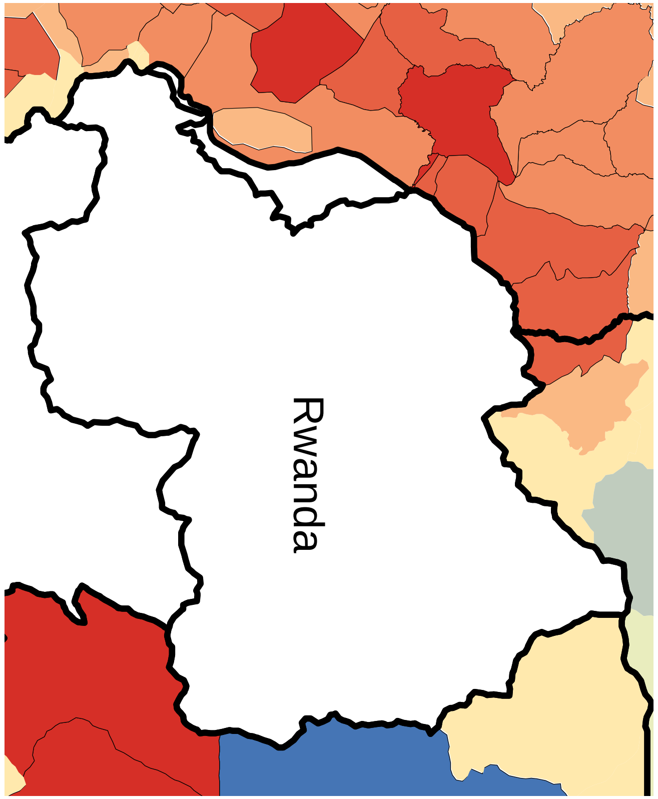
Rwanda

SDN

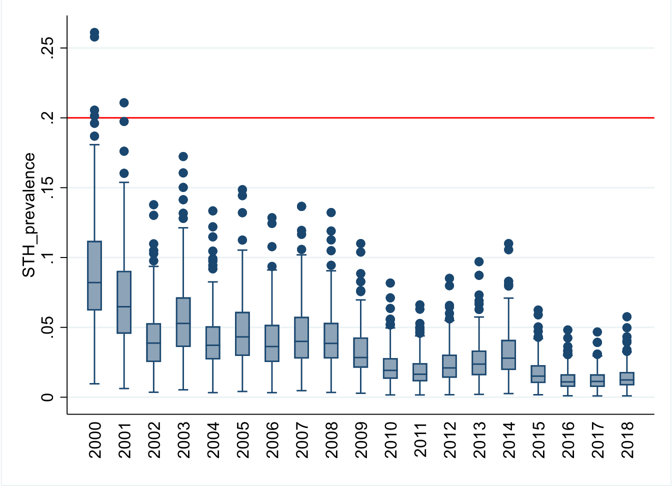
STH_prevalence
0
.05
.1
.15
.2
.25
2000 2001 2002 2003 2004 2005 2006 2007 2008 2009 2010 2011 2012 2013 2014 2015 2016 2017 2018

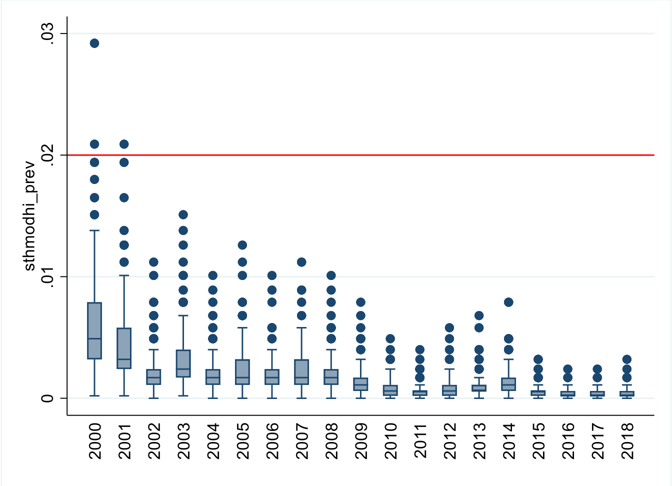
sthmodhi_prev
0
.01
.02
.03
2000 2001 2002 2003 2004 2005 2006 2007 2008 2009 2010 2011 2012 2013 2014 2015 2016 2017 2018

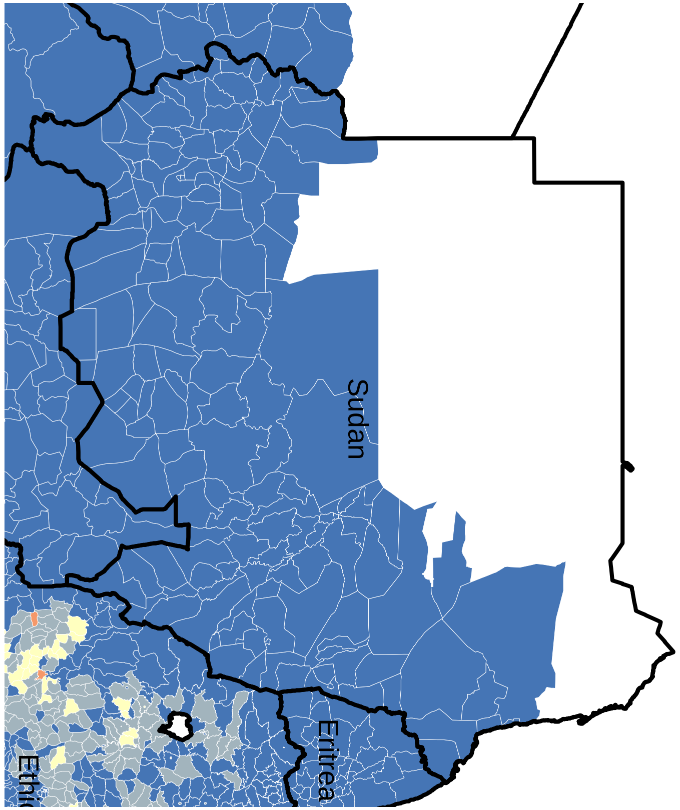
Sudan
Eritrea

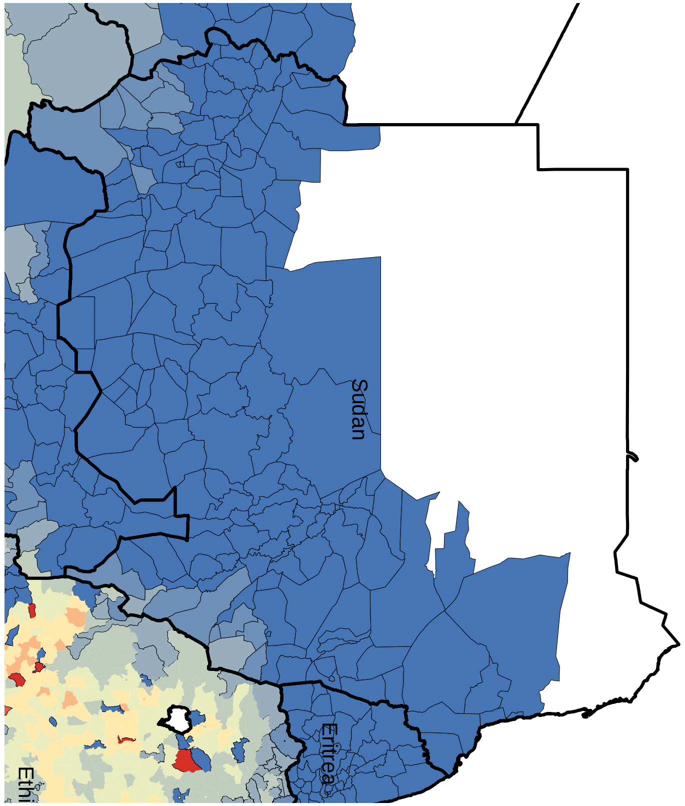
Sudan
Eritrea

SEN

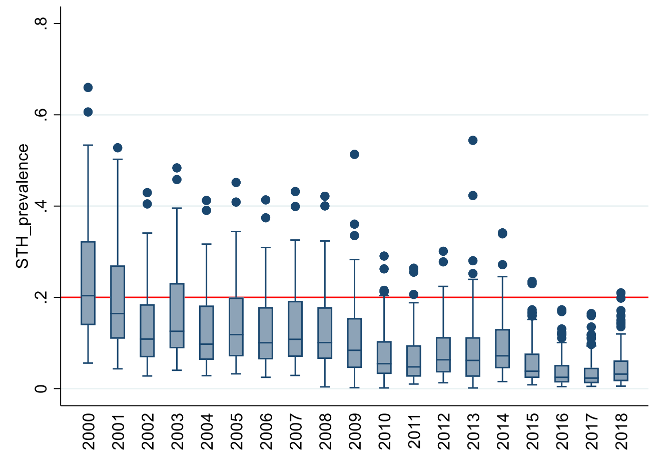
STH_prevalence
0
.2
.4
.6
.8
2000
2001
2002
2003
2004
2005
2006
2007
2008
2009
2010
2011
2012
2013
2014
2015
2016
2017
2018

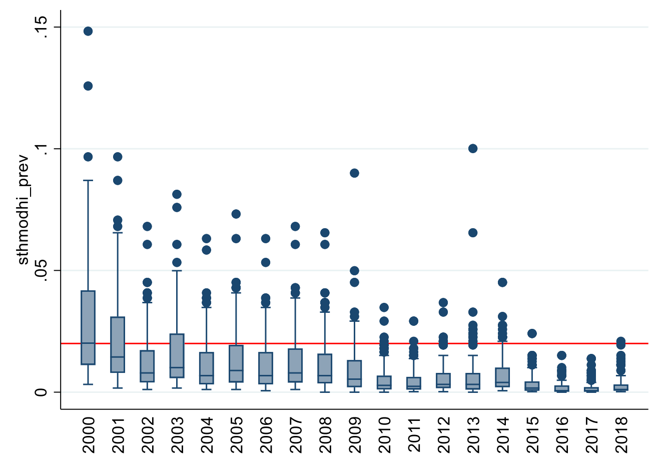
sthmodhi_prev
0
.05
.1
.15
2000
2001
2002
2003
2004
2005
2006
2007
2008
2009
2010
2011
2012
2013
2014
2015
2016
2017
2018

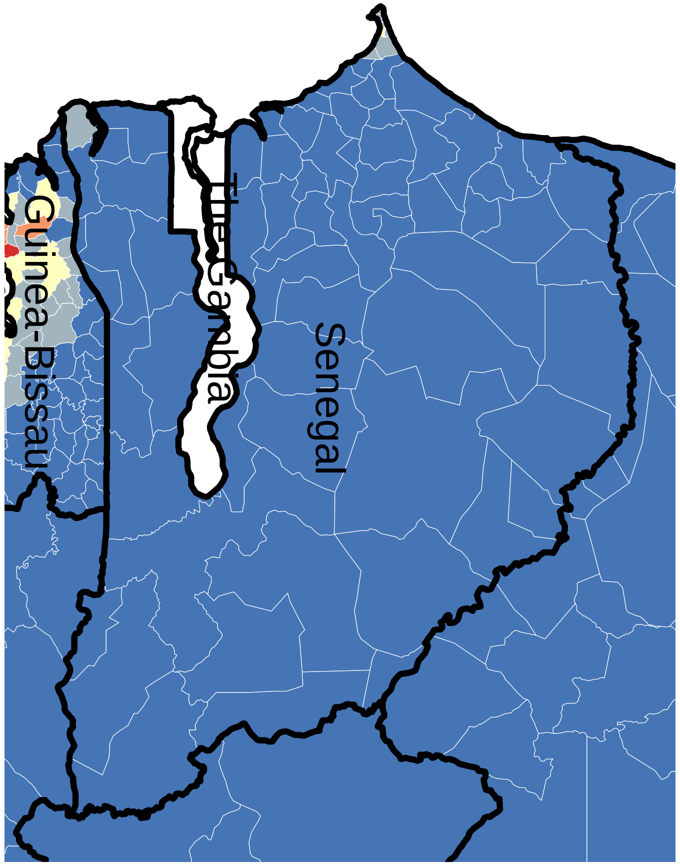
Guinea-Bissau
The Gambia
Senegal

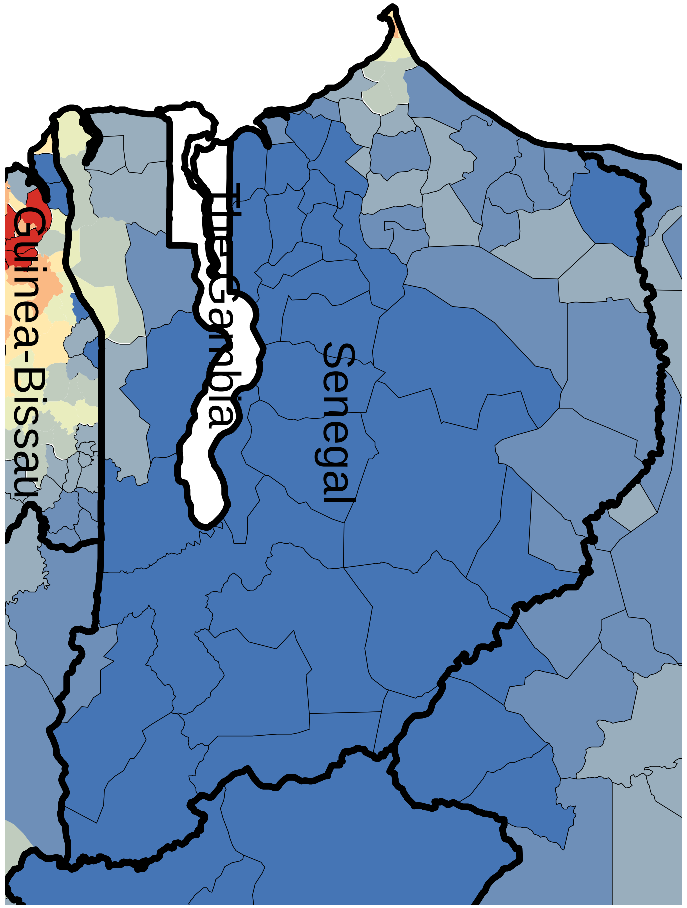
Guinea-Bissau
The Gambia
Senegal

SLE	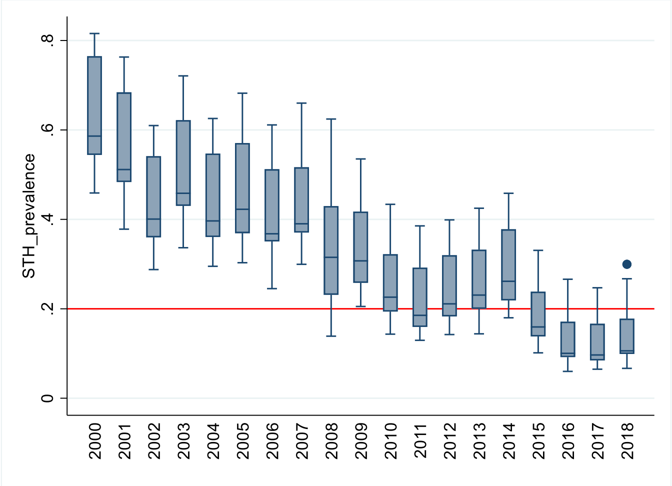 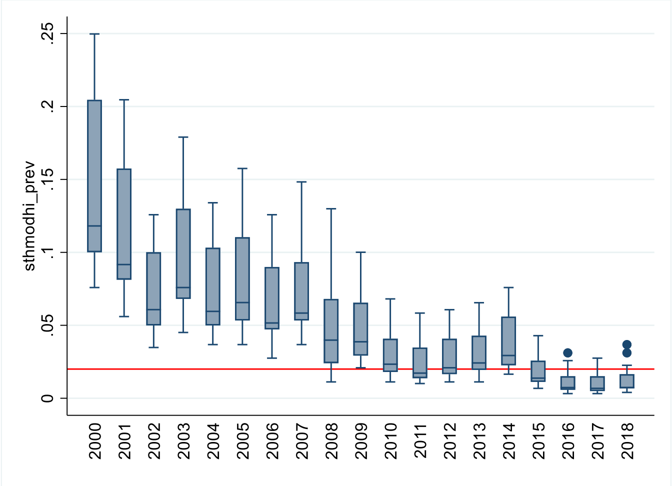	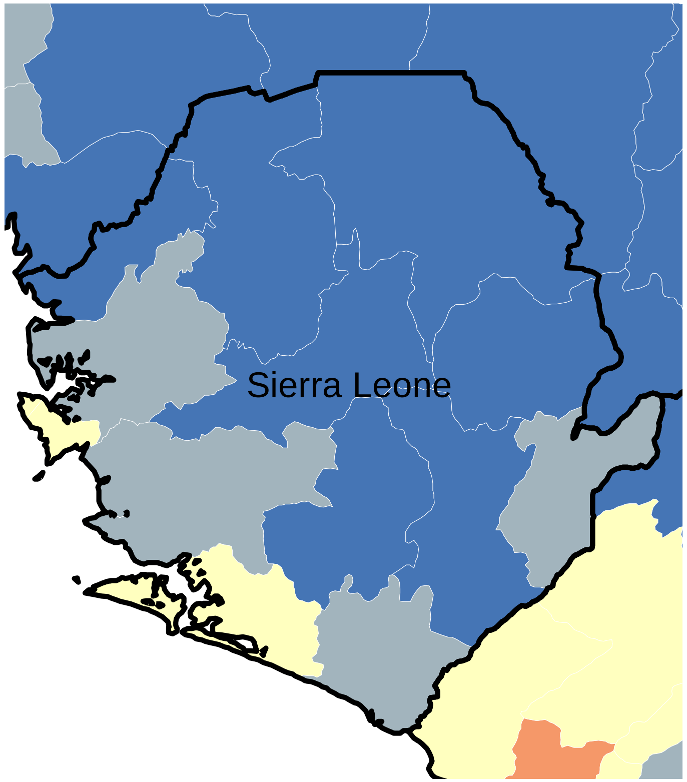	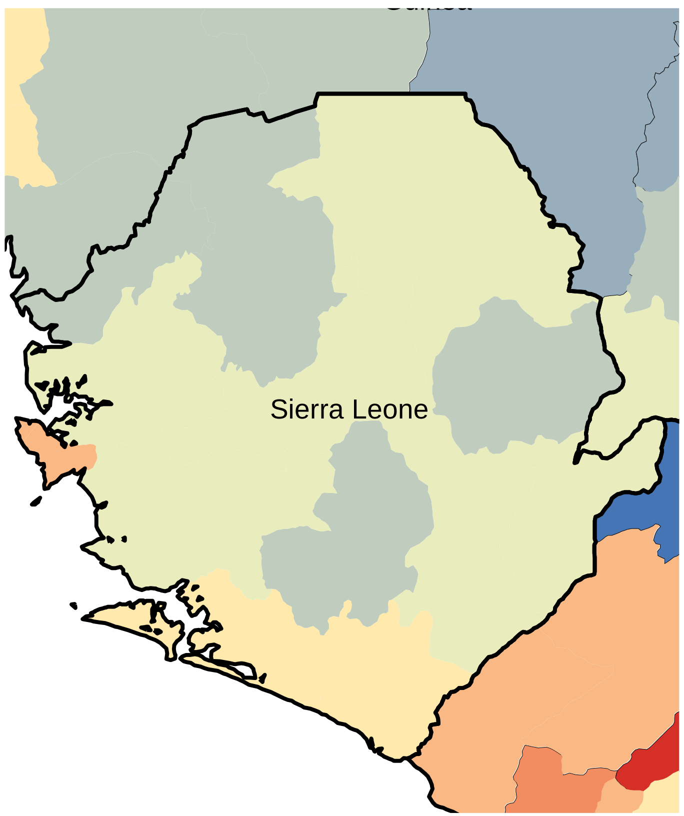

STH_prevalence
0
.2
.4
.6
.8
2000 2001 2002 2003 2004 2005 2006 2007 2008 2009 2010 2011 2012 2013 2014 2015 2016 2017 2018

sthmodhi_prev
0
.05
.1
.15
.2
.25
2000 2001 2002 2003 2004 2005 2006 2007 2008 2009 2010 2011 2012 2013 2014 2015 2016 2017 2018

Sierra Leone

Sierra Leone

**SOM**	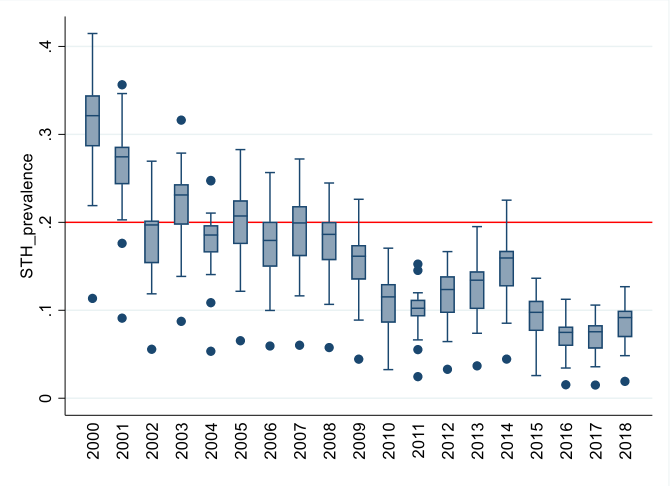STH_prevalence
0
.1
.2
.3
.4
2000 2001 2002 2003 2004 2005 2006 2007 2008 2009 2010 2011 2012 2013 2014 2015 2016 2017 2018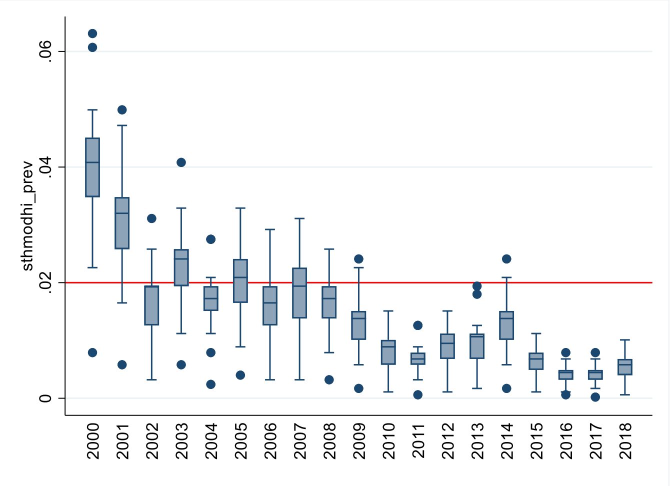sthmodhi_prev
0
.02
.04
.06
2000 2001 2002 2003 2004 2005 2006 2007 2008 2009 2010 2011 2012 2013 2014 2015 2016 2017 2018	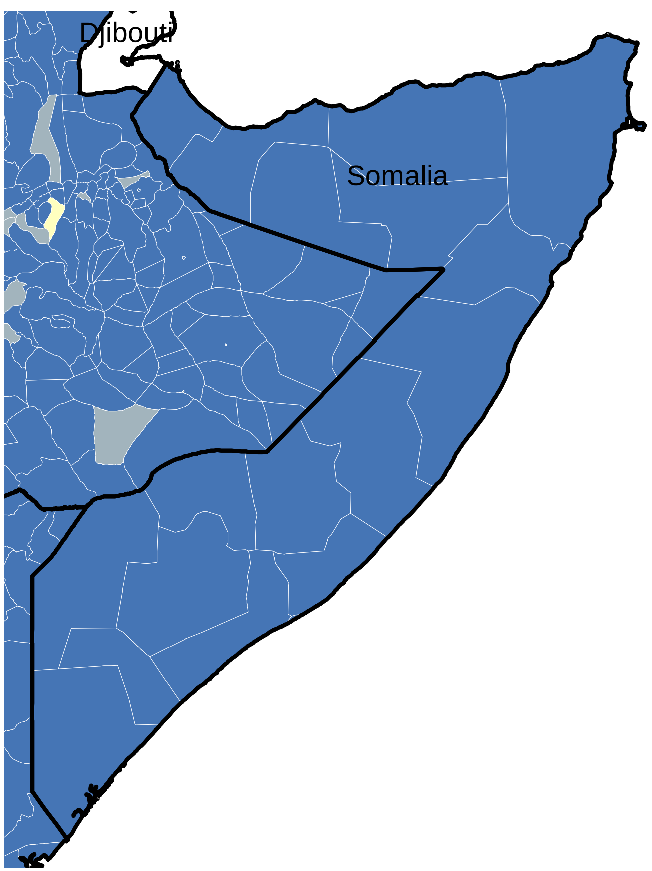Djibouti
Somalia	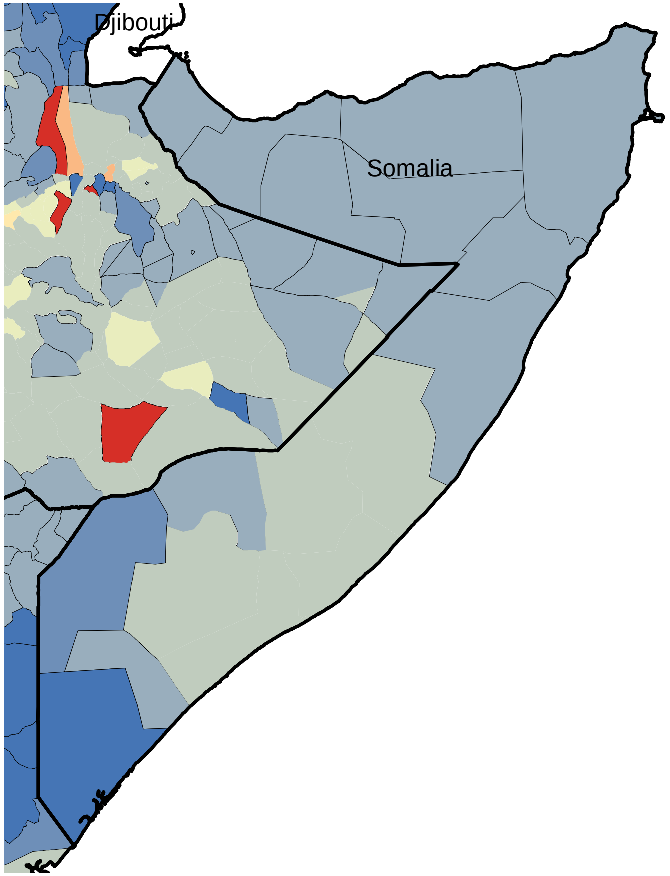Djibouti
Somalia

SSD	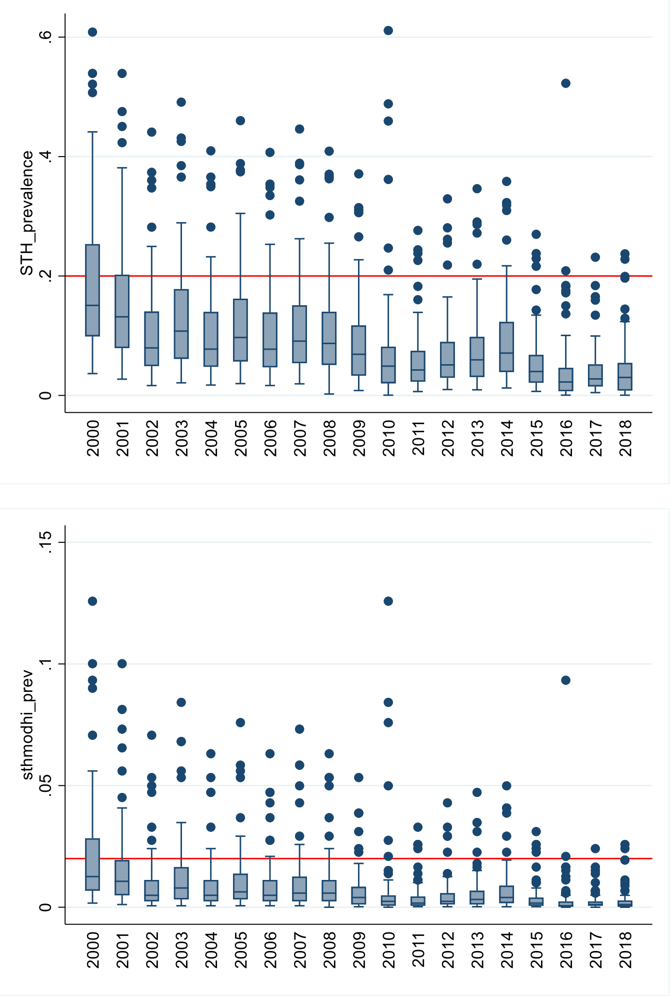 STH_prevalence<br>0<br>.2<br>.4<br>.6<br>2000 2001 2002 2003 2004 2005 2006 2007 2008 2009 2010 2011 2012 2013 2014 2015 2016 2017 2018<br>sthmodhi_prev_<br>0<br>.05<br>.1<br>.15<br>2000 2001 2002 2003 2004 2005 2006 2007 2008 2009 2010 2011 2012 2013 2014 2015 2016 2017 2018	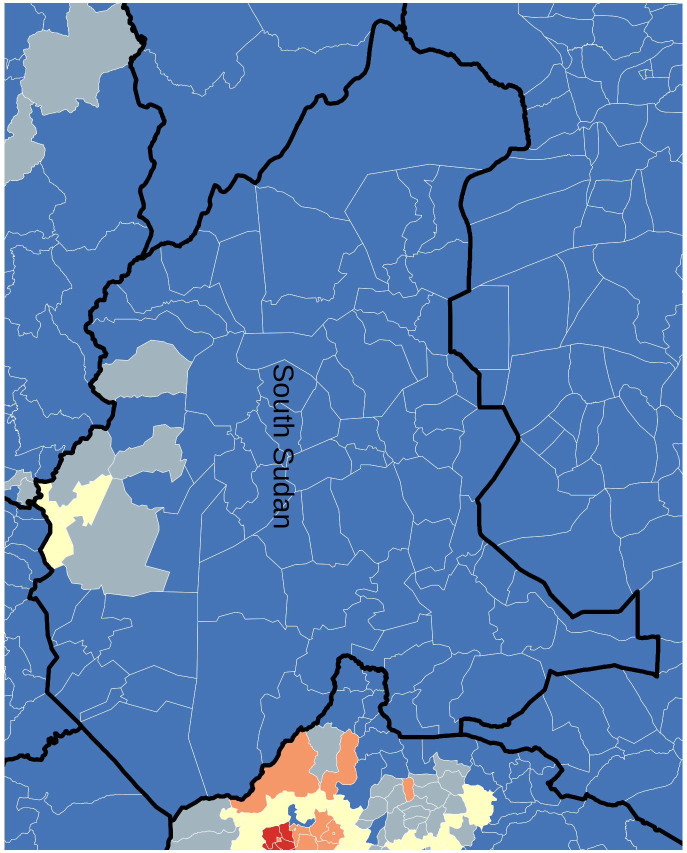 South Sudan	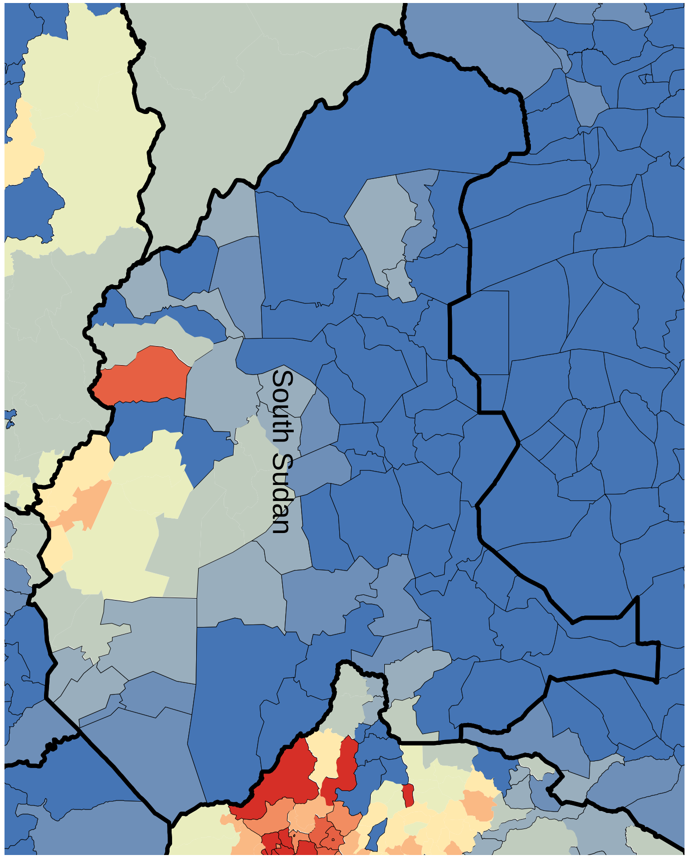 South Sudan

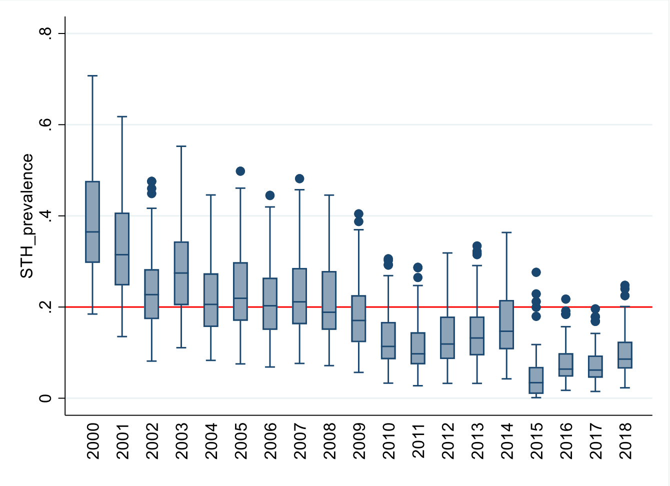
STH_prevalence
0
.2
.4
.6
.8
2000 2001 2002 2003 2004 2005 2006 2007 2008 2009 2010 2011 2012 2013 2014 2015 2016 2017 2018

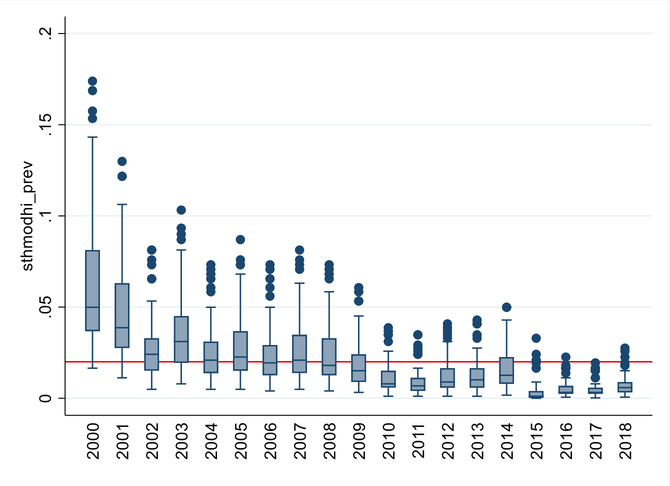
sthmodhi_prev
0
.05
.1
.15
.2
2000 2001 2002 2003 2004 2005 2006 2007 2008 2009 2010 2011 2012 2013 2014 2015 2016 2017 2018

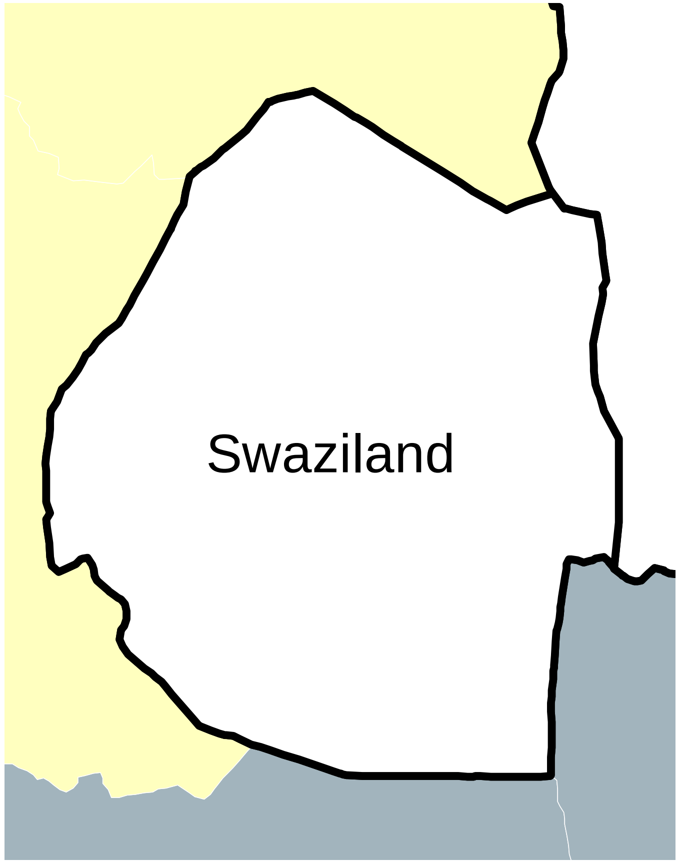
Swaziland

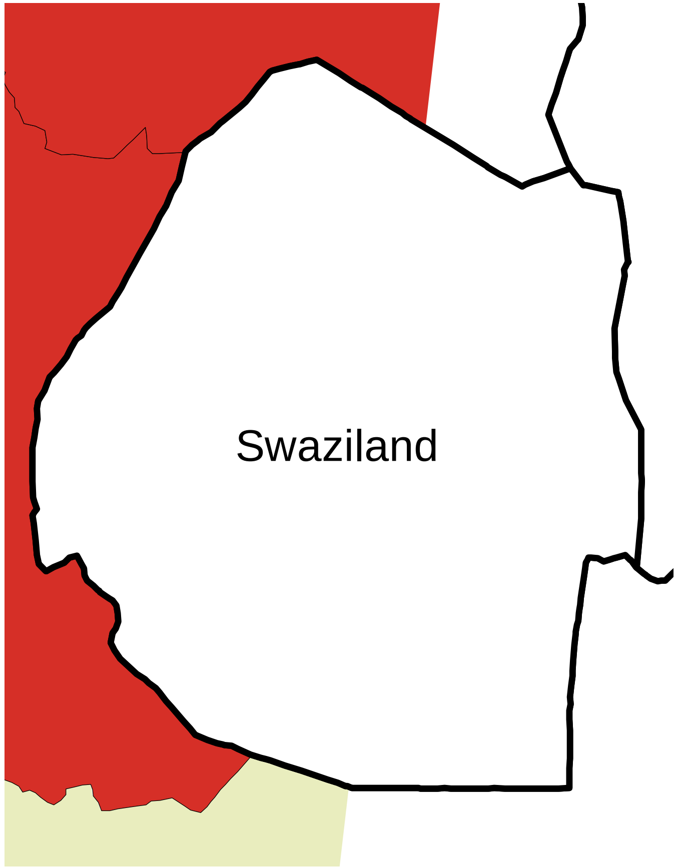
Swaziland

SWZ

TCD

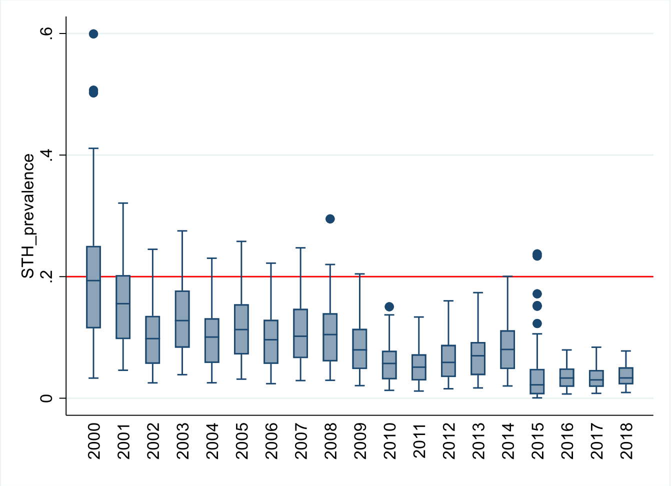
STH_prevalence
0
.2
.4
.6
2000 2001 2002 2003 2004 2005 2006 2007 2008 2009 2010 2011 2012 2013 2014 2015 2016 2017 2018

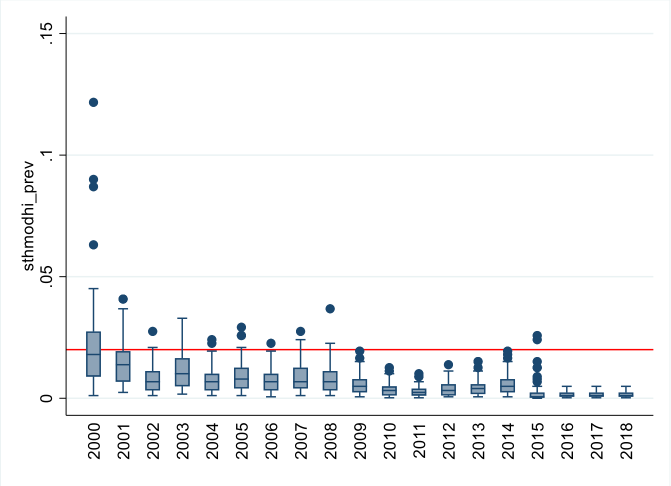
sthmodhi_prev
0
.05
.1
.15
2000 2001 2002 2003 2004 2005 2006 2007 2008 2009 2010 2011 2012 2013 2014 2015 2016 2017 2018

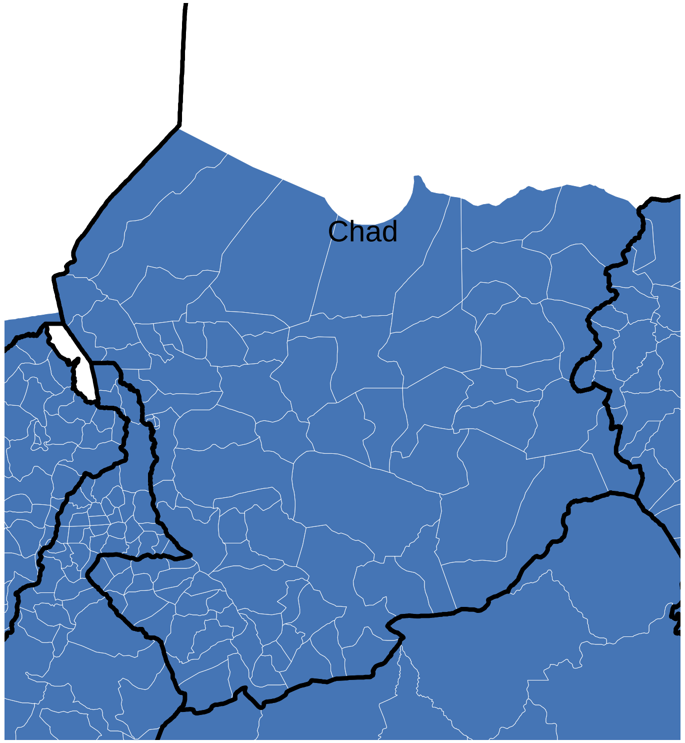
Chad

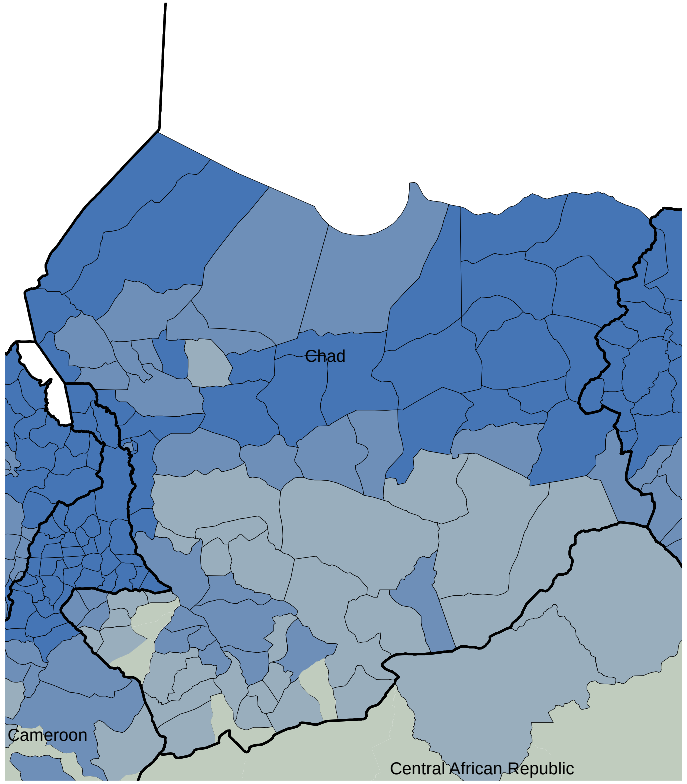
Chad
Cameroon
Central African Republic

TGO	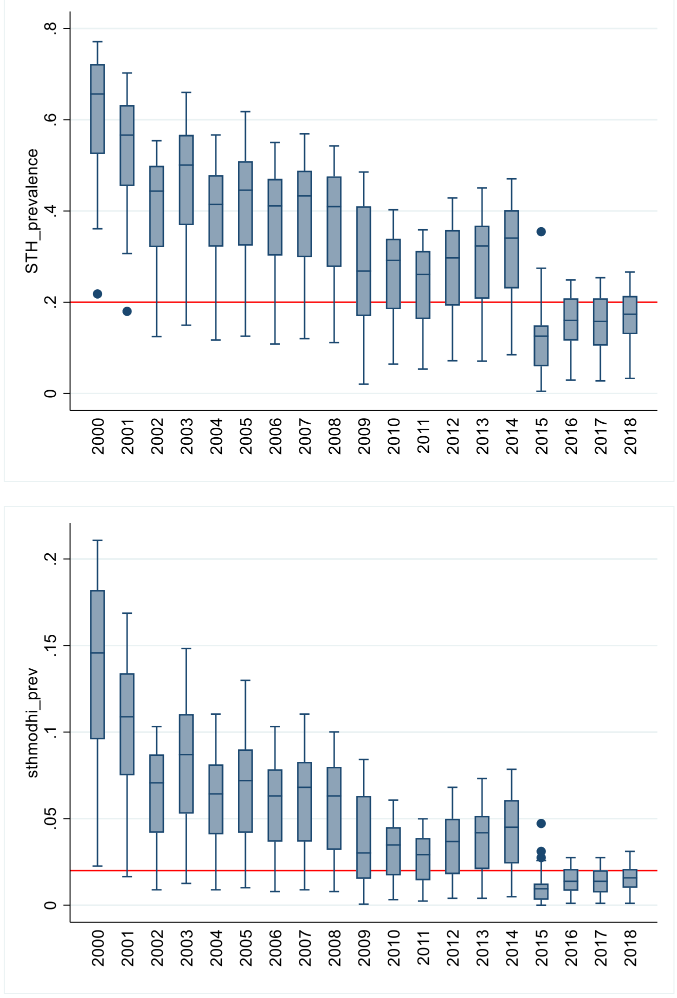STH_prevalence<br>0<br>.2<br>.4<br>.6<br>.8<br>2000<br>2001<br>2002<br>2003<br>2004<br>2005<br>2006<br>2007<br>2008<br>2009<br>2010<br>2011<br>2012<br>2013<br>2014<br>2015<br>2016<br>2017<br>2018<br>sthmodhi_prev<br>0<br>.05<br>.1<br>.15<br>.2	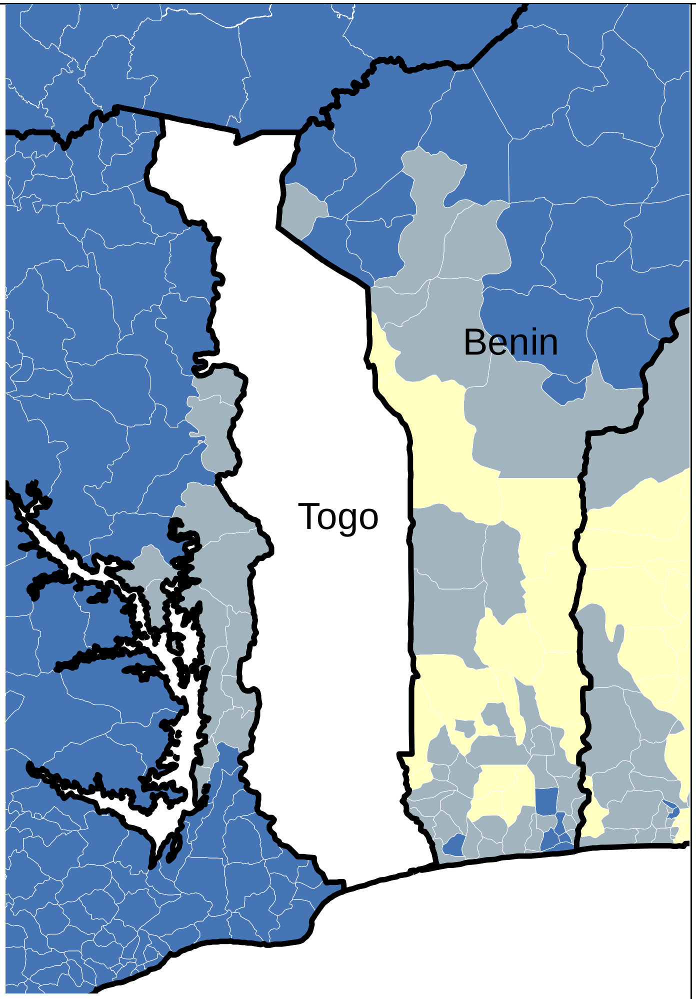Togo<br>Benin	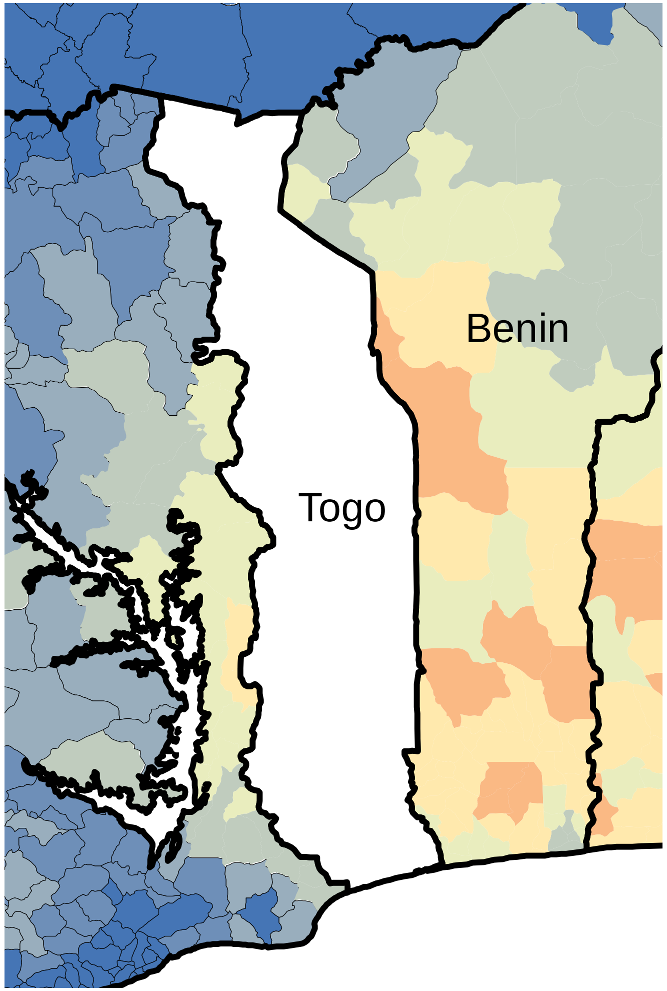Togo<br>Benin

TZA	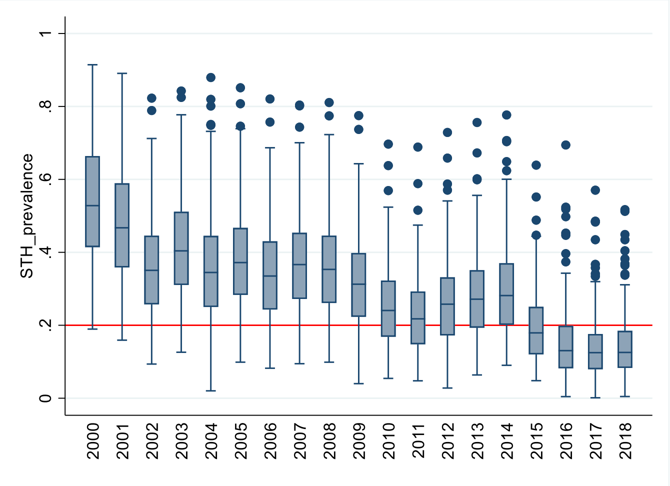 STH_prevalence<br>0<br>.2<br>.4<br>.6<br>.8<br>1<br>2000<br>2001<br>2002<br>2003<br>2004<br>2005<br>2006<br>2007<br>2008<br>2009<br>2010<br>2011<br>2012<br>2013<br>2014<br>2015<br>2016<br>2017<br>2018 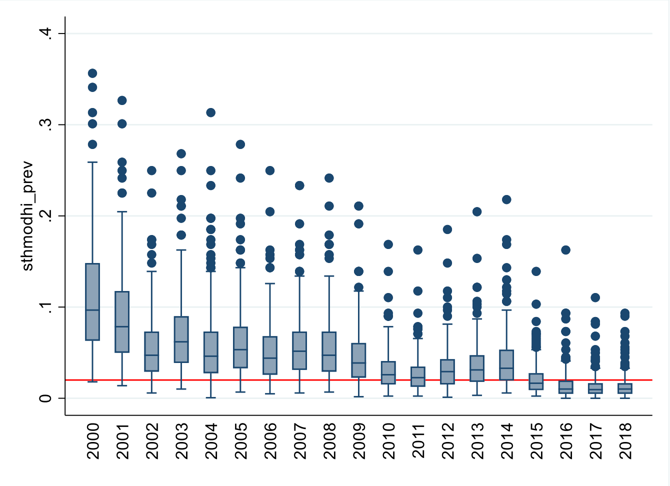 sthmodhi_prev<br>0<br>.1<br>.2<br>.3<br>.4<br>2000<br>2001<br>2002<br>2003<br>2004<br>2005<br>2006<br>2007<br>2008<br>2009<br>2010<br>2011<br>2012<br>2013<br>2014<br>2015<br>2016<br>2017<br>2018	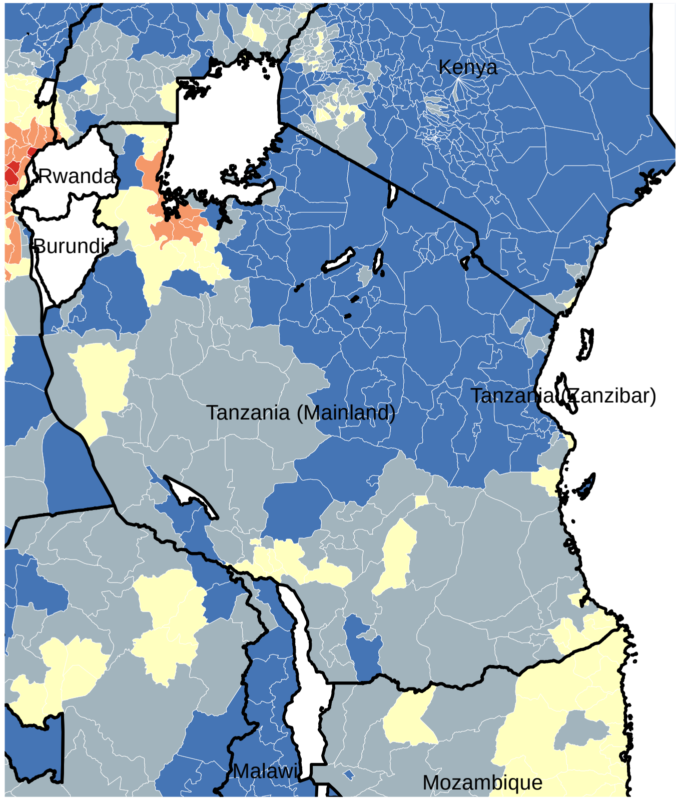 Kenya<br>Rwanda<br>Burundi<br>Tanzania (Mainland)<br>Tanzania (Zanzibar)<br>Malawi<br>Mozambique	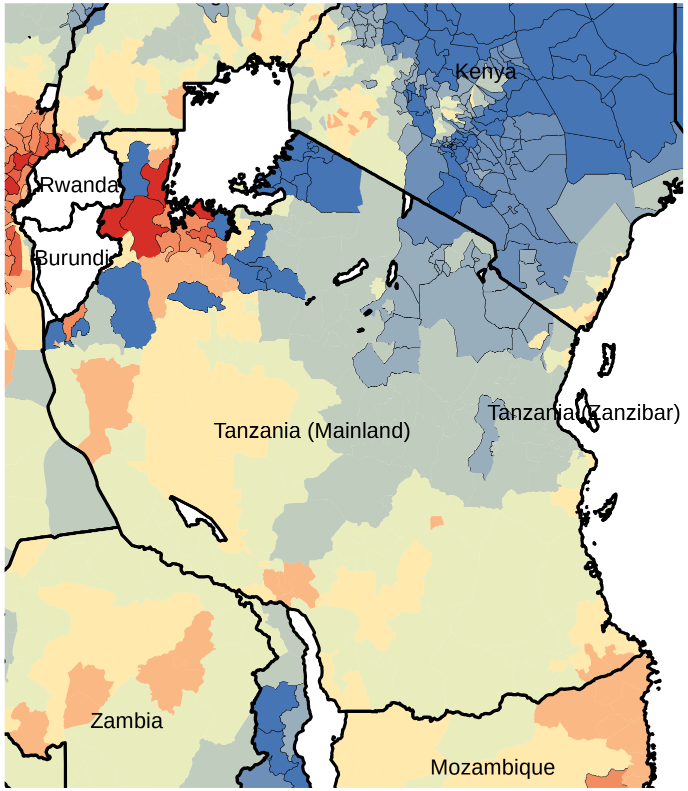 Kenya<br>Rwanda<br>Burundi<br>Tanzania (Mainland)<br>Tanzania (Zanzibar)<br>Zambia<br>Mozambique

TZZ

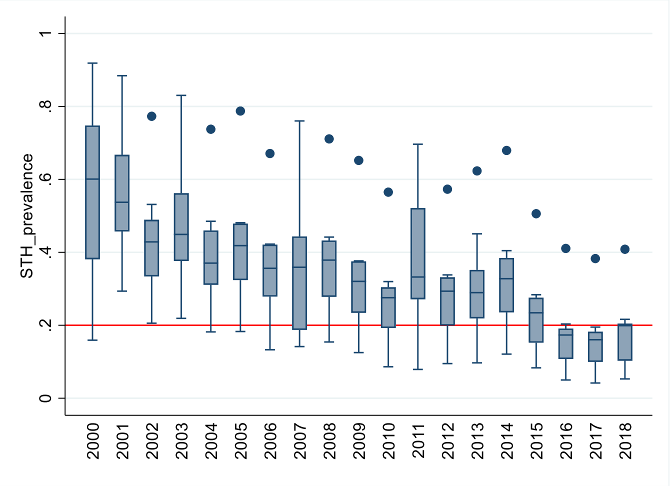
STH_prevalence
0
.2
.4
.6
.8
1
2000 2001 2002 2003 2004 2005 2006 2007 2008 2009 2010 2011 2012 2013 2014 2015 2016 2017 2018

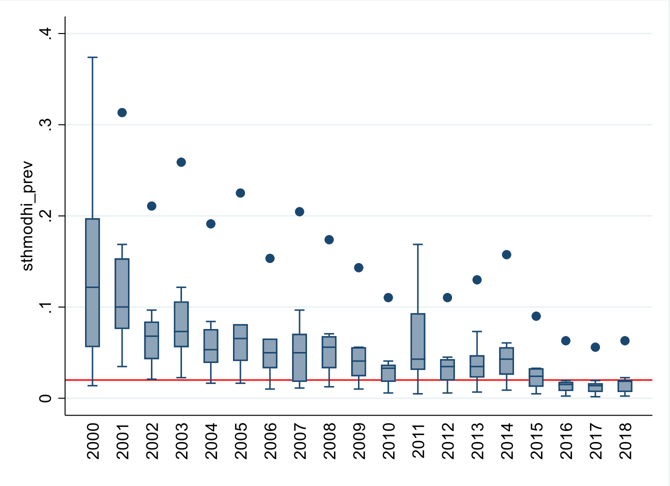
sthmodhi_prev
0
.1
.2
.3
.4
2000 2001 2002 2003 2004 2005 2006 2007 2008 2009 2010 2011 2012 2013 2014 2015 2016 2017 2018

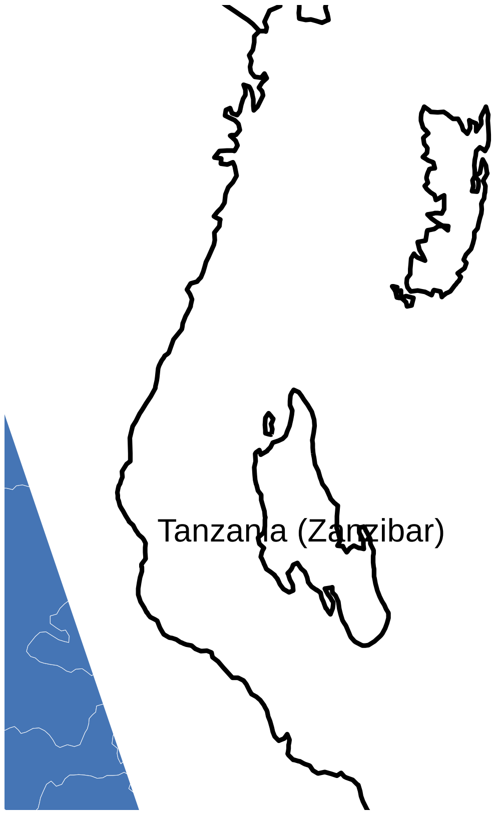
Tanzania (Zanzibar)

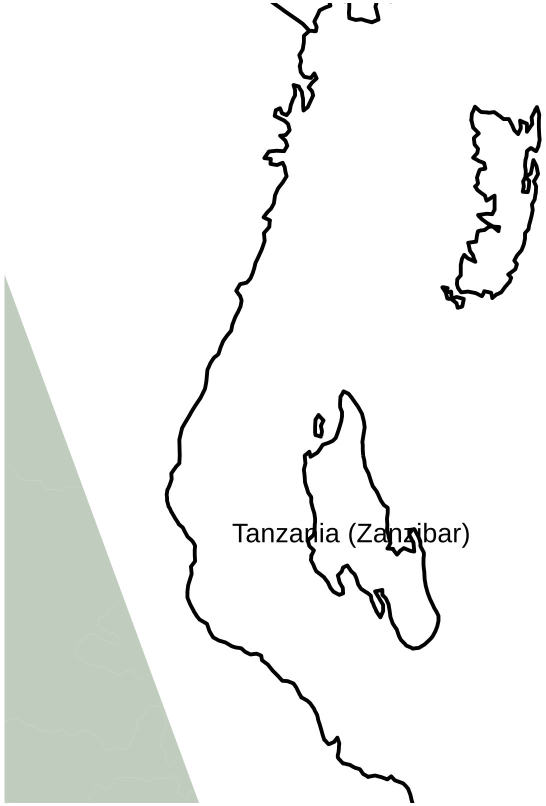
Tanzania (Zanzibar)

UGA	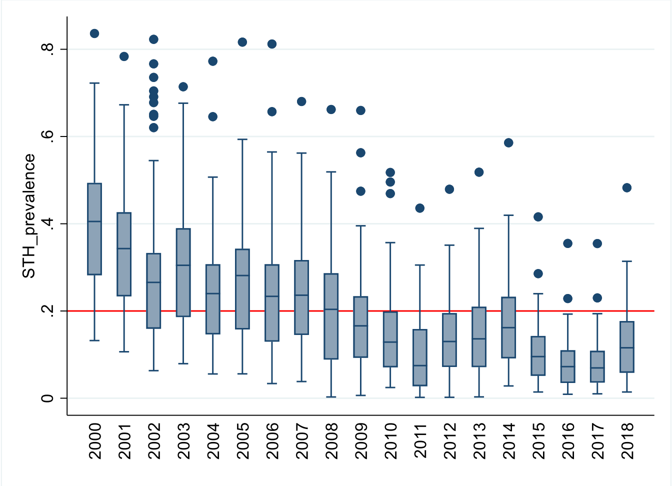<br>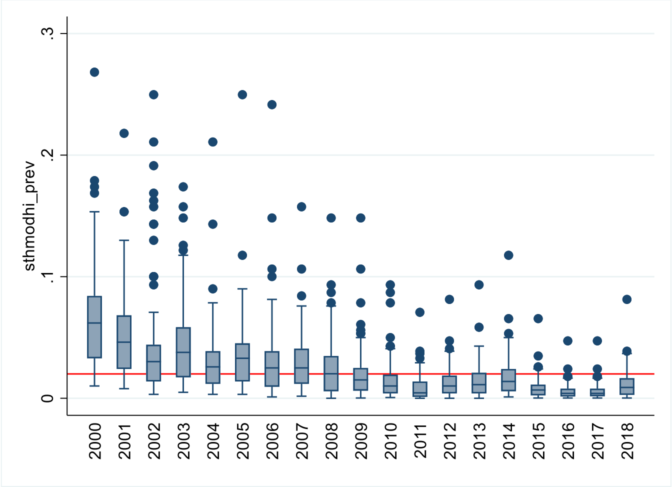	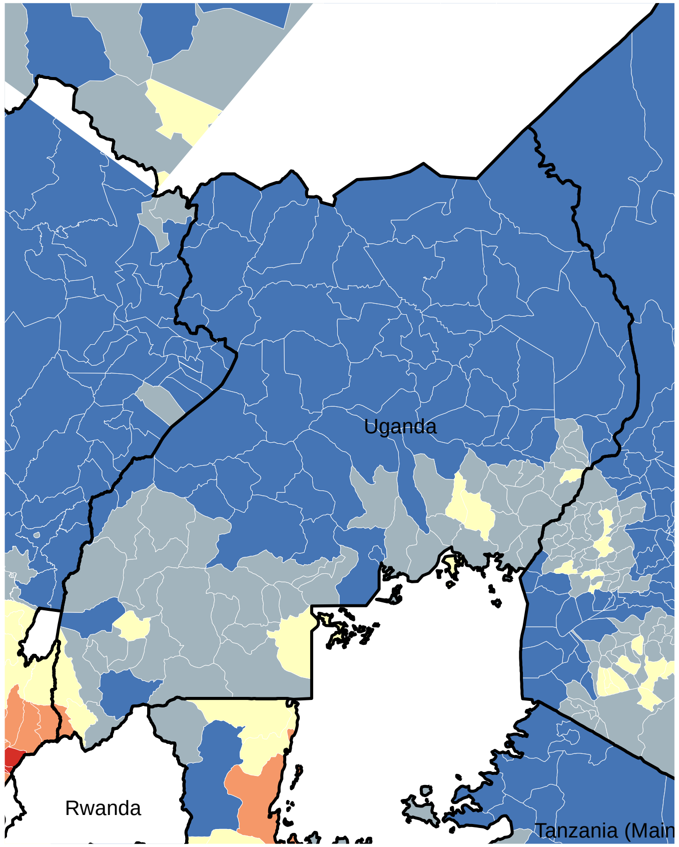	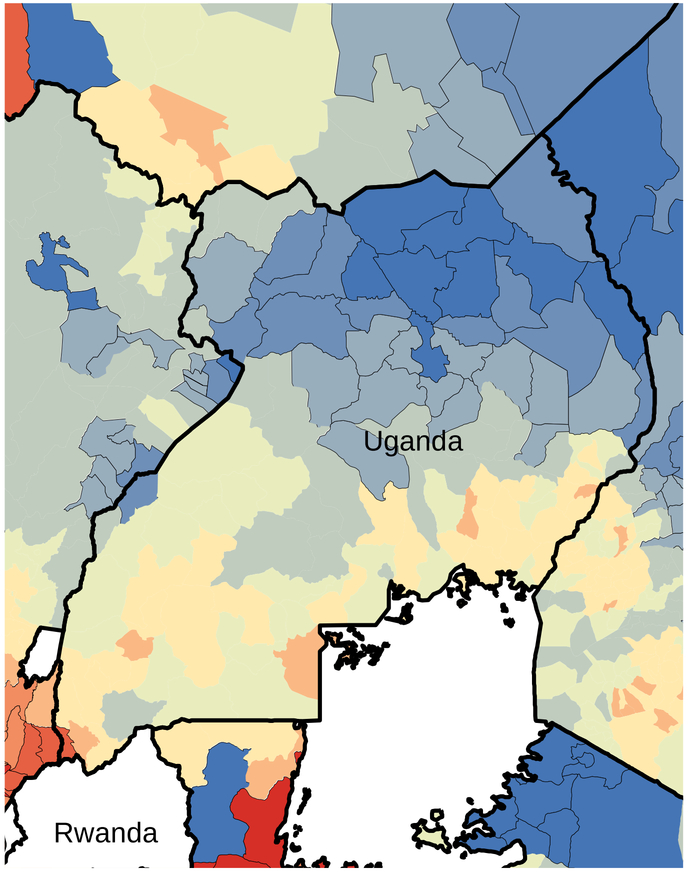

STH_prevalence
0
.2
.4
.6
.8
2000 2001 2002 2003 2004 2005 2006 2007 2008 2009 2010 2011 2012 2013 2014 2015 2016 2017 2018

sthmodhi_prev
0
.1
.2
.3
2000 2001 2002 2003 2004 2005 2006 2007 2008 2009 2010 2011 2012 2013 2014 2015 2016 2017 2018

Uganda
Rwanda
Tanzania (Main

Uganda
Rwanda

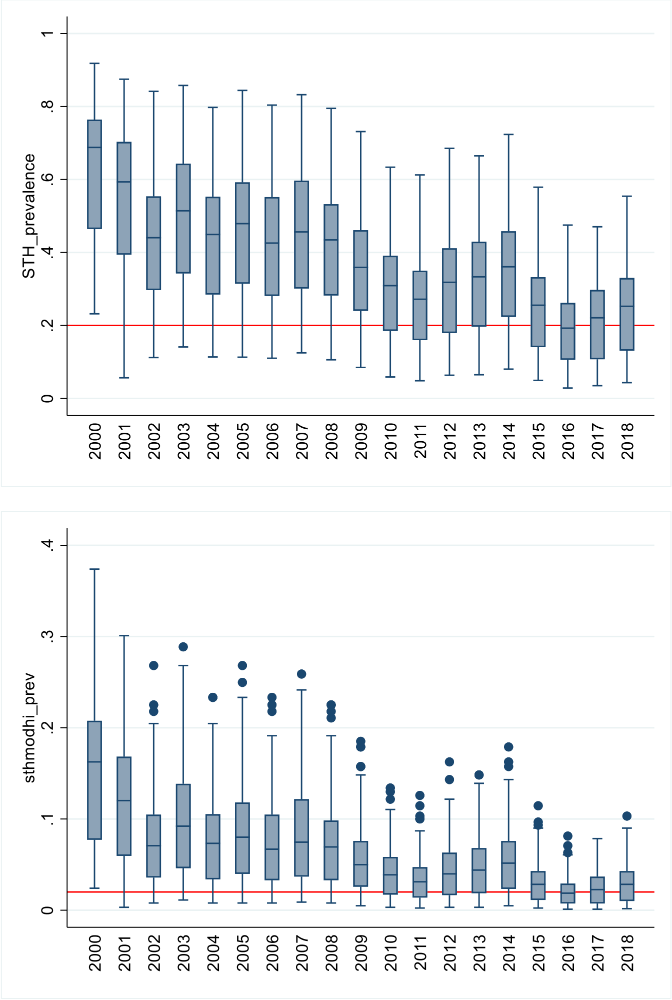
STH_prevalence
0
.2
.4
.6
.8
1
2000 2001 2002 2003 2004 2005 2006 2007 2008 2009 2010 2011 2012 2013 2014 2015 2016 2017 2018
sthmodhi_prev
0
.1
.2
.3
.4
2000 2001 2002 2003 2004 2005 2006 2007 2008 2009 2010 2011 2012 2013 2014 2015 2016 2017 2018

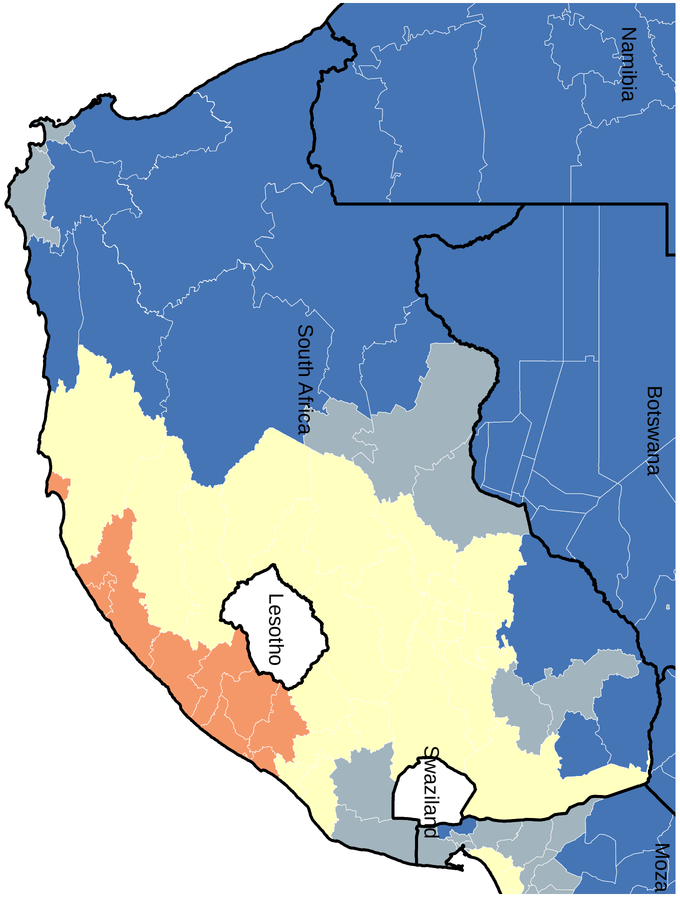
Namibia
South Africa
Botswana
Lesotho
Swaziland

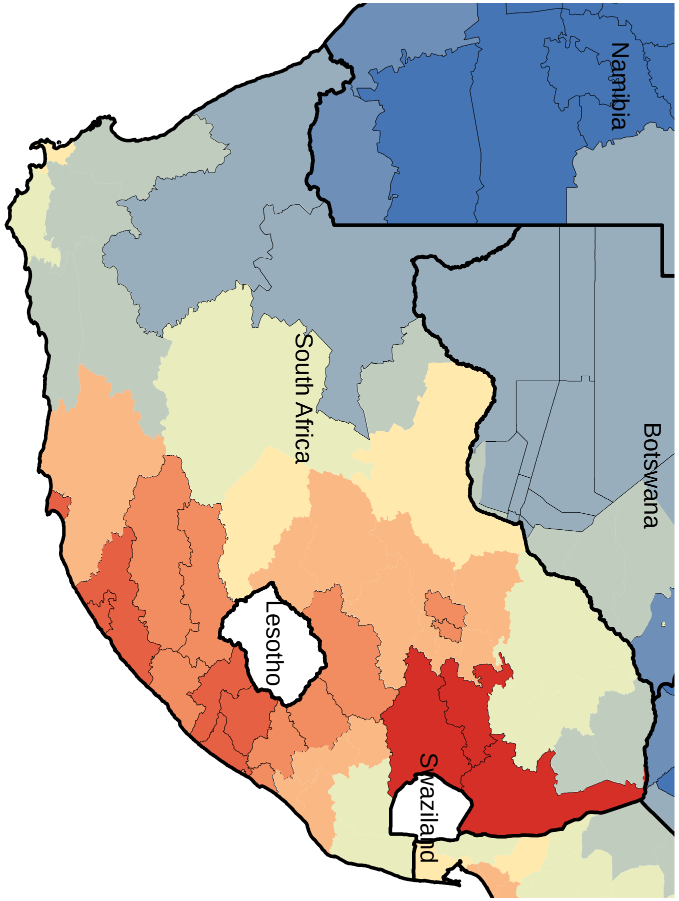
Namibia
South Africa
Botswana
Lesotho
Swaziland

ZAF

ZMB

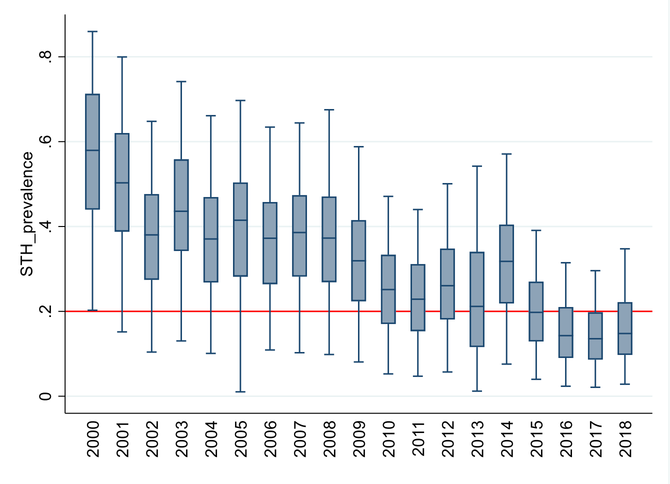
STH_prevalence
0
.2
.4
.6
.8
2000
2001
2002
2003
2004
2005
2006
2007
2008
2009
2010
2011
2012
2013
2014
2015
2016
2017
2018

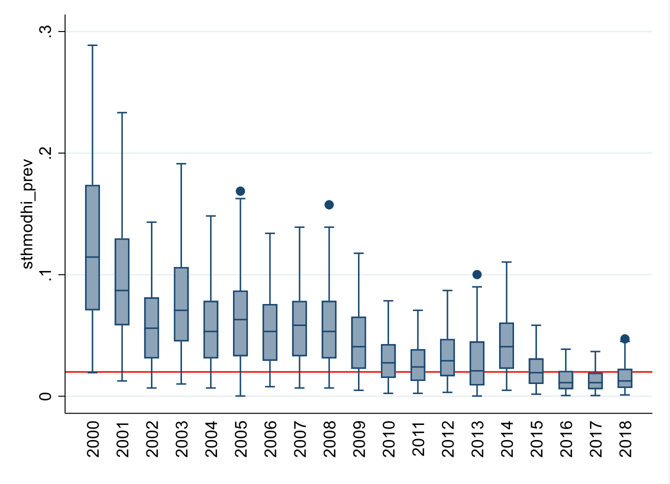
sthmodhi_prev
0
.1
.2
.3
2000
2001
2002
2003
2004
2005
2006
2007
2008
2009
2010
2011
2012
2013
2014
2015
2016
2017
2018

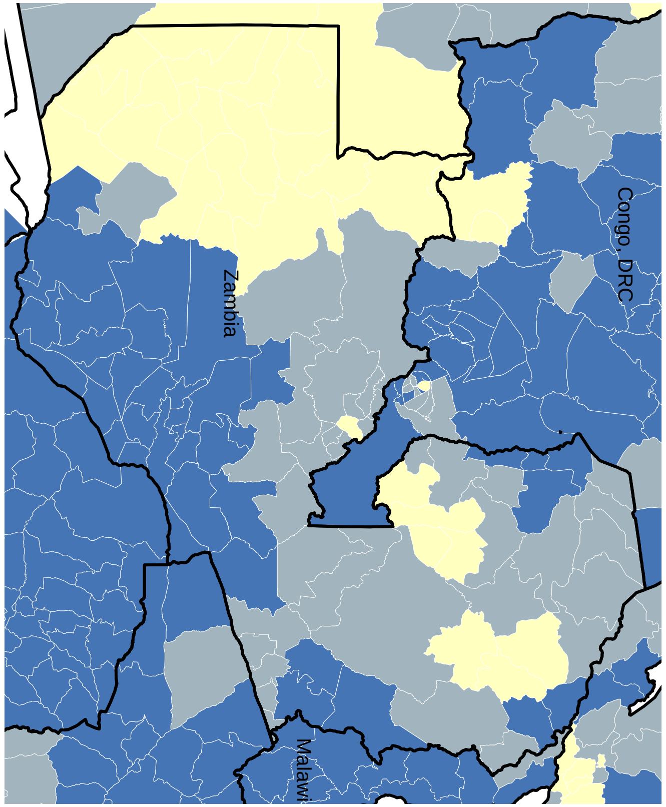
Zambia
Congo, DRC

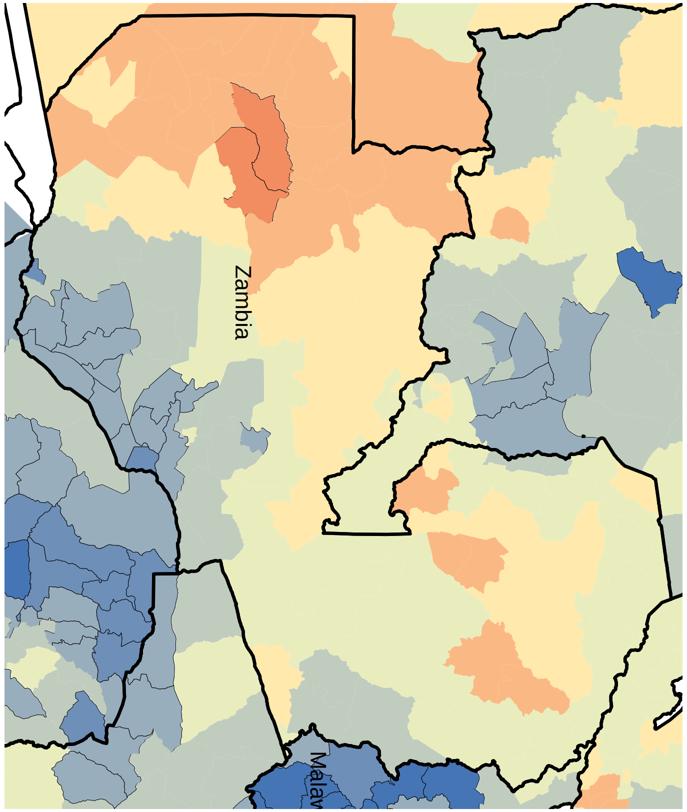
Zambia

ZWE	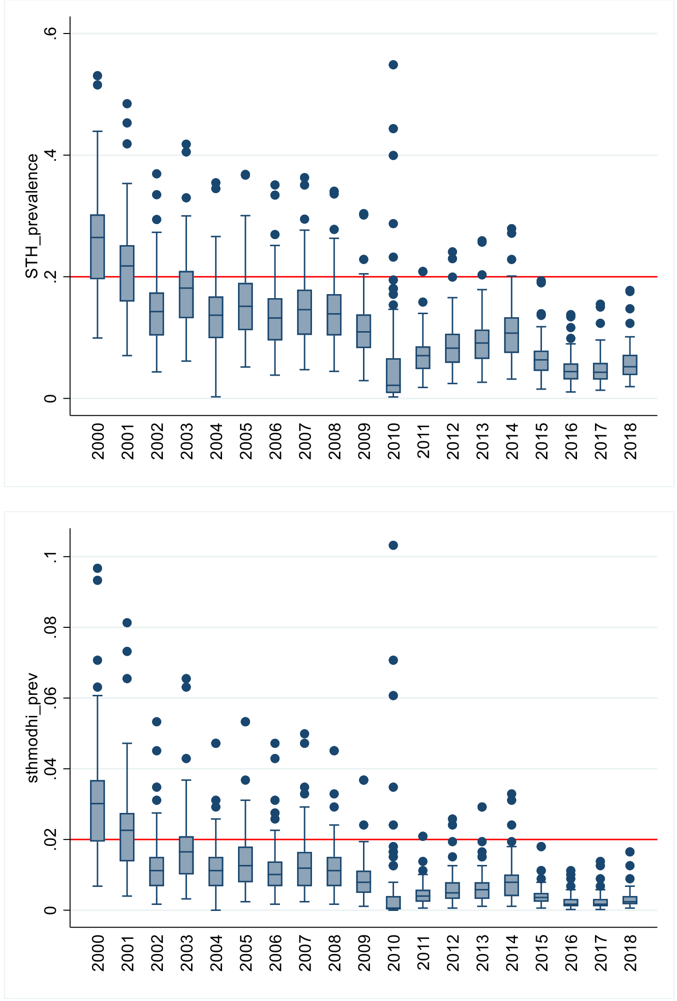STH_prevalence<br>0<br>.2<br>.4<br>.6<br>2000<br>2001<br>2002<br>2003<br>2004<br>2005<br>2006<br>2007<br>2008<br>2009<br>2010<br>2011<br>2012<br>2013<br>2014<br>2015<br>2016<br>2017<br>2018<br>sthmodhi_prev<br>0<br>.02<br>.04<br>.06<br>.08<br>.1	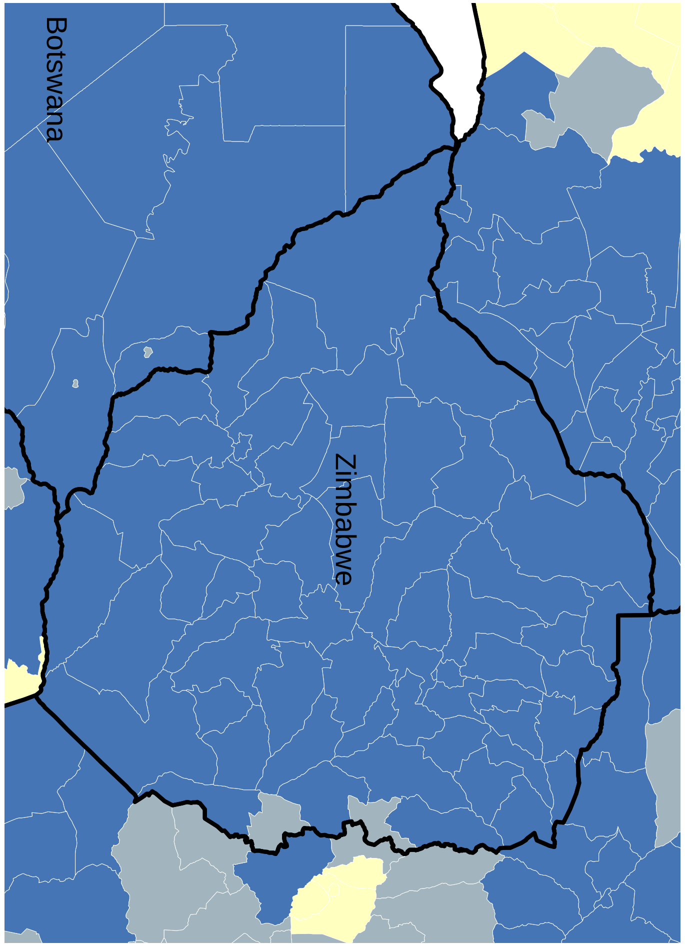Botswana<br>Zimbabwe	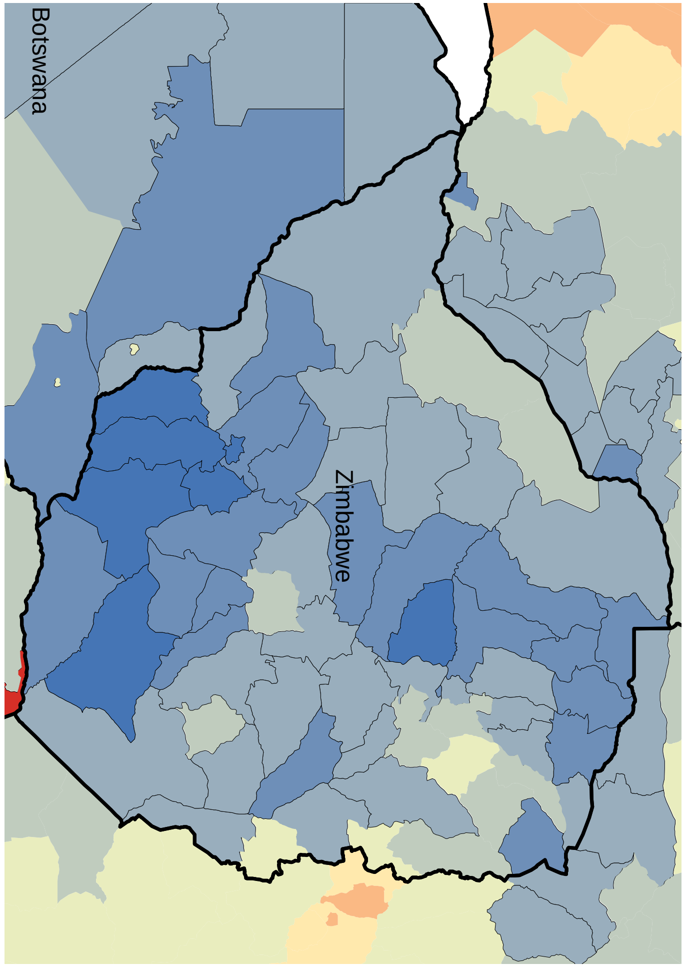Botswana<br>Zimbabwe

ISO3	Full Country Name	ISO3	Full Country Name
AGO	Angola	MLI	Mali
BDI	Burundi	MOZ	Mozambique
BEN	Benin	MRT	Mauritania
BFA	Burkina Faso	MWI	Malawi
BWA	Botswana	NAM	Namibia
CAF	Central African Republic	NER	Niger
CIV	Cote d'Ivoire	NGA	Nigeria
CMR	Cameroon	RWA	Rwanda
COD	Congo, DRC	SDN	Sudan
COG	Congo	SEN	Senegal
DJI	Djibouti	SLE	Sierra Leone
ERI	Eritrea	SOM	Somalia
ETH	Ethiopia	SSD	South Sudan
GAB	Gabon	SWZ	Swaziland
GHA	Ghana	TCD	Chad
GIN	Guinea	TGO	Togo
GMB	The Gambia	TZA	Tanzania (Mainland)
GNB	Guinea-Bissau	TZZ	Tanzania (Zanzibar)
GNQ	Equatorial Guinea	UGA	Uganda
KEN	Kenya	ZAF	South Africa
LBR	Liberia	ZMB	Zambia
LSO	Lesotho	ZWE	Zimbabwe
MDG	Madagascar		

41

Section B: Additional data descriptions, methodological information and results

B1. Gather checklist of information that should be included in new reports of global health estimates

Item #	Checklist item	Reported on page #
Objectives and funding		
1	Define the indicator(s), populations (including age, sex, and geographic entities), and time period(s) for which estimates were made.	6-7
2	List the funding sources for the work.	3
Data Inputs		
For all data inputs from multiple sources that are synthesized as part of the study:		
3	Describe how the data were identified and how the data were accessed.	6-7
4	Specify the inclusion and exclusion criteria. Identify all ad-hoc exclusions.	6-8<br>Supplementary B2, B4
5	Provide information on all included data sources and their main characteristics. For each data source used, report reference information or contact name/institution, population represented, data collection method, year(s) of data collection, sex and age range, diagnostic criteria or measurement method, and sample size, as relevant.	6-7<br>Supplementary B2, B4
6	Identify and describe any categories of input data that have potentially important biases (e.g., based on characteristics listed in item 5).	8<br>Supplementary B6
For data inputs that contribute to the analysis but were not synthesized as part of the study:		
7	Describe and give sources for any other data inputs.	6-7<br>Supplementary B2, B4
For all data inputs:		
8	Provide all data inputs in a file format from which data can be efficiently extracted (e.g., a spreadsheet rather than a PDF), including all relevant meta-data listed in item 5. For any data inputs that cannot be shared because of ethical or legal reasons, such as third-party ownership, provide a contact name or the name of the institution that retains the right to the data.	Supplementary B4
Data analysis		
9	Provide a conceptual overview of the data analysis method. A diagram may be helpful.	7-8<br>Supplementary B10
10	Provide a detailed description of all steps of the analysis, including mathematical formulae. This description should cover, as relevant, data cleaning, data pre-processing, data adjustments and weighting of data sources, and mathematical or statistical model(s).	7-9<br>Supplementary B10
11	Describe how candidate models were evaluated and how the final model(s) were selected.	8-9<br>Supplementary B14
12	Provide the results of an evaluation of model performance, if done, as well as the results of any relevant sensitivity analysis.	9<br>Supplementary B14
13	Describe methods for calculating uncertainty of the estimates. State which sources of uncertainty were, and were not, accounted for in the uncertainty analysis.	8-9

14	State how analytic or statistical source code used to generate estimates can be accessed.	Supplementary B11
Results and Discussion		
15	Provide published estimates in a file format from which data can be efficiently extracted.	
16	Report a quantitative measure of the uncertainty of the estimates (e.g. uncertainty intervals).	10-13<br>Figures 1,2,4
17	Interpret results in light of existing evidence. If updating a previous set of estimates, describe the reasons for changes in estimates.	12-14
18	Discuss limitations of the estimates. Include a discussion of any modelling assumptions or data limitations that affect interpretation of the estimates.	14-15

B2. Distribution and quality of programmatic or survey data points, 1975-2019

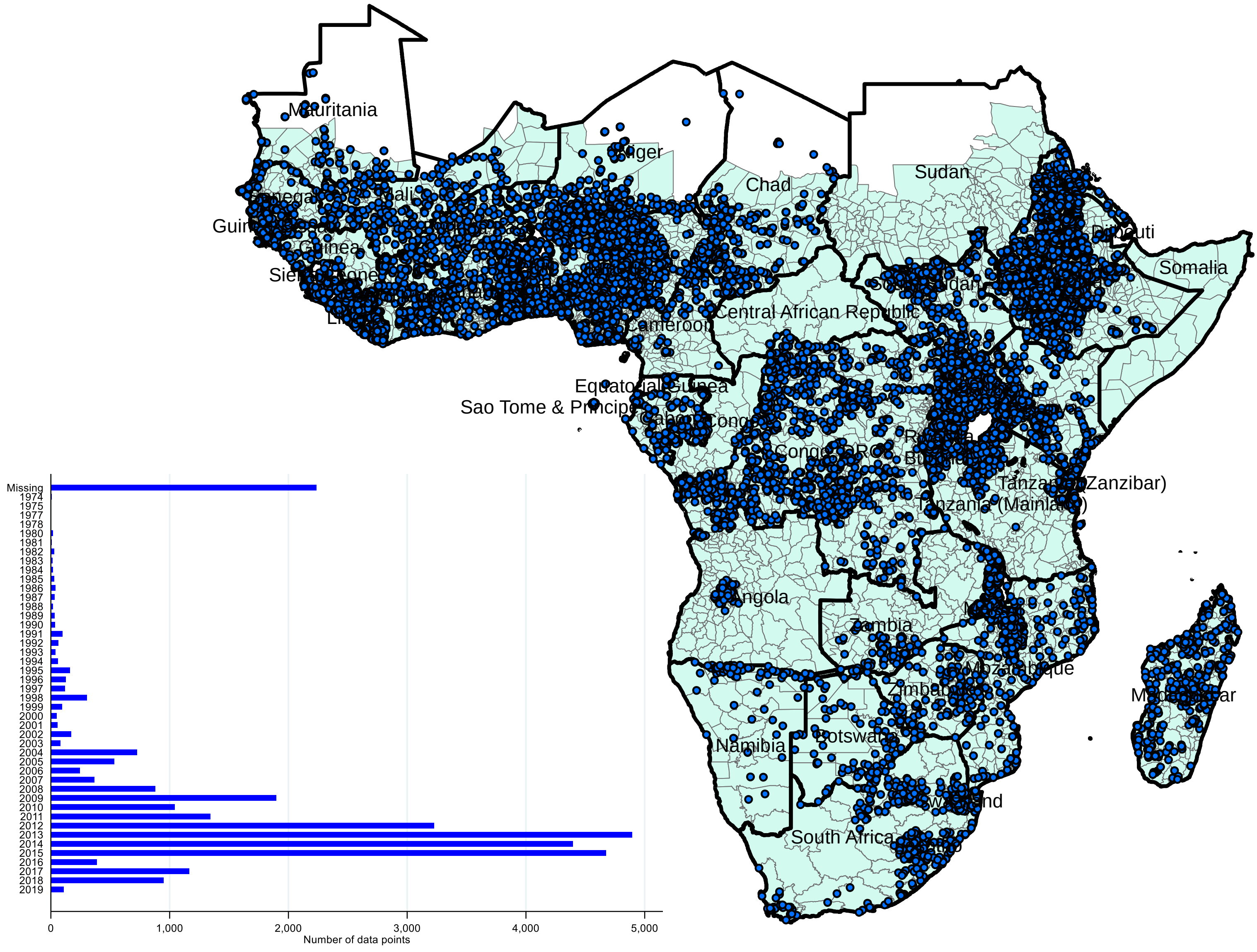

- 30732 Initial input data points or surveys
  - -2236 surveys without year
- 28496
  - -1325 surveys prior to 2000
- 27171
  - -867 surveys which could not be IU located
- 26304 Final input data points or surveys used in analyses

**Georeliability**	**No of data points**	**%**
Reliable: Exact location identified on Google Maps	8,121	30.9%
Reliable: Approx. location identified on Google Maps	237	0.9%
Reliable: Coordinates located within the correct ADM1 boundary	15,814	60.1%
Unreliable: Coordinates not within the correct ADM1 boundary/Not found	2,132	8.1%
Overall	26,304	100.0%

**Grading quality by site from 1998 to 2018**	**No of data points**	**%**
1. Good Quality: Survey conducted since 2005, reporting prevalence of all three species using Kato Katz);	24,496	93.1%
2. Middle Quality: Insufficient information on prevalence, numbers tested or non-standard diagnostic, since 2000;	1,762	6.7%
3. Poor quality: Old data (pre-2000), or insufficient information available on survey details	46	0.2%
Overall	26,304	100.0%

B3: Age profile of survey data points, 2000 to 2018

Age_start	Freq.	Percent	Cum.
0	392	1.49	1.49
1	75	0.29	1.78
2	18	0.07	1.84
3	24	0.09	1.94
4	47	0.18	2.11
**5**	**1,444**	**5.49**	**7.60**
**6**	**1,779**	**6.76**	**14.37**
**7**	**897**	**3.41**	**17.78**
**8**	**1,245**	**4.73**	**22.51**
**9**	**940**	**3.57**	**26.08**
**10**	**4,864**	**18.49**	**44.57**
**11**	**648**	**2.46**	**47.04**
**12**	**352**	**1.34**	**48.38**
**13**	**122**	**0.46**	**48.84**
14	6	0.02	48.86
15	3	0.01	48.87
16	1	0.00	48.88
17	1	0.00	48.88
18	6	0.02	48.91
20	2	0.01	48.91
21	1	0.00	48.92
22	1	0.00	48.92
23	1	0.00	48.92
26	1	0.00	48.93
27	1	0.00	48.93
28	3	0.01	48.94
29	1	0.00	48.95
32	1	0.00	48.95
33	1	0.00	48.95
35	1	0.00	48.96
40	1	0.00	48.96
**.**	**13,425**	**51.04**	**100.00**
Total	26,304	100.00	

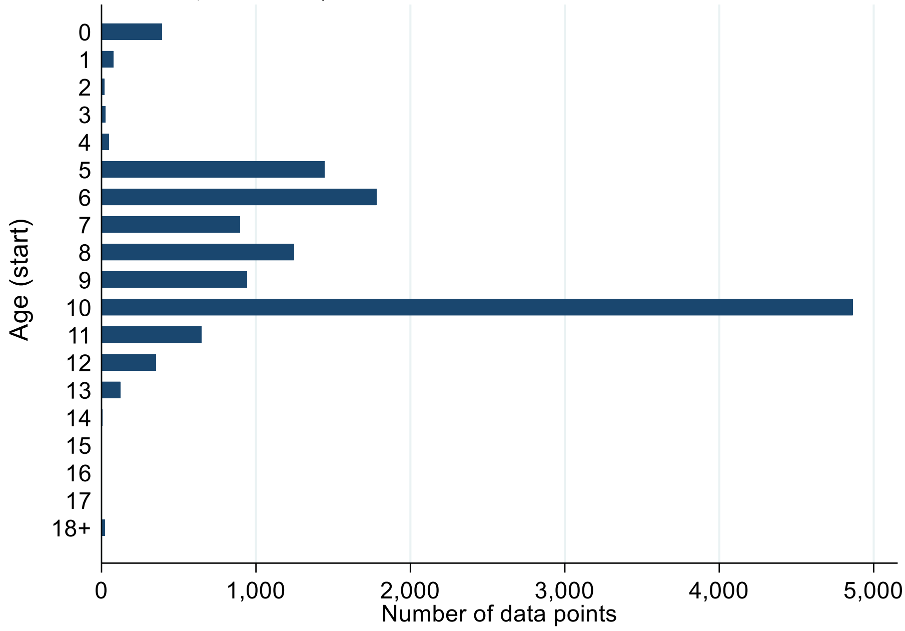

```
. rename ageend2 AgeEnd

. tab AgeStart AgeEnd , missing nowrap
```

AgeStart	AgeEnd 0	4	5	6	8	9	10	11	12	13	14	15	16	17	18	19	.	Total
0	249	0	5	4	0	67	2	0	2	2	5	1	2	2	3	48	0	392
1	3	1	1	0	0	0	2	0	3	2	15	1	0	8	0	39	0	75
2	0	0	0	0	1	0	0	0	0	4	4	0	1	0	1	7	0	18
3	0	0	0	0	0	0	0	1	0	1	5	11	0	0	2	4	0	24
4	0	0	0	0	0	0	4	3	4	6	10	9	3	3	1	4	0	47
5	6	0	0	0	0	1	13	9	23	41	153	225	446	15	193	319	0	1,444
6	8	0	0	0	2	1,111	26	74	59	67	169	106	61	33	32	31	0	1,779
7	16	0	0	0	9	7	98	78	127	194	232	57	30	29	5	15	0	897
8	21	0	0	0	0	0	77	15	103	125	531	71	74	12	196	20	0	1,245
9	22	0	0	0	0	2	26	9	298	87	294	144	26	6	12	14	0	940
10	220	0	0	0	0	0	6	16	138	499	3,244	574	84	27	21	35	0	4,864
11	7	0	0	0	0	0	0	7	18	54	286	204	26	12	12	22	0	648
12	13	0	0	0	0	0	0	0	14	28	121	91	44	16	10	15	0	352
13	0	0	0	0	0	0	0	0	0	5	92	11	4	3	2	5	0	122
14	0	0	0	0	0	0	0	0	0	0	1	0	0	2	0	3	0	6
15	0	0	0	0	0	0	0	0	0	0	0	0	1	0	0	2	0	3
16	0	0	0	0	0	0	0	0	0	0	0	0	0	0	0	1	0	1
17	0	0	0	0	0	0	0	0	0	0	0	0	0	0	0	1	0	1
18	0	0	0	0	0	0	0	0	0	0	0	0	0	0	0	20	1	21
.	0	0	0	0	0	0	0	0	0	0	0	0	0	0	0	0	13,425	13,425
Total	565	1	6	4	12	1,188	254	212	789	1,115	5,162	1,505	802	168	490	605	13,426	26,304

```
. tab Year missing_age
```

Year	missing_age 0	1	Total
2000	18	28	46
2001	39	12	51
2002	86	45	131
2003	58	10	68
2004	131	590	721
2005	226	201	427
2006	79	160	239
2007	47	304	351
2008	358	503	861
2009	350	1,532	1,882
2010	81	956	1,037
2011	114	1,217	1,331
2012	1,308	1,909	3,217
2013	2,517	2,343	4,860
2014	2,943	1,337	4,280
2015	2,539	1,914	4,453
2016	138	103	241
2017	1,102	0	1,102
2018	745	261	1,006
Total	12,879	13,425	26,304

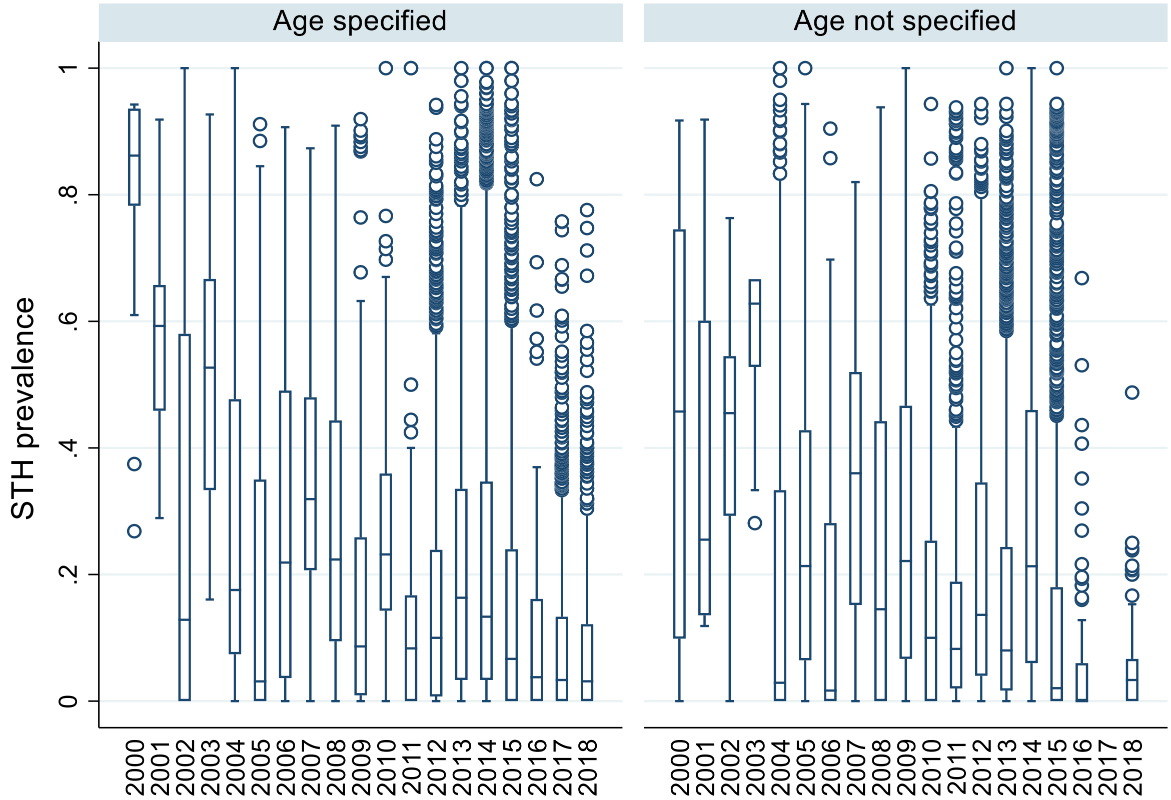


```
. xtmixed Cum_Prevalence missing_age || ISO3: || idlim2:, vce(robust)

Mixed-effects regression                        Number of obs     =     26,304

-------------------------------------------------------------
                |     No. of       Observations per Group
 Group Variable |     Groups    Minimum    Average    Maximum
----------------+--------------------------------------------
           ISO3 |         46          1      571.6      3,045
         idlim2 |      3,594          1        7.3        411
-------------------------------------------------------------

                                                Wald chi2(1)      =       1.01
Log pseudolikelihood =  8315.5478               Prob > chi2       =     0.3152

                                   (Std. Err. adjusted for 46 clusters in ISO3)
--------------------------------------------------------------------------------
               |               Robust
Cum_Prevalence |      Coef.   Std. Err.      z    P>|z|     [95% Conf. Interval]
---------------+----------------------------------------------------------------
   missing_age |   .0438275   .0436352     1.00   0.315    -.0416959    .1293508
         _cons |   .1840767   .0344213     5.35   0.000     .1166122    .2515413
--------------------------------------------------------------------------------

------------------------------------------------------------------------------
                             |               Robust
  Random-effects Parameters  |   Estimate   Std. Err.     [95% Conf. Interval]
-----------------------------+------------------------------------------------
ISO3: Identity               |
                   sd(_cons) |    .154399   .0162615      .1256016    .1897991
-----------------------------+------------------------------------------------
idlim2: Identity             |
                   sd(_cons) |    .144734    .009942      .1265029    .1655925
-----------------------------+------------------------------------------------
                sd(Residual) |   .1577436   .0105519      .1383607    .1798419
------------------------------------------------------------------------------
```

```
. logit Cum_Prevalence missing_age, cluster(idlim2)

Logistic regression                             Number of obs     =     26,304
                                                Wald chi2(1)      =       0.28
                                                Prob > chi2       =     0.5994
Log pseudolikelihood = -14655.754               Pseudo R2         =     0.0001

                              (Std. Err. adjusted for 3,545 clusters in idlim2)
--------------------------------------------------------------------------------
               |               Robust
Cum_Prevalence |      Coef.   Std. Err.      z    P>|z|     [95% Conf. Interval]
---------------+----------------------------------------------------------------
   missing_age |  -.0388108   .0738784    -0.53   0.599    -.1836099    .1059883
         _cons |   1.142296   .0712945    16.02   0.000     1.002561     1.28203
--------------------------------------------------------------------------------
```

B4. Detailed description of study covariates

A detailed summary of the covariates, source as well as temporal and spatial resolution can be found in the table below.

Climatic data by year for 2000 to 2017 were derived using high-resolution satellite and meteorological data from the WorldClim database at 1 km spatial resolution(1). Aridity was calculated using mean annual precipitation divided by mean annual Potential Evapo-Transpiration (PET). Soil porosity, using sand fraction of the top-soil as a proxy, and top-soil pH data were extracted from SoilGrids system at a 250 m resolution available at https://soilgrids.org/ with a published description(2).

Data for living in a house that has dirt or earth floors and living in slum conditions were extracted from high-resolution, standardized estimates of housing conditions across SSA for 2000 to 2015 recently published (3) and available at https://map.ox.ac.uk/research-project/housing_in_africa/. As a further marker for poverty, we utilised annual gridded dataset predictions for GDP at purchasing power parity (GDP PPP) at 5 arc-min resolution available for 1990 to 2015 (4). Treatment data at the IU level were provided by national programmes to ESPEN as part of routine reporting. Only LF MDA rounds that exceeded 65% population coverage were included.

Environmental, climatic and socio-economic/development raster data were imported into ArcGIS 10.5 (ESRI 2011. ArcGIS Desktop: Release 10. Redlands, CA: Environmental Systems Research Institute) and linked by geographical location (IU) and year (if TV) to the parasitological survey data. For IUs without parasitological data points we took the median value of a given covariate (and by year if TV) as the value for inclusion in the model. For IUs with parasitological cluster data (with latitude/longitude) the value of the covariate at the location (or closest to it) was utilised.

Table B4: Summary of explanatory covariates tested and/or utilised in the model:

Grouping	Variable	Source	Temporal resolution	Spatial resolution
Climatic/terrain	Max temperature	WorldClim (1, 5)	Monthly, 2000 to 2016	1km
	Aridity index (ratio of the annual precipitation and potential evapotranspiration (PET) totals)	WorldClim (1)	Period, 1970-2000	~1km
	Soil type (texture fraction)	SoilGrids250m (2)	Updated 2016, static	250m
	Soil pH	SoilGrids250m (2)	Updated 2016, static	250m
Socio-economic/living conditions	Lack of access to safe drinking water	Local Burden of Disease (LBD) project (The Lancet Global Health, In press)	Annual, 1990-2017	5km
	Lack of access to sanitation facilities	Local Burden of Disease (LBD) project (The Lancet Global Health, In press)	Annual, 1990-2017	5km
	Night lights	Earth observatory (NASA) (https://earthobservatory.nasa.gov/features/NightLights)	Annual, 2012, 2016	3km
	GDP at purchasing power parity (GDP PPP)	Gridded global datasets for Gross Domestic Product and Human Development Index (4)	Annual, 1990-2015	5arc-minutes or ~ 10 km at

				the equator
	Population count (overall and school aged between 5 to 14 years)	Gridded Population of the World (GPW), version 4, SEDAC https://sedac.ciesin.columbia.edu/data/collection/gpw-v4	Annual 2000-2017	1km
	Slum to non-slum living conditions	Mapping changes in housing in sub-Saharan Africa from 2000 to 2015 (3)	Annual, 2000-2015	5x5km
Programmatic	Cumulative number of effective mass drug administrations for LF (>75% coverage) and school based deworming for STH	ESPEN http://espen.afro.who.int/	Annual, 1990-2018	IU level

B5.1 Fitted relationship between prevalence of infection for a given STH subtype (red= *Ascaris lumbricoides*, blue=hookworm, grey= *Trichuris trichiura*) and intensity of infection (moderate to heavy) prior to mass drug administration.

Note: dashed lines provide uncertainty intervals for these estimates.

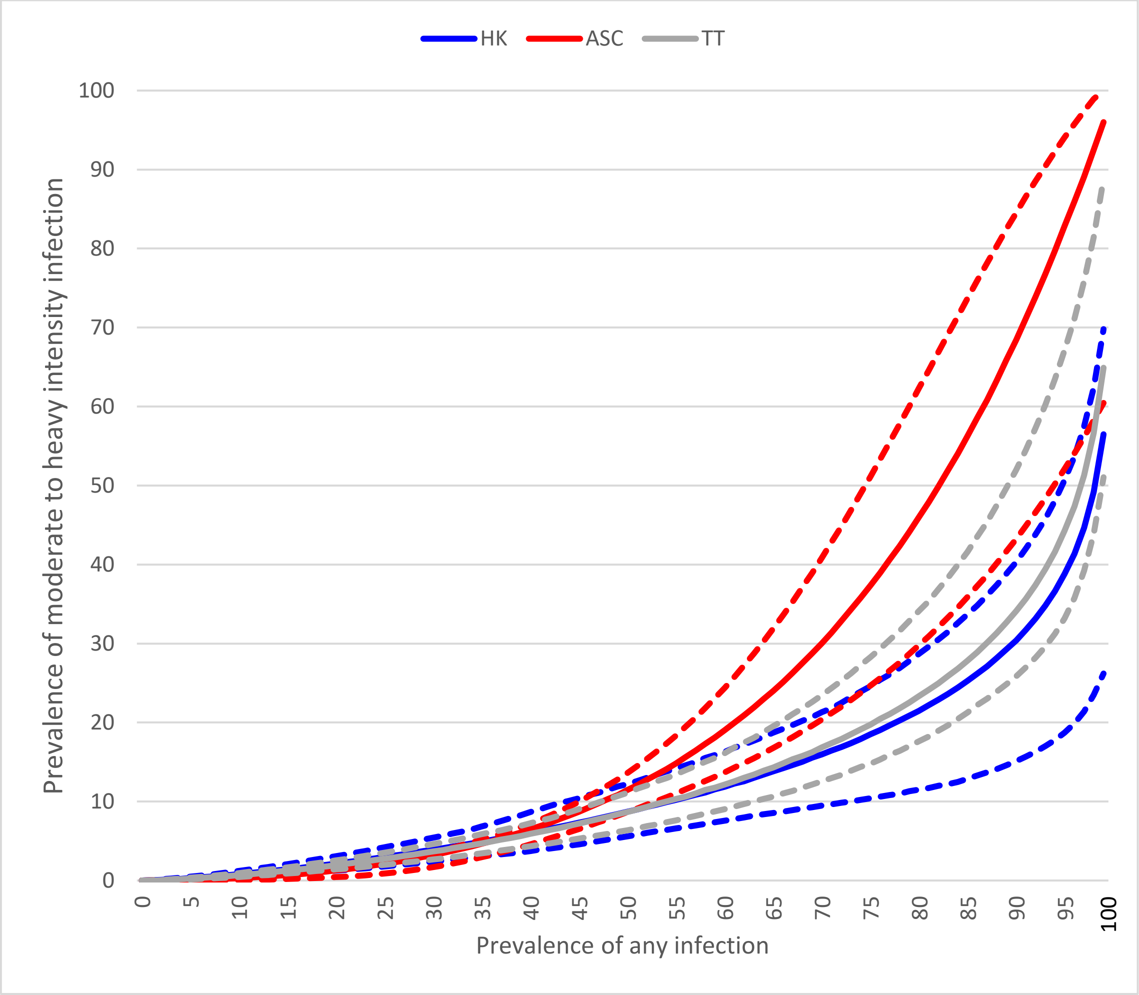

B5.2 Validation of fitted relationship between intensity and prevalence compared to independent data from the Tumikia cluster randomised trial in Kenya [1,2], both at baseline (circles) and post (squares) PC treatment. Note relationship for *Ascaris lumbricoides* not displayed due to small sample size of moderate-to-heavy intensity infections in trial setting.

Hookworm *Trichuris trichiura*

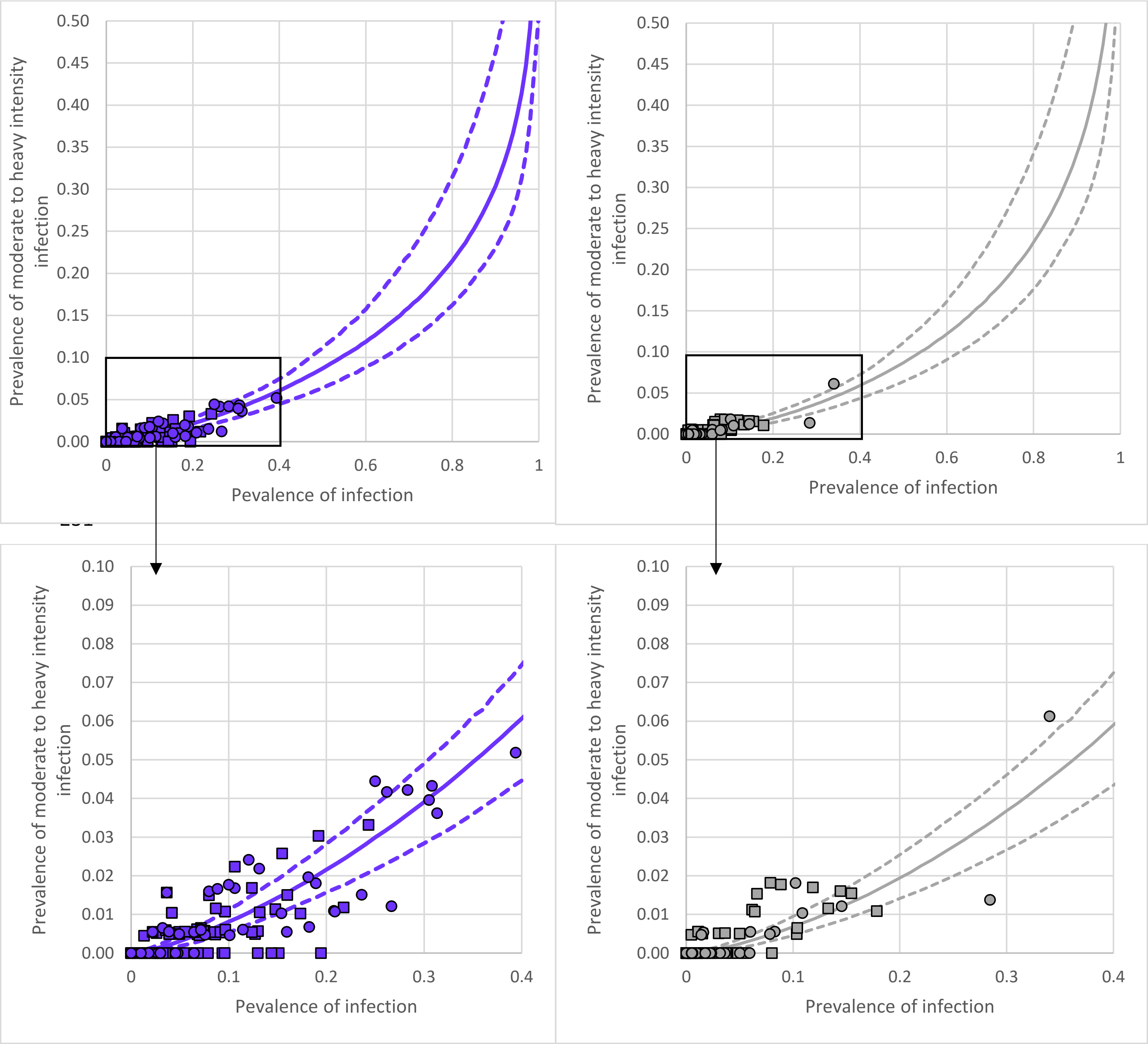

1. Brooker SJ, Mwandawiro CS, Halliday KE, Njenga SM, McHaro C, Gichuki PM, et al. Interrupting transmission of soil-transmitted helminths: a study protocol for cluster randomised trials evaluating alternative treatment strategies and delivery systems in Kenya. BMJ open. 2015;5(10):e008950. pmid:26482774
2. Halliday KE, Oswald WE, Mcharo C, Beaumont E, Gichuki PM, Kepha S, et al. (2019) Community-level epidemiology of soil-transmitted helminths in the context of school-based deworming: Baseline results of a cluster randomised trial on the coast of Kenya. PLoS Negl Trop Dis 13(8): e0007427. https://doi.org/10.1371/journal.pntd.0007427

B6: Relationships between STH prevalence and selected covariates plus outlier identification (in red)

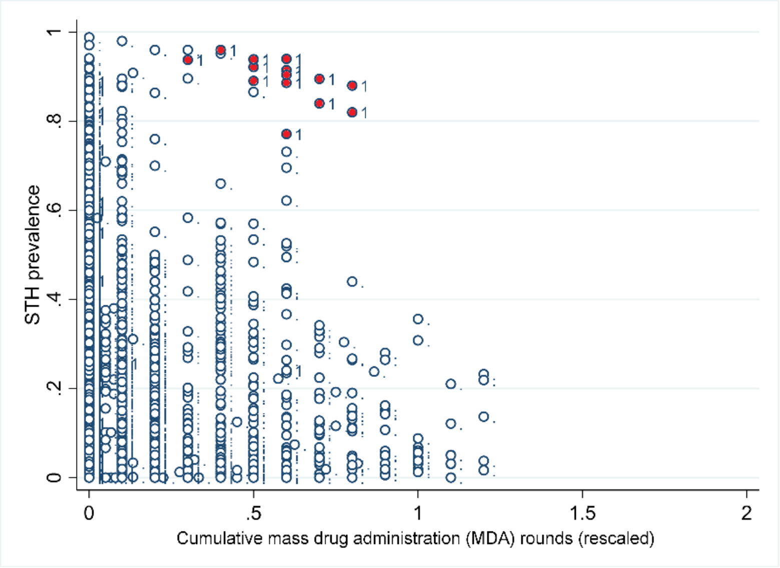

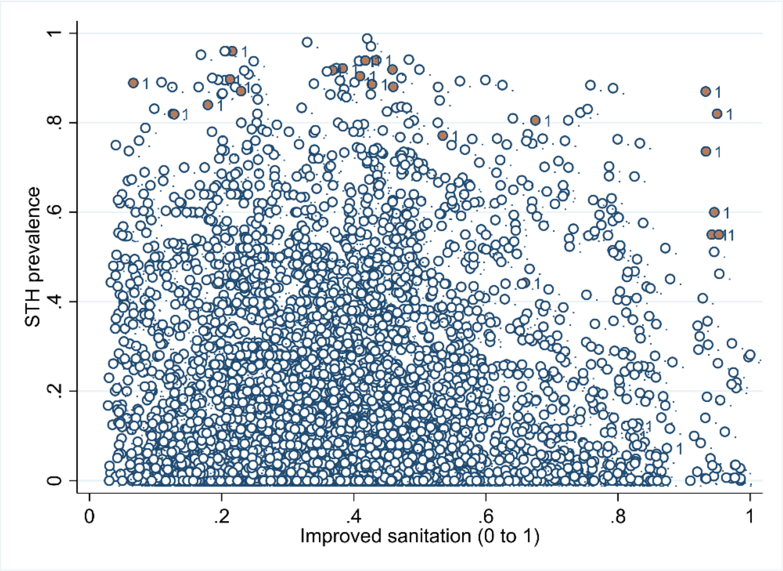

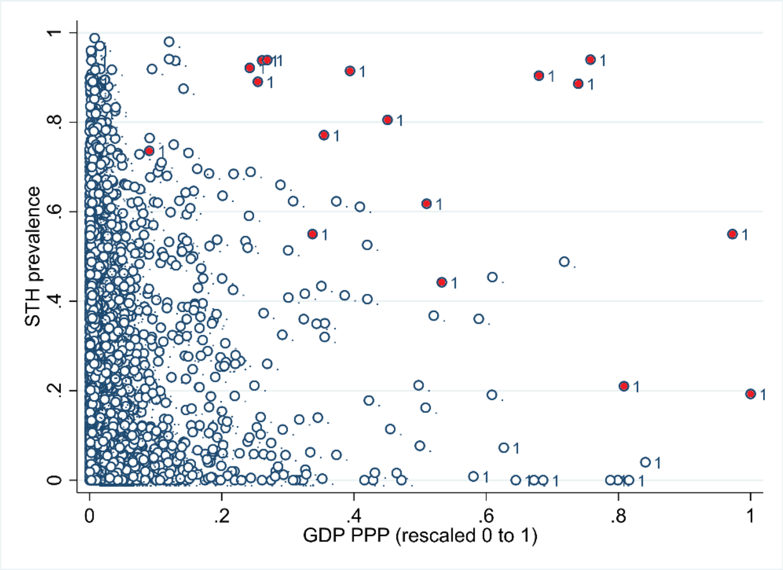

B7. a) Summary of data points by country for the period 2000-2018 and b) comparison to resultant Bayesian credibility interval uncertainty

**Country**	**Data points (2000-2018)**	**Total cumulative sample size (2000-2018)**
**Central African Republic**	**0**	**0**
**Congo**	**0**	**0**
**Djibouti**	**0**	**0**
**Equatorial Guinea**	**0**	**0**
**Somalia**	**0**	**0**
**Sudan**	**1**	**0**
Seychelles	6	201
Cape Verde	16	1661
Guinea-Bissau	36	1279
Mauritius	49	1495
Lesotho	50	2517
Sao Tome & Principe	75	2551
Namibia	80	4833
Guinea	85	4249
Ghana	90	743
Burkina Faso	113	8666
Sierra Leone	114	6730
Senegal	135	1356
Mauritania	140	12791
Angola	160	3585
Botswana	161	7302
Mozambique	162	7900
South Africa	211	8494
Mali	221	17480
Swaziland	276	13834
Gabon	280	13513
Zimbabwe	283	12368
Cameroon	298	13192
Madagascar	353	18385
Eritrea	368	17462
Chad	409	20469
The Gambia	431	21194
South Sudan	434	18810
Rwanda	445	16778
Benin	448	22797
Burundi	529	62365

Niger	603	37286
Malawi	1008	32379
Tanzania	1059	40715
Zambia	1139	48782
Kenya	1182	59568
Cote d'Ivoire	1259	51079
Uganda	1589	55899
Congo (Kinshasa)	1904	95634
Liberia	1993	94283
Togo	2199	32985
Nigeria	2851	145924
Ethiopia	3054	155229
Total	26299	1194763

b)

**Country**	**Mean uncertainty interval width (2018)**
Equatorial Guinea	0.870
Lesotho	0.856
Gabon	0.853
Congo	0.818
Madagascar	0.756
Cameroon	0.749
Angola	0.719
South Africa	0.694
Tanzania (Zanzibar)	0.685
Congo, DRC	0.681
Liberia	0.676
Mozambique	0.666
Togo	0.620
Rwanda	0.616
Benin	0.594
Burundi	0.590
Zambia	0.587
Guinea-Bissau	0.573
Tanzania (Mainland)	0.561
Nigeria	0.552
Ethiopia	0.532
Uganda	0.515
Sierra Leone	0.507

Central African	0.475
Guinea	0.463
Somalia	0.463
Swaziland	0.392
Cote d'Ivoire	0.362
Mauritania	0.337
Kenya	0.336
Botswana	0.309
Zimbabwe	0.274
Ghana	0.229
Chad	0.187
Djibouti	0.186
Namibia	0.177
Senegal	0.176
The Gambia	0.149
Malawi	0.139
South Sudan	0.128
Sudan	0.076
Mali	0.068
Burkina Faso	0.060
Niger	0.029
Eritrea	0.013

247

B8. Classification of implementation units (n=5183) within STH spatial limits (6) **according to any STH infection prevalence thresholds** (7) **by year (a), by country in 2018 (b) , exceeding 20% in 2018 and without reported preventative chemotherapy rounds (c), estimated number of children aged 5to14 by prevalence category in 2018 (d), and number of IU's above/below the target 10% prevalence threshold after implementation of ≥5 years of preventive chemotherapy (e).**

a)

Year	STH prevalence category			
	<2%	2-19.9%	20-49.9%	≥50%
2000	30 (1%)	883 (17%)	2022 (39%)	2248 (43%)
2001	47 (1%)	1142 (22%)	2299 (44%)	1695 (33%)
2002	111 (2%)	1707 (33%)	2516 (49%)	849 (16%)
2003	64 (1%)	1374 (27%)	2479 (48%)	1266 (24%)
2004	177 (3%)	1676 (32%)	2526 (49%)	804 (16%)
2005	116 (2%)	1561 (30%)	2516 (49%)	990 (19%)
2006	130 (3%)	1784 (34%)	2500 (48%)	769 (15%)
2007	103 (2%)	1658 (32%)	2496 (48%)	926 (18%)
2008	146 (3%)	1720 (33%)	2464 (48%)	853 (16%)
2009	204 (4%)	2030 (39%)	2348 (45%)	601 (12%)
2010	354 (7%)	2471 (48%)	2042 (39%)	316 (6%)
2011	414 (8%)	2686 (52%)	1853 (36%)	230 (4%)
2012	347 (7%)	2400 (46%)	2066 (40%)	370 (7%)
2013	391 (8%)	2259 (44%)	2115 (41%)	418 (8%)
2014	243 (5%)	2217 (43%)	2203 (43%)	520 (10%)
2015	592 (11%)	2790 (54%)	1623 (31%)	178 (3%)
2016	733 (14%)	3257 (63%)	1103 (21%)	90 (2%)
2017	779 (15%)	3301 (64%)	1017 (20%)	86 (2%)
2018	602 (12%)	3222 (62%)	1233 (24%)	126 (2%)

b)

Country	STH prevalence category				Total IU's
	<2%	2-19.9%	20-49.9%	≥50%	
AGO	0 (0%)	76 (46%)	85 (52%)	3 (2%)	164
BDI	0 (0%)	31 (67%)	15 (33%)	0 (0%)	46
BEN	1 (1%)	61 (79%)	15 (19%)	0 (0%)	77
BFA	52 (74%)	18 (26%)	0 (0%)	0 (0%)	70
BWA	0 (0%)	24 (100%)	0 (0%)	0 (0%)	24

CAF	0 (0%)	16 (94%)	1 (6%)	0 (0%)	17
CIV	0 (0%)	82 (99%)	1 (1%)	0 (0%)	83
CMR	33 (17%)	44 (23%)	102 (54%)	10 (5%)	189
COD	12 (2%)	263 (51%)	228 (44%)	13 (3%)	516
COG	0 (0%)	12 (28%)	30 (70%)	1 (2%)	43
DJI	2 (40%)	3 (60%)	0 (0%)	0 (0%)	5
ERI	58 (100%)	0 (0%)	0 (0%)	0 (0%)	58
ETH	20 (3%)	592 (80%)	121 (16%)	11 (1%)	744
GAB	0 (0%)	0 (0%)	33 (65%)	18 (35%)	51
GHA	35 (16%)	181 (84%)	0 (0%)	0 (0%)	216
GIN	0 (0%)	34 (89%)	4 (11%)	0 (0%)	38
GMB	18 (41%)	24 (55%)	2 (5%)	0 (0%)	44
GNB	3 (3%)	60 (51%)	49 (42%)	6 (5%)	118
GNQ	0 (0%)	0 (0%)	1 (6%)	16 (94%)	17
KEN	34 (12%)	228 (79%)	28 (10%)	0 (0%)	290
LBR	1 (7%)	3 (20%)	7 (47%)	4 (27%)	15
LSO	0 (0%)	0 (0%)	4 (40%)	6 (60%)	10
MDG	0 (0%)	46 (40%)	51 (45%)	17 (15%)	114
MLI	50 (76%)	16 (24%)	0 (0%)	0 (0%)	66
MOZ	0 (0%)	87 (55%)	72 (45%)	0 (0%)	159
MRT	1 (2%)	41 (98%)	0 (0%)	0 (0%)	42
MWI	2 (7%)	27 (93%)	0 (0%)	0 (0%)	29
NAM	13 (38%)	17 (50%)	4 (12%)	0 (0%)	34
NER	32 (82%)	7 (18%)	0 (0%)	0 (0%)	39
NGA	26 (3%)	501 (65%)	234 (30%)	13 (2%)	774
RWA	0 (0%)	17 (57%)	10 (33%)	3 (10%)	30
SDN	123 (78%)	34 (22%)	0 (0%)	0 (0%)	157
SEN	23 (30%)	52 (68%)	1 (1%)	0 (0%)	76
SLE	0 (0%)	11 (79%)	3 (21%)	0 (0%)	14
SOM	1 (6%)	17 (94%)	0 (0%)	0 (0%)	18
SSD	33 (41%)	45 (56%)	2 (3%)	0 (0%)	80
SWZ	0 (0%)	51 (93%)	4 (7%)	0 (0%)	55
TCD	17 (19%)	74 (81%)	0 (0%)	0 (0%)	91
TGO	0 (0%)	29 (73%)	11 (28%)	0 (0%)	40
TZA	7 (4%)	143 (77%)	34 (18%)	2 (1%)	186
TZZ	0 (0%)	6 (55%)	5 (45%)	0 (0%)	11
UGA	4 (3%)	97 (84%)	15 (13%)	0 (0%)	116
ZAF	0 (0%)	21 (40%)	28 (54%)	3 (6%)	52
ZMB	0 (0%)	70 (68%)	33 (32%)	0 (0%)	103
ZWE	1 (2%)	61 (98%)	0 (0%)	0 (0%)	62
Total	602 (12%)	3222 (62%)	1233 (24%)	126 (2%)	5183

c) IU's by country in 2018 with estimated STH prevalence exceeding 20% and with no reported preventative chemotherapy rounds

Country	Freq.	Percent
AGO	55	17.8
BEN	1	0.32
CMR	1	0.32
COD	60	19.42
COG	2	0.65
ETH	7	2.27
GAB	46	14.89
GIN	4	1.29
GMB	2	0.65
GNB	10	3.24
GNQ	17	5.5
KEN	5	1.62
LBR	1	0.32
LSO	5	1.62
MDG	17	5.5
MOZ	1	0.32
NAM	4	1.29
NGA	52	16.83
SSD	2	0.65
TZA	1	0.32
UGA	2	0.65
ZAF	8	2.59
ZMB	6	1.94
Total	309	100

d)

Year	STH prevalence	Estimated population of children 5to14 in 2018 residing in category
2018	<2%	33,716,500
2018	2-19.9%	169,720,524
2018	20-49.9%	60,554,912
2018	≥50%	4,923,187

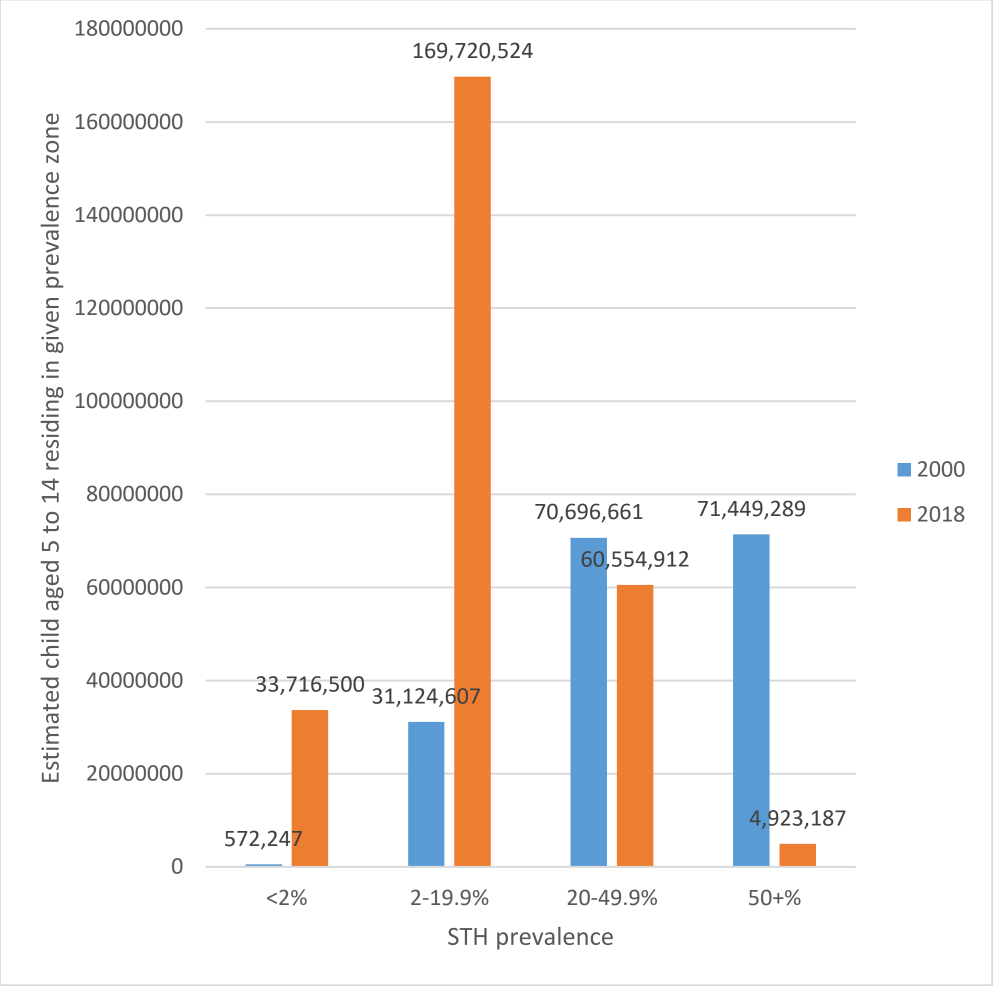

e)

```
. tab five_consecutive_years_MDA sth_post_under10perc

five_conse |
cutive_yea | sth_post_under10perc
    rs_MDA |         0          1 |     Total
-----------+----------------------+----------
         0 |    72,442     25,305 |    97,747
         1 |       399        331 |       730
-----------+----------------------+----------
     Total |    72,841     25,636 |    98,477
```

Number of IU's by country having implemented ≥5 years of preventive chemotherapy and attained a STH prevalence under 10% (N=331):

```
 ADMIN0ISO3 |      Freq.     Percent        Cum.
------------+-----------------------------------
        BDI |         13        3.93        3.93
        BEN |          7        2.11        6.04
        BFA |        126       38.07       44.11
        CMR |          6        1.81       45.92
        ETH |         12        3.63       49.55
        GHA |         94       28.40       77.95
        MLI |         28        8.46       86.40
        MWI |          6        1.81       88.22
        NER |          6        1.81       90.03
```

NGA	20	6.04	96.07
RWA	7	2.11	98.19
SEN	1	0.30	98.49
TGO	4	1.21	99.70
TZA	1	0.30	100.00
Total	331	100.00	

Number of IU's by country having implemented ≥5 years of preventive chemotherapy and NOT attained a STH prevalence under 10% (N=399):

ADMIN0ISO3	Freq.	Percent	Cum.
BDI	31	7.77	7.77
BEN	55	13.78	21.55
BFA	14	3.51	25.06
CMR	25	6.27	31.33
ETH	50	12.53	43.86
GHA	25	6.27	50.13
MDG	15	3.76	53.88
MWI	2	0.50	54.39
NGA	86	21.55	75.94
RWA	21	5.26	81.20
SLE	12	3.01	84.21
TGO	31	7.77	91.98
TZA	14	3.51	95.49
TZZ	18	4.51	100.00
Total	399	100.00	

B9. Classification of implementation units (n=5183) within STH spatial limits (6) according to moderate-to-heavy intensity STH infection prevalence thresholds (7) by year (a) by country in 2018 (b) and change in estimated number of school aged children by STH moderate-to-heavy intensity STH infection prevalence thresholds from 2000 to 2018

a)

Year	STH moderate-to-heavy intensity prevalence category				
	**<2%**	**2-4.9%**	**5-9.9%**	**10-14.9%**	**≥15%**
2000	946 (18 %)	1048 (20 %)	1196 (23 %)	841 (16 %)	1152 (22 %)
2001	1218 (23 %)	1171 (23 %)	1348 (26 %)	671 (13 %)	775 (15 %)
2002	1873 (36 %)	1424 (27 %)	1183 (23 %)	378 (7 %)	325 (6 %)
2003	1485 (29 %)	1260 (24 %)	1371 (26 %)	538 (10 %)	529 (10 %)
2004	1898 (37 %)	1478 (29 %)	1147 (22 %)	360 (7 %)	300 (6 %)
2005	1730 (33 %)	1339 (26 %)	1283 (25 %)	429 (8 %)	402 (8 %)
2006	1972 (38 %)	1451 (28 %)	1133 (22 %)	334 (6 %)	293 (6 %)
2007	1814 (35 %)	1401 (27 %)	1199 (23 %)	394 (8 %)	375 (7 %)
2008	1923 (37 %)	1414 (27 %)	1155 (22 %)	356 (7 %)	335 (6 %)
2009	2277 (44 %)	1458 (28 %)	952 (18 %)	278 (5 %)	218 (4 %)
2010	2867 (55 %)	1474 (28 %)	593 (11 %)	154 (3 %)	95 (2 %)
2011	3153 (61 %)	1371 (26 %)	474 (9 %)	115 (2 %)	70 (1 %)
2012	2809 (54 %)	1434 (28 %)	646 (12 %)	171 (3 %)	123 (2 %)
2013	2699 (52 %)	1418 (27 %)	718 (14 %)	203 (4 %)	145 (3 %)
2014	2512 (48 %)	1406 (27 %)	827 (16 %)	243 (5 %)	195 (4 %)
2015	3442 (66 %)	1236 (24 %)	361 (7 %)	85 (2 %)	59 (1 %)
2016	4045 (78 %)	844 (16 %)	223 (4 %)	45 (1 %)	26 (1 %)
2017	4132 (80 %)	785 (15 %)	201 (4 %)	46 (1 %)	19 (0 %)
2018	3882 (75 %)	956 (18 %)	251 (5 %)	60 (1 %)	34 (1 %)

b)

Country	STH moderate-to-heavy intensity prevalence category					Total
	<2%	2-4.9%	5-9.9%	10-14.9%	≥15%	
AGO	84 (51), (2)	68 (41), (7)	9 (5), (4)	1 (1), (2)	2 (1), (6)	164 (100), (3)
BDI	32 (70), (1)	14 (30), (1)	0 (0), (0)	0 (0), (0)	0 (0), (0)	46 (100), (1)
BEN	63 (82), (2)	14 (18), (1)	0 (0), (0)	0 (0), (0)	0 (0), (0)	77 (100), (1)
BFA	70 (100), (2)	0 (0), (0)	0 (0), (0)	0 (0), (0)	0 (0), (0)	70 (100), (1)
BWA	24 (100), (1)	0 (0), (0)	0 (0), (0)	0 (0), (0)	0 (0), (0)	24 (100), (0)
CAF	16 (94), (0)	1 (6), (0)	0 (0), (0)	0 (0), (0)	0 (0), (0)	17 (100), (0)
CIV	83 (100), (2)	0 (0), (0)	0 (0), (0)	0 (0), (0)	0 (0), (0)	83 (100), (2)
CMR	80 (42), (2)	79 (42), (8)	22 (12), (9)	7 (4), (12)	1 (1), (3)	189 (100), (4)

COD	282 (55), (7)	184 (36), (19)	43 (8), (17)	7 (1), (12)	0 (0), (0)	516 (100), (10)
COG	12 (28), (0)	17 (40), (2)	14 (33), (6)	0 (0), (0)	0 (0), (0)	43 (100), (1)
DJI	5 (100), (0)	0 (0), (0)	0 (0), (0)	0 (0), (0)	0 (0), (0)	5 (100), (0)
ERI	58 (100), (1)	0 (0), (0)	0 (0), (0)	0 (0), (0)	0 (0), (0)	58 (100), (1)
ETH	621 (83), (16)	97 (13), (10)	18 (2), (7)	8 (1), (13)	0 (0), (0)	744 (100), (14)
GAB	1 (2), (0)	7 (14), (1)	32 (63), (13)	8 (16), (13)	3 (6), (9)	51 (100), (1)
GHA	216 (100), (6)	0 (0), (0)	0 (0), (0)	0 (0), (0)	0 (0), (0)	216 (100), (4)
GIN	34 (89), (1)	4 (11), (0)	0 (0), (0)	0 (0), (0)	0 (0), (0)	38 (100), (1)
GMB	42 (95), (1)	2 (5), (0)	0 (0), (0)	0 (0), (0)	0 (0), (0)	44 (100), (1)
GNB	63 (53), (2)	36 (31), (4)	16 (14), (6)	1 (1), (2)	2 (2), (6)	118 (100), (2)
GNQ	0 (0), (0)	0 (0), (0)	1 (6), (0)	7 (41), (12)	9 (53), (26)	17 (100), (0)
KEN	267 (92), (7)	23 (8), (2)	0 (0), (0)	0 (0), (0)	0 (0), (0)	290 (100), (6)
LBR	5 (33), (0)	4 (27), (0)	3 (20), (1)	0 (0), (0)	3 (20), (9)	15 (100), (0)
LSO	0 (0), (0)	0 (0), (0)	4 (40), (2)	5 (50), (8)	1 (10), (3)	10 (100), (0)
MDG	47 (41), (1)	34 (30), (4)	18 (16), (7)	7 (6), (12)	8 (7), (24)	114 (100), (2)
MLI	66 (100), (2)	0 (0), (0)	0 (0), (0)	0 (0), (0)	0 (0), (0)	66 (100), (1)
MOZ	89 (56), (2)	70 (44), (7)	0 (0), (0)	0 (0), (0)	0 (0), (0)	159 (100), (3)
MRT	42 (100), (1)	0 (0), (0)	0 (0), (0)	0 (0), (0)	0 (0), (0)	42 (100), (1)
MWI	29 (100), (1)	0 (0), (0)	0 (0), (0)	0 (0), (0)	0 (0), (0)	29 (100), (1)
NAM	30 (88), (1)	4 (12), (0)	0 (0), (0)	0 (0), (0)	0 (0), (0)	34 (100), (1)
NER	39 (100), (1)	0 (0), (0)	0 (0), (0)	0 (0), (0)	0 (0), (0)	39 (100), (1)
NGA	536 (69), (14)	176 (23), (18)	52 (7), (21)	6 (1), (10)	4 (1), (12)	774 (100), (15)
RWA	17 (57), (0)	10 (33), (1)	0 (0), (0)	2 (7), (3)	1 (3), (3)	30 (100), (1)
SDN	157 (100), (4)	0 (0), (0)	0 (0), (0)	0 (0), (0)	0 (0), (0)	157 (100), (3)
SEN	75 (99), (2)	1 (1), (0)	0 (0), (0)	0 (0), (0)	0 (0), (0)	76 (100), (1)
SLE	11 (79), (0)	3 (21), (0)	0 (0), (0)	0 (0), (0)	0 (0), (0)	14 (100), (0)
SOM	18 (100), (0)	0 (0), (0)	0 (0), (0)	0 (0), (0)	0 (0), (0)	18 (100), (0)
SSD	78 (98), (2)	2 (3), (0)	0 (0), (0)	0 (0), (0)	0 (0), (0)	80 (100), (2)
SWZ	52 (95), (1)	3 (5), (0)	0 (0), (0)	0 (0), (0)	0 (0), (0)	55 (100), (1)
TCD	91 (100), (2)	0 (0), (0)	0 (0), (0)	0 (0), (0)	0 (0), (0)	91 (100), (2)
TGO	29 (73), (1)	11 (28), (1)	0 (0), (0)	0 (0), (0)	0 (0), (0)	40 (100), (1)
TZA	152 (82), (4)	27 (15), (3)	7 (4), (3)	0 (0), (0)	0 (0), (0)	186 (100), (4)
TZZ	9 (82), (0)	1 (9), (0)	1 (9), (0)	0 (0), (0)	0 (0), (0)	11 (100), (0)
UGA	102 (88), (3)	13 (11), (1)	1 (1), (0)	0 (0), (0)	0 (0), (0)	116 (100), (2)
ZAF	22 (42), (1)	19 (37), (2)	10 (19), (4)	1 (2), (2)	0 (0), (0)	52 (100), (1)
ZMB	71 (69), (2)	32 (31), (3)	0 (0), (0)	0 (0), (0)	0 (0), (0)	103 (100), (2)
ZWE	62 (100), (2)	0 (0), (0)	0 (0), (0)	0 (0), (0)	0 (0), (0)	62 (100), (1)
Total	3882 (75), (100)	956 (18), (100)	251 (5), (100)	60 (1), (100)	34 (1), (100)	5183 (100), (100)

c) Estimated child population counts (5-14 years) by change in intensity infection category from 2000 to 2018

STH cat [i] 2000:2018	Total population	Total population	Sum, 2018	Proportion, 2018

	(5 to 14), 2000	(5 to 14), 2018		
01:01	32,849,816	50,816,069	206,204,822	76.7%
02:01	35,181,375	54,414,795		
03:01	42,574,678	65,862,106		
04:01	18,846,441	29,154,094		
05:01	3,851,143	5,957,758		
02:02	27	42	62,710,301	23.3%
03:02	1,118,003	1,729,219		
04:02	8,786,572	13,591,188		
05:02	20,578,010	31,830,726		
04:03	13,330	20,621		
05:03	7,662,719	11,854,222		
05:04	1,529,323	2,366,044		
05:05	851,367	1,318,239		
Total	173,842,804	268,915,123		

i STH cat: 1: <2% moderate-to-heavy intensity; 2: 2-4.9%; 3: 5-9.9%; 4: 10-14.9%; 5: 15+%

B10. Full statistical model specification

The input data were as follows: O_{1ij}, O_{2ij} and O_{3ij} and n_{1ij}, n_{2ij} and n_{3ij} represent the observed number of cases of and number tested for HK, ASC and TT respectively, for implementation unit (IU) i = 1, ..., 5183 and year j = 2000, …, 2018.Specifically, the observed numbers of positives (O_1=HK, O_2=ASC, O_3=TT) scaled by the total number tested (n_1, n_2, n_3) in each unit i in year j is assumed to follow a binomial distribution with prevalence proportions ρ_{1ij}, ρ_{2ij} and ρ_{3ij}.

$O_{1ij} \sim \text{Binomial}(\rho_{1ij}, n_{1ij})$	$O_{2ij} \sim \text{Binomial}(\rho_{2ij}, n_{2ij})$	$O_{3ij} \sim \text{Binomial}(\rho_{3ij}, n_{3ij})$
$\text{logit}(\rho_{1ij}) = \alpha_1 + X_i\beta_{1(1)} + X_{it}\beta_{1(2)} + \mu_{1ij}$	$\text{logit}(\rho_{2ij}) = \alpha_2 + X_i\beta_{2(1)} + X_{it}\beta_{2(2)} + \mu_{2ij}$	$\text{logit}(\rho_{3ij}) = \alpha_3 + X_i\beta_{3(1)} + X_{it}\beta_{3(2)} + \mu_{3ij}$

where $p_{(1\text{-}3)it}$ corresponds to the prevalence rate for a given STH and $n_{(1\text{-}3)it}$ is the total number of tested individuals in area i in year j, α's are the disease-specific intercepts, Xi and Xi$_t$ are the time in varying and time varying covariates respectively and $\beta_{1\text{-}3}$ are the vector of regression coefficients for each STH species. The space–time structure is introduced on the logit scale through the terms: μ_{1ij}, μ_{2ij} and μ_{3ij}.

In this analysis we modelled the joint spatial-temporal structure as follows and in accordance with previous suggested formulations (8, 9):

$$\mu_{1ij} = \lambda_{1i}\delta_1 + \lambda_{2i} + \xi_{1j}\kappa_1 + \xi_{2j} + v_{ij}$$

$$\mu_{2ij} = \lambda_{1i}\delta_2 + \lambda_{3i} + \xi_{1j}\kappa_2 + \xi 3j + v_{ij}$$

$$\mu_{3ij} = \lambda_{1i}\delta_3 + \lambda_{4i} + \xi_{1j}\kappa_3 + \xi 4j + v_{ij}$$

where λ_{1i} and ξ_{1j} represent the shared spatial and temporal patterns respectively; λ_{2i}, λ_{3i}, λ_{4i} represent the differential spatial pattern from the shared spatial pattern for each STH respectively; ξ_{2j}, ξ_{3j}, ξ_{4j} represent the differential temporal pattern from the shared temporal pattern for each STH respectively and v_{ij} is the space–time order 2 interaction term. The spatial structure (λ) was modelled using a conditional autoregressive Gaussian distribution (CAR) i.e. the conditional

distribution of each λ_i given all λ's is a normal distribution with mean equal to the average of the λ 's of its neighbouring polygons, and precision proportional to the number of 'neighbours'. A first order queen contingency (i.e. all surrounding IU's which share or touch the boundary of a given IU) neighbourhood structure was utilised and utilised in the CAR via matrix W. We assumed a first order random walk for the temporal effects (ξ) or one dimensional versions of the CAR spatial priors, with weight matrices Q that define the temporal neighbours of year j as years j − 1 and j + 1 (with a single neighbour in the first and last year in the series namely years j = 2000 and j = 2018). The scaling parameters (δ and κ) represent the relative contribution of the shared terms to the risk of given STH species to the overall STH spatial and temporal effects respectively. We implemented a sum to zero constraint for $\delta_{1\text{-}3}$ and $\kappa_{1\text{-}3}$ to ensure model identifiability (10).

Lastly, we assumed an exchangeable (unstructured) hierarchical structure for the shared interaction terms ν_{ij}.

For the regression coefficients (β), we choose non-informative normal prior distributions. We followed previous recommendations for the precision parameters of the spatial and temporal CAR priors (11)namely:

α's $\propto 1$ (for identifiability)

λ's $\sim$ CARNormal(W, $\tau\lambda$)

ξ's $\sim$ CARNormal(Q, $\tau\xi$)

$\nu_{ij} \sim$ Normal(0, $\tau\nu$)

τ s $\sim$ Gamma(0.5, 0.0005)

log δ, log $\kappa \sim$ Normal(0, 5.9)

B11. WinBUGS code for model implementation

```
model
{
for (i in 1:N) {
	for (j in 1:T) {

#Binomial likelihood
	Y1b[i,j]~dbin(p1[i,j],Y1m_tot[i,j])
	Y2b[i,j]~dbin(p2[i,j],Y2m_tot[i,j])
	Y3b[i,j]~dbin(p3[i,j],Y3m_tot[i,j])
	Y4b[i,j]~dbin(p4[i,j],Y4m_tot[i,j])

#Risk factor model
	logit(p1[i,j])<-b1[1]*x1s[i]+b2[1]*x2s[i]+b3[1]*x3s[i,j]+b4[1]*x4s[i,j]+b5[1]*x5s[i,j]+mu[i,j,1]
	logit(p2[i,j])<-b1[2]*x1s[i]+b2[2]*x2s[i]+b3[2]*x3s[i,j]+b4[2]*x4s[i,j]+b5[2]*x5s[i,j]+mu[i,j,2]
	logit(p3[i,j])<-b1[3]*x1s[i]+b2[3]*x2s[i]+b3[3]*x3s[i,j]+b4[3]*x4s[i,j]+b5[3]*x5s[i,j]+mu[i,j,3]
	logit(p4[i,j])<-b1.4*x1s[i]+b2.4*x2s[i]+b3.4*x3s[i,j]+b4.4*x4s[i,j]+b5.4*x5s[i,j]+mu[i,j,4]

	mu[i,j,1:4]~dmnorm(eta[i,j,],Sigma.inv[,])

#Joint modelling
	eta[i,j,1]<-phi1[i]*delta[1]+gamma1[j]*kappa[1]+nu1[i,j]
	eta[i,j,2]<-phi2[i]*delta[2]+gamma2[j]*kappa[2]+nu2[i,j]
	eta[i,j,3]<-phi3[i]*delta[3]+gamma3[j]*kappa[3]+nu3[i,j]
	eta[i,j,4]<-phi1[i]/delta[1]+ phi2[i]/delta[2]+ phi3[i]/delta[3] +gamma1[j]/kappa[1] +gamma2[j]/kappa[2]+
	gamma3[j]/kappa[3]+nu4[i,j]

	}

#Baseline and endline prevalence and exceedance probability posteriors
sth2000[i]<-p4[i,1]
sth2018[i]<-p4[i,19]

hk2000[i]<-p1[i,1]
hk2018[i]<-p1[i,19]

asc2000[i]<-p2[i,1]
asc2018[i]<-p2[i,19]

tt2000[i]<-p3[i,1]
tt2018[i]<-p3[i,19]

exc_p_low_2000[i]<-step(0.1-p4[i,1])
exc_p_low_2018[i]<-step(0.1-p4[i,19])

exc_p_high_2000[i]<-step(0.2-p4[i,1])
exc_p_high_2018[i]<-step(0.2-p4[i,19])

}

#Spatial Modelling (priors)
phi1[1:N]~car.normal(adj[],weights[],num[],tau.phi[1])
phi2[1:N]~car.normal(adj[],weights[],num[],tau.phi[2])
phi3[1:N]~car.normal(adj[],weights[],num[],tau.phi[3])

#Weights for adjacency matrices in space
for(k in 1:sumNumNeigh) {
	weights[k]<-1
```

```
        }

#Temporal Modelling (priors)
gamma1[1:T]~car.normal(adj.t[],weights.t[],num.t[],tau.gamma[1])
gamma2[1:T]~car.normal(adj.t[],weights.t[],num.t[],tau.gamma[2])
gamma3[1:T]~car.normal(adj.t[],weights.t[],num.t[],tau.gamma[3])

for(t in 1:1){
        weights.t[t] <- 1;
        adj.t[t] <- t+1;
        num.t[t] <- 1
       }
 for(t in 2:(T-1)) {
        weights.t[2+(t-2)*2] <- 1;
        adj.t[2+(t-2)*2] <- t-1
        weights.t[3+(t-2)*2] <- 1;
        adj.t[3+(t-2)*2] <- t+1;
        num.t[t] <- 2
       }
for(t in T:T) {
       weights.t[(T-2)*2 + 2] <- 1;
       adj.t[(T-2)*2 + 2] <- t-1;
       num.t[t] <- 1
       }

#Space-time Interaction Modelling (priors)
for(i in 1:N){
        for(j in 1:T){
                nu1[i,j]~dnorm(alpha1, tau.nu[1])
                nu2[i,j]~dnorm(alpha2, tau.nu[2])
                nu3[i,j]~dnorm(alpha3, tau.nu[3])
                nu4[i,j]~dnorm(alpha4, tau.nu[4])
                }
}

#Hyperprior specification

for(k in 1:3) {
        tau.phi[k]~dgamma(0.5, 0.005)
}

for(k in 1:3) {
        tau.gamma[k]~dgamma(0.5, 0.005)
}

for(k in 1:4) {
        tau.nu[k]~dgamma(0.5, 0.005)
}

for(k in 1:3) {
        delta[k]~dunif(0.575,1.675)
        kappa[k]~dunif(0.575,1.675)
}

#Intercepts
alpha1~dflat()
alpha2~dflat()
alpha3~dflat()
alpha4~dflat()
```

```
Sigma.inv[1:4,1:4]~dwish(B[,],4)
B[1,1]<-0.01
B[2,2]<-0.01
B[3,3]<-0.01
B[4,4]<-0.01
B[1,2]<-0
B[1,3]<-0
B[1,4]<-0
B[2,1]<-0
B[2,3]<-0
B[2,4]<-0
B[3,1]<-0
B[3,2]<-0
B[3,4]<-0
B[4,1]<-0
B[4,2]<-0
B[4,3]<-0

#Coefficient for covariates

for(k in 1:3) {
        b1[k]~dnorm(0,0.01)
        b2[k]~dnorm(0,0.01)
        b3[k]~dnorm(0,0.01)
        b4[k]~dnorm(0,0.01)
        b5[k]~dnorm(0,0.01)
}

b1.4<-(b1[1]+b1[2]+b1[3])/3
b2.4<-(b2[1]+b2[2]+b2[3])/3
b3.4<-(b3[1]+b3[2]+b3[3])/3
b4.4<-(b4[1]+b4[2]+b4[3])/3
b5.4<-(b5[1]+b5[2]+b5[3])/3

for (i in 1:N) {
        for (j in 1:T) {
                HKtot[i,j]<-p1[i ,j]*pop[i,j]
                ASCtot[i,j]<-p2[i ,j]*pop[i,j]
                TTtot[i,j]<-p3[i ,j]*pop[i,j]
                STHtot[i,j]<-p4[i ,j]*pop[i,j]
                }
}

for (j in 1 : T) {
        HKprev[j] <- sum(HKtot[,j])/sum(pop[,j])
        ASCprev[j] <-  sum(ASCtot[,j])/sum(pop[,j])
        TTprev[j] <-  sum(TTtot[,j])/sum(pop[,j])
        STHprev[j] <-  sum(STHtot[,j])/sum(pop[,j])
        }

}
```

B12. Gelman-Rubin convergence plots for key model parameters

Model convergence was assessed by visual inspection of the series plot of each parameter, and using Gelman-Rubin statistics (12). Furthermore, the final posterior samples were also assessed to check if the Monte Carlo error for each parameter was less than 5% of the sample standard deviation. An assessment of model convergence using Gelman-Rubin statistics/plots (Figure B12) are presented below. An inspection of these plots suggested convergence/stabilisation of the full multivariable space-time model after approximately 25,000 iterations.

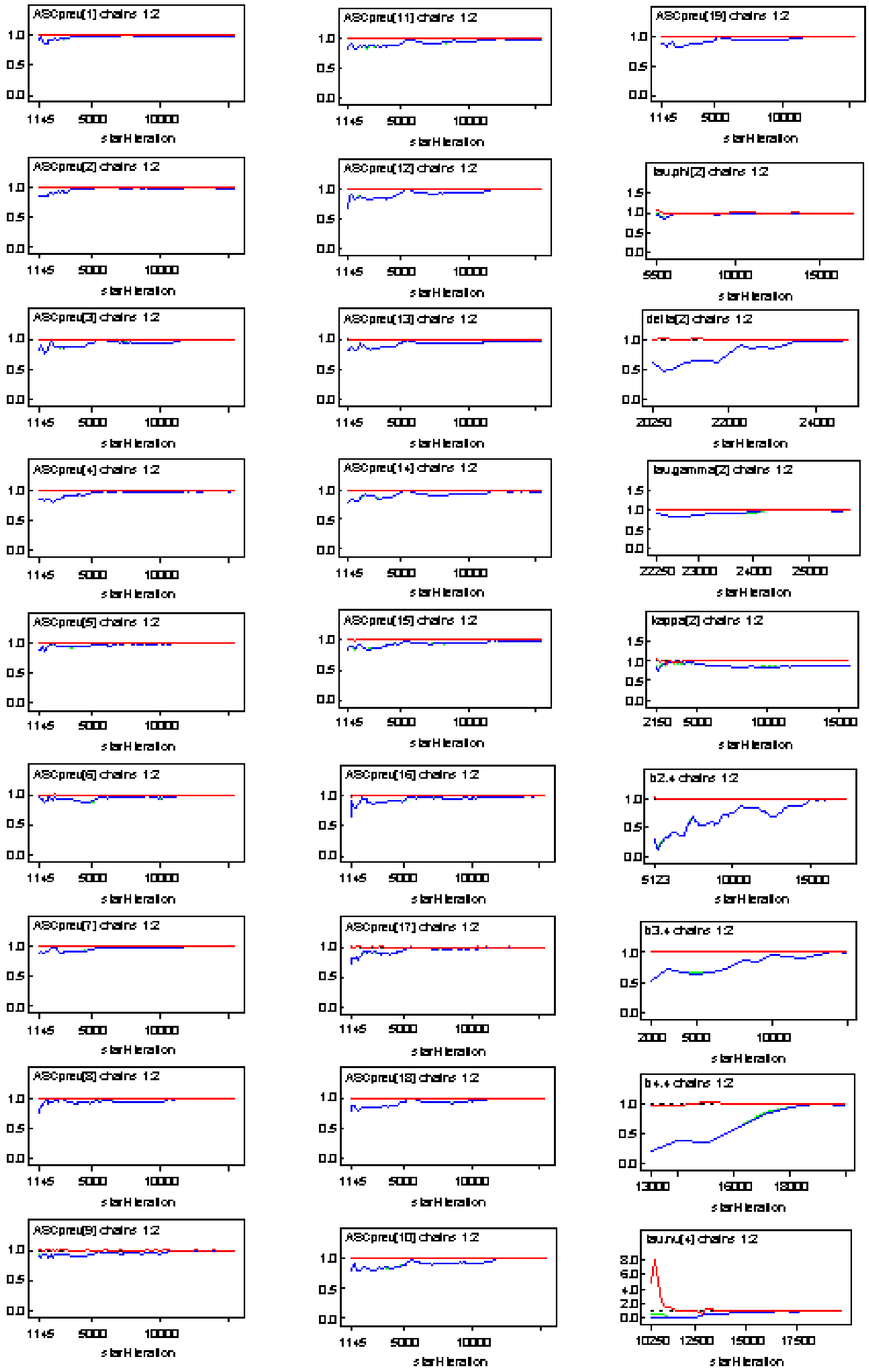

Figure B12: Gelman-Rubin statistics plots for key model parameters as confirmation of convergence

B13. Scatter plots and correlation coefficients for observed prevalence versus model posterior predicted prevalence

A comparison of observed versus fitted STH prevalence overall and by sub-species (see scatter plots below, Figure B13) from the full model suggested a very high degree of correlation (STH: spearman rho=0.995, P<0.001; hookworm: spearman rho=0.967, P<0.001; Ascaris: spearman rho=0.951, P<0.001; Trichuris: spearman rho=0.995, P<0.001) with a few notable differences e.g. many data points with observed prevalence of zero were corrected upwards by the model and/or smoothed towards local areal mean based on contiguity matrix in space and time.

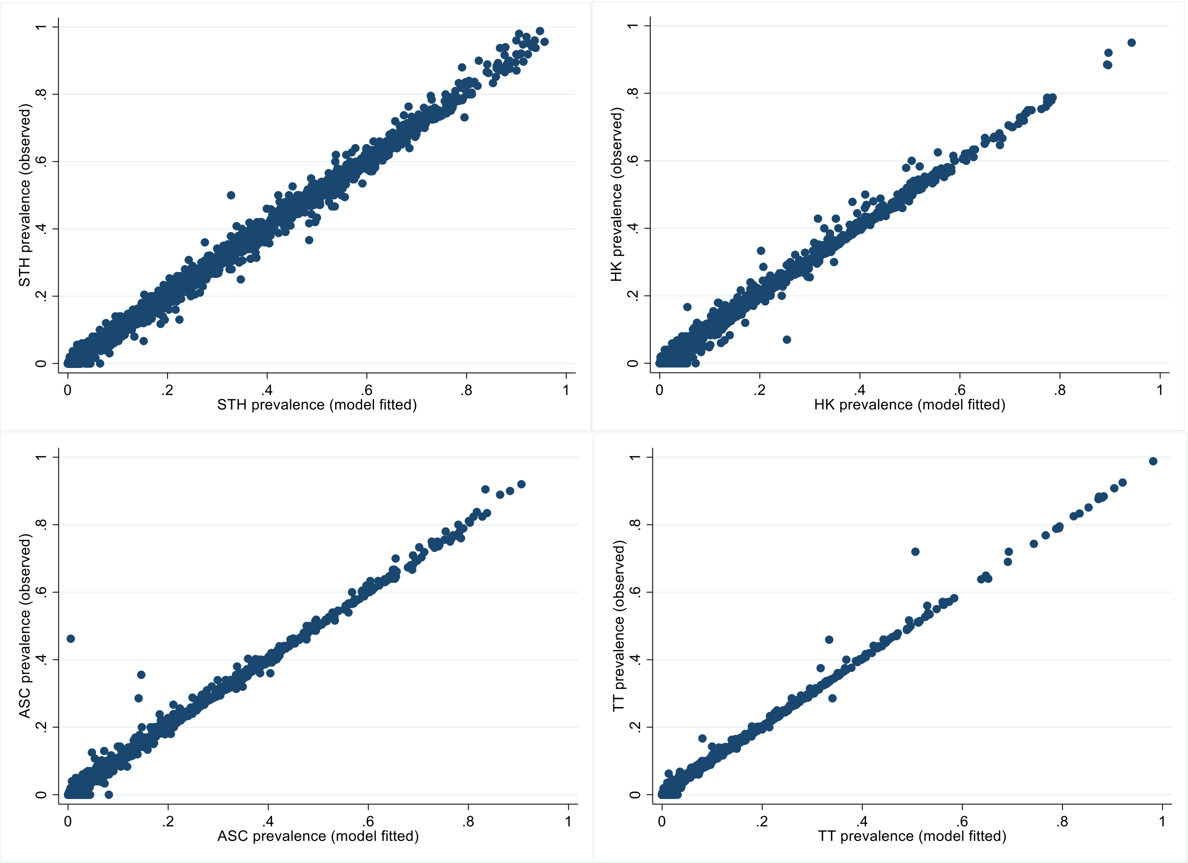

Figure B13.1: Scatterplots comparing observed versus model fitted prevalence by species for the full models.

We also compared agreement between the observed and model fitted prevalence using the Bland-Altman method (Figure B13.2). This method suggests a high concordance (Lin's Concordance Correlation coeff. of Absolute Agreement = 0.9979) with only 5.5% of observations outside the limits of agreement. This agreement was similar for the predictions for the individual species (please see below).

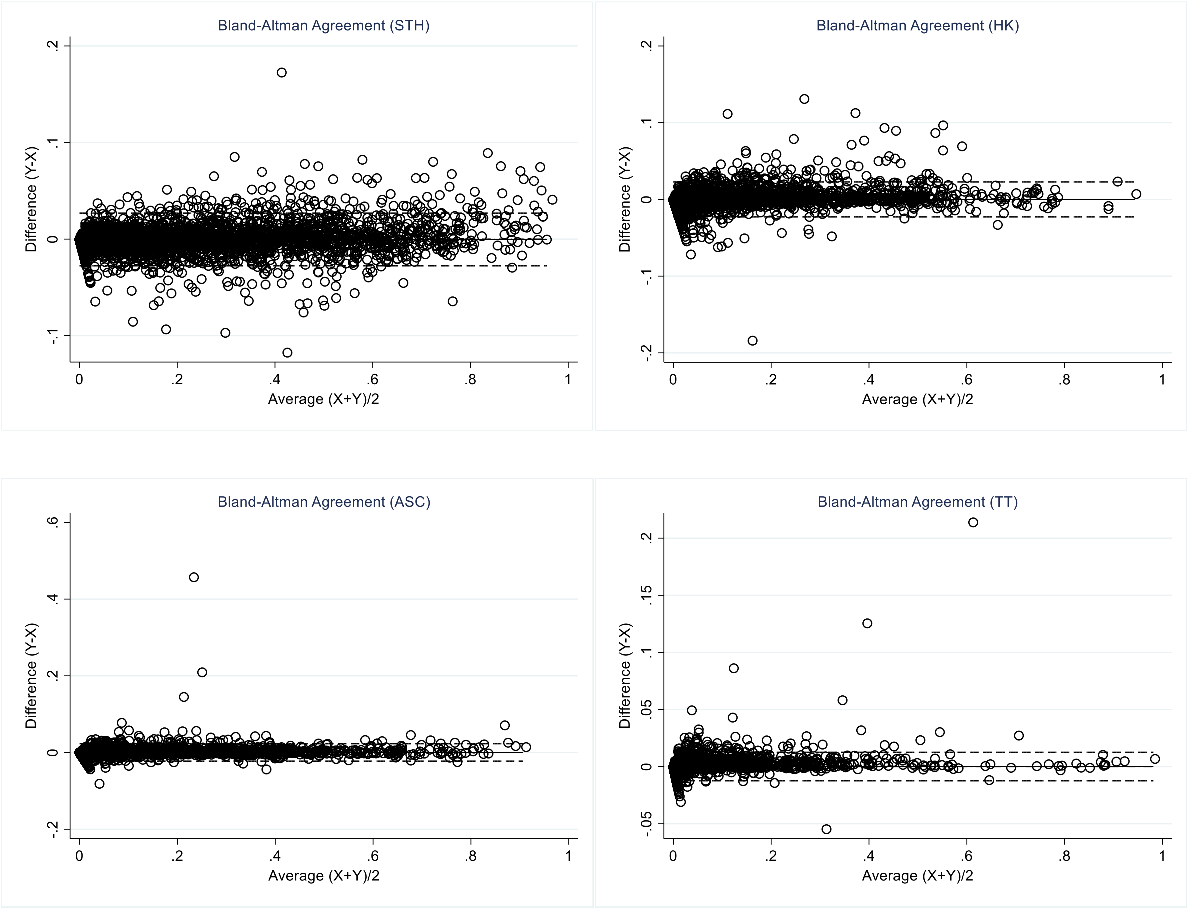

Figure B13.2: Bland-Altman plots for observed versus model fitted prevalence by species with limits of agreement (LoA).

STH

```
Bland-Altman: Absolute values of Bias & Limits of Agreement (LoA)
--------------------------------------------------------------------------
Parameter             Estimate  Std. Dev.   Std. Err.  [95% Conf. Interval]
Diff. (Y-X):   Bias  -.0003533   .0139362    .0002165   -.0007778  .0000711
          Lower LoA  -.0276678                .000375    -.028403 -.0269327
          Upper LoA   .0269611                .000375     .026226  .0276963
--------------------------------------------------------------------------
Cases over limit = 138 (3.33%)
Cases under limit = 92 (2.22%)
Spearman correlation between (Y-X) and (X+Y)/2: r=  0.1949 (p= 0.0000)
Lin's Concordance Correlation coeff. of Absolute Agreement = 0.9979
--------------------------------------------------------------------------
```

HK

```
Bland-Altman: Absolute values of Bias & Limits of Agreement (LoA)
--------------------------------------------------------------------------
Parameter             Estimate  Std. Dev.   Std. Err.  [95% Conf. Interval]
Diff. (Y-X):   Bias  -.0000311   .0116327     .000186   -.0003958  .0003336
          Lower LoA  -.0228308               .0003222   -.0234625 -.0221991
          Upper LoA   .0227686               .0003222    .0221369  .0234003
--------------------------------------------------------------------------
Cases over limit = 116 (2.97%)
Cases under limit = 80 (2.05%)
Spearman correlation between (Y-X) and (X+Y)/2: r=  0.3465 (p= 0.0000)
Lin's Concordance Correlation coeff. of Absolute Agreement = 0.9966
--------------------------------------------------------------------------
```

ASC

```
Bland-Altman: Absolute values of Bias & Limits of Agreement (LoA)
```

```
-----------------------------------------------------------------------------
Parameter             Estimate  Std. Dev.   Std. Err.  [95% Conf. Interval]
Diff. (Y-X):   Bias   .0002786   .0115271    .0001841   -.0000824  .0006396
         Lower LoA   -.022314                .0003189   -.0229393 -.0216888
         Upper LoA   .0228713                .0003189     .022246  .0234966
-----------------------------------------------------------------------------
Cases over limit = 74 (1.89%)
Cases under limit = 37 (0.94%)
Spearman correlation between (Y-X) and (X+Y)/2: r=  0.4013 (p= 0.0000)
Lin's Concordance Correlation coeff. of Absolute Agreement = 0.9970
-----------------------------------------------------------------------------
```

TT

```
Bland-Altman: Absolute values of Bias & Limits of Agreement (LoA)
-----------------------------------------------------------------------------
Parameter             Estimate  Std. Dev.   Std. Err.  [95% Conf. Interval]
Diff. (Y-X):   Bias    .000109   .0063809    .0001033   -.0000935  .0003114
         Lower LoA  -.0123974                .0001788   -.0127481 -.0120468
         Upper LoA   .0126153                .0001788    .0122647   .012966
-----------------------------------------------------------------------------
Cases over limit = 77 (2.02%)
Cases under limit = 42 (1.10%)
Spearman correlation between (Y-X) and (X+Y)/2: r=  0.4308 (p= 0.0000)
Lin's Concordance Correlation coeff. of Absolute Agreement = 0.9979
-----------------------------------------------------------------------------
```

B14. Out of sample validation

A random 20% of observed data points were drawn for STH overall and for each sub-species separately from the space-time cube. The data with these points removed were then re-inputted into WinBUGS. The posterior distributions for the predicted prevalence for these 20% of removed data points were then compared against the observed values to ascertain the predictive power of the model (i.e. out of sample validation). The percentage of observed prevalence values that were contained within the credibility interval of the posterior distribution for the predicted prevalence were calculated. Of note is that 742/752 (or 99%%) of the observed prevalence values were contained in the 95% credible interval (CI) for its posterior distribution while 507/752 or 67% where contained within the 50% credible interval (i.e. 25% to 75% centile). The scatter plot comparing observed versus model fitted prevalence for validation sample suggests highly significant moderate strength correlation (Spearman rank correlation coefficient +0.67, p-value <0.001) (please see Figure B14 below).

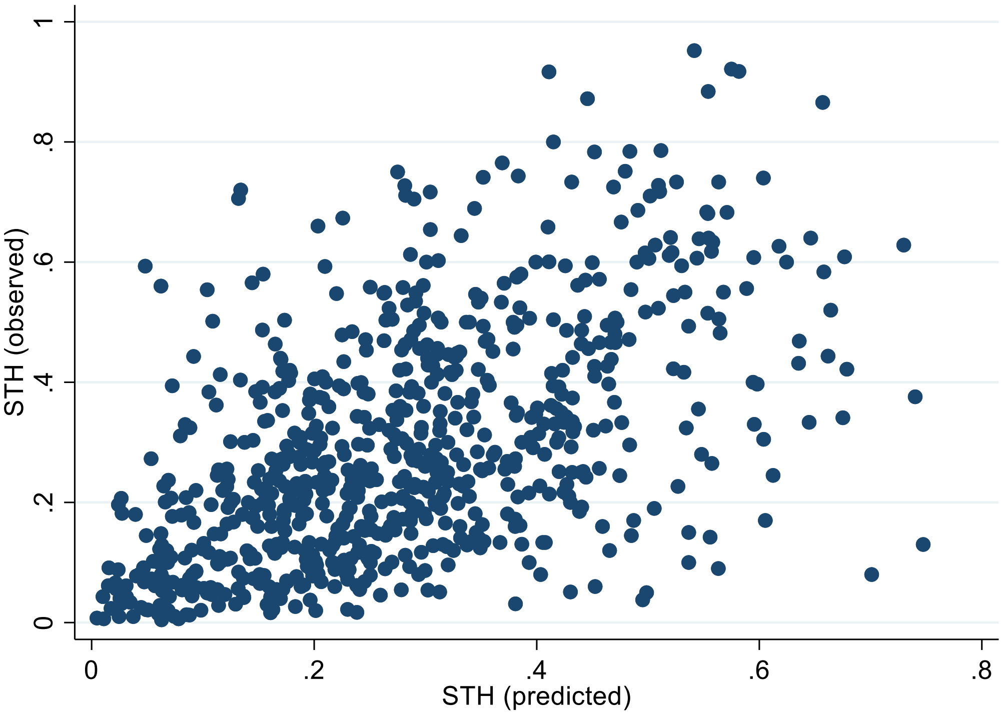

Figure B14: Scatter plot comparing observed versus model predicted for the out of sample validation

B15. World Health Organization preventive chemotherapy guidelines to control soil-transmitted helminth infections (reduce worm burden and thus morbidity) in at-risk population groups (13)

Preventive chemotherapy (or deworming), using annual or biannual (biannual administration recommended where the baseline prevalence is over 50%) single-dose albendazole (400 mg) or mebendazole (500 mg) (half-dose of albendazole (i.e. 200 mg) is recommended for children under 2 years of age) is recommended as a public health intervention for all young children (12-23 months of age), preschool (24-59 months of age) and school-age children (5 and 12 years of age) living in areas where the baseline prevalence of any soil-transmitted infection is 20% or higher among children (*strong recommendation, low-quality evidence*).

Preventive chemotherapy using annual or biannual (biannual administration recommended where the baseline prevalence is over 50%) single-dose albendazole (400 mg) or mebendazole (500 mg), is recommended for all non-pregnant adolescent girls (10–19 years of age) and non-pregnant women of reproductive age (15–49 years of age) residing in areas where the baseline prevalence of any soil-transmitted helminth infection is 20% or higher among non-pregnant adolescent girls and/or non-pregnant women of reproductive age (*strong recommendation, moderate-quality evidence*).

Preventive chemotherapy (deworming), using single-dose albendazole (400 mg) or mebendazole (500 mg), is recommended for pregnant women, after the first trimester, living in areas where the baseline prevalence of hookworm and/or T. trichiura infection is 20% or higher among pregnant women, AND where anaemia is a severe public health problem, with a prevalence of 40% or higher among pregnant women (*conditional recommendation, moderate-quality evidence*).

References

1. Hijmans RJ, Cameron SE, Parra JL, Jones PG, Jarvis A. Very high resolution interpolated climate surfaces for global land areas. International Journal of Climatology: A Journal of the Royal Meteorological Society. 2005;25(15):1965-78.
2. Hengl T, de Jesus JM, Heuvelink GB, Gonzalez MR, Kilibarda M, Blagotić A, et al. SoilGrids250m: Global gridded soil information based on machine learning. PLoS one. 2017;12(2):e0169748.
3. Tusting LS, Bisanzio D, Alabaster G, Cameron E, Cibulskis R, Davies M, et al. Mapping changes in housing in sub-Saharan Africa from 2000 to 2015. Nature. 2019:1.
4. Kummu M, Taka M, Guillaume JH. Gridded global datasets for gross domestic product and Human Development Index over 1990–2015. Scientific data. 2018;5:180004.
5. Fick SE, Hijmans RJ. WorldClim 2: new 1-km spatial resolution climate surfaces for global land areas. International journal of climatology. 2017;37(12):4302-15.
6. Pullan RL, Brooker SJ. The global limits and population at risk of soil-transmitted helminth infections in 2010. Parasites & vectors. 2012;5(1):81.
7. Organization WH. Soil-transmitted helminthiases: eliminating as public health problem soil-transmitted helminthiases in children: progress report 2001-2010 and strategic plan 2011-2020. 2012.
8. Knorr-Held L, Best NG. A shared component model for detecting joint and selective clustering of two diseases. Journal of the Royal Statistical Society: Series A (Statistics in Society). 2001;164(1):73-85.
9. Richardson S, Abellan JJ, Best N. Bayesian spatio-temporal analysis of joint patterns of male and female lung cancer risks in Yorkshire (UK). Statistical methods in medical research. 2006;15(4):385-407.
10. MacNab YC. On Bayesian shared component disease mapping and ecological regression with errors in covariates. Statistics in medicine. 2010;29(11):1239-49.
11. Elliott P, Wakefield JC, Best NG, Briggs DJ. Spatial epidemiology: methods and applications: Oxford University Press Oxford; 2000.
12. Gelman A, Rubin DB. Inference from iterative simulation using multiple sequences. Statistical science. 1992;7(4):457-72.
13. World Health Organization. Guideline: preventive chemotherapy to control soil-transmitted helminth infections in at-risk population groups: World Health Organization; 2017.
